# Design and Synthesis of Pleuromutilin Derivatives as Antibacterial Agents Using Quantitative Structure–Activity Relationship Model

**DOI:** 10.3390/ijms25042256

**Published:** 2024-02-13

**Authors:** Jiaming Zhang, Qinqin Liu, Haoxia Zhao, Guiyu Li, Yunpeng Yi, Ruofeng Shang

**Affiliations:** 1 Key Laboratory of New Animal Drug Project, Gansu Province/Key Laboratory of Veterinary Pharmaceutical Development, Ministry of Agriculture and Rural Affairs/Lanzhou Institute of Husbandry and Pharmaceutical Sciences of CAAS, Lanzhou 730050, Chinajyly0613@163.com (G.L.); 2 Shandong Provincial Animal and Poultry Green Health Products Creation Engineering Laboratory, Institute of Poultry Science, Shandong Academy of Agricultural Science, Jinan 250023, China

**Keywords:** pleuromutilin, QSAR, synthesis, antimicrobial activity, molecular docking

## Abstract

The quantitative structure–activity relationship (QSAR) is one of the most popular methods for the virtual screening of new drug leads and optimization. Herein, we collected a dataset of 955 MIC values of pleuromutilin derivatives to construct a 2D-QSAR model with an accuracy of 80% and a 3D-QSAR model with a non-cross-validated correlation coefficient (*r*^2^) of 0.9836 and a cross-validated correlation coefficient (*q*^2^) of 0.7986. Based on the obtained QSAR models, we designed and synthesized pleuromutilin compounds **1** and **2** with thiol-functionalized side chains. Compound **1** displayed the highest antimicrobial activity against both *Staphylococcus aureus* ATCC 29213 (*S. aureus*) and Methicillin-resistant *Staphylococcus aureus* (MRSA), with minimum inhibitory concentrations (MICs) < 0.0625 μg/mL. These experimental results confirmed that the 2D and 3D-QSAR models displayed a high accuracy of the prediction function for the discovery of lead compounds from pleuromutilin derivatives.

## 1. Introduction

The widespread use of antibiotics in humans has led to the rapid emergence of many drug-resistant strains. Almost all antibacterial agents have developed antibiotic resistance, which has posed a serious threat to global public health [1,2]. Methicillin-resistant *Staphylococcus aureus* (MRSA) is a top-priority pathogen listed by the Centers for Disease Control (CDC) [3]. It is estimated that each year, approximately 72,000 cases of serious invasive disease and 10,000 deaths are caused by MRSA in the US [4,5]. Therefore, the development of new antibacterial agents with novel structures and unique antimicrobial mechanisms is urgently needed to fight antimicrobial resistance [6,7].

**Figure 1:**
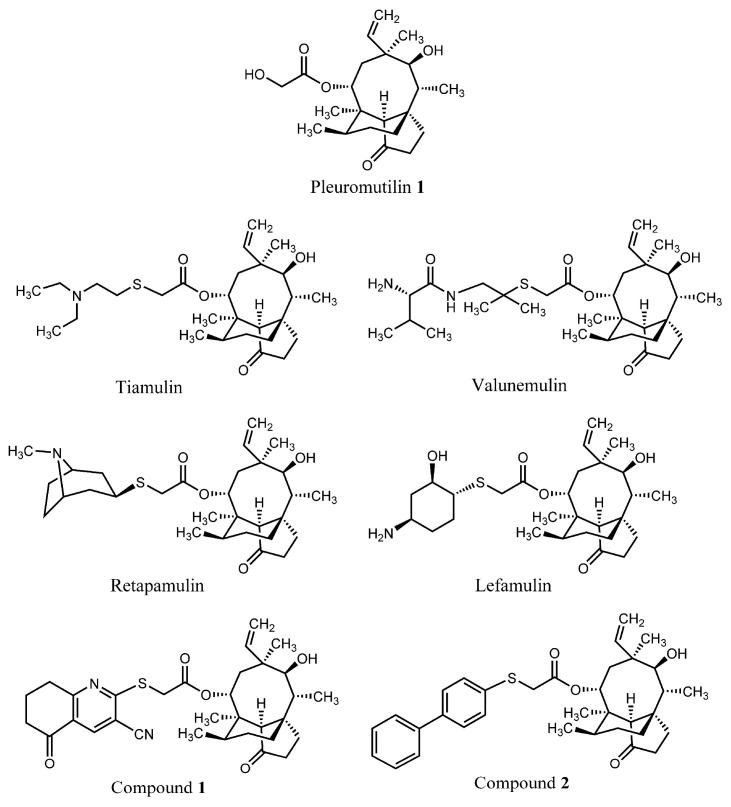
Structural formulas of pleuromutilin and derivatives.

Pleuromutilin (Figure 1) is a natural compound with a specific tricyclic diterpene structure produced by the *Pleurotus mutilus* [8]. After the discovery of pleuromutilin in 1951, tremendous efforts have been made to modify its scaffold to explore the structure–activity relationship (SAR) [9]. Modification of the pleuromutilin tricyclic ring does not improve their antibacterial activities significantly [10]. However, the presence of a thioether group at the side chain of pleuromutilin and a basic group enhances antibacterial activity dramatically [11]. Modifications of the side chain of pleuromutilin have led to tiamulin [12] and valnemulin [13] (Figure 1) for veterinary use. After that, retapamulin [14] and lefamulin [15] (Figure 1) were successfully developed for human use. Pleuromutilin compounds display good antibacterial activity against bacteria and mycoplasma by interacting with the 50S subunit of bacterial ribosomes to inhibit bacterial protein synthesis, but does not bind to mammalian ribosomes [16,17].

The quantitative structure–activity relationship (QSAR) has proved to be effective for scientific research in many disciplines, especially in the development of new generations of selective drugs [18]. To find new drug leads, efficient and stable procedures are required to screen chemical databases and virtual libraries for molecules with known activity. To this end, the QSAR model provides an effective way to explore and exploit the relationship between chemical structures and their biological effects for the development of new drug candidates [19,20,21]. The approach to building QSAR models can often be described as applying data analysis methods and statistical data, and the established model can accurately predict the biological activities or properties of a compound based on its structure. The 2D-QSAR is a method for quantitatively describing the relationship between the physicochemical and other measurable properties of a structure and its activity through a linear or non-linear model. This method has widely been used in many fields, such as medicine, pharmacy, and chemistry [22]. However, the 3D-QSAR introduces the three-dimensional structure of compound molecules to establish a link between the characteristic structure and its activity for obtaining better QSAR models [22].

In this study, the biological activity of target compounds **1** and **2** (Figure 1) was first designed and predicted based on the established 2D- and 3D-QSAR model. Subsequently, we synthesized and tested their antibacterial activities in vitro. The structure of pleuromutilin and the structure of the now-available pleuromutilin derivatives are shown in Figure 1.

## 2. Results and Discussion

### 2.1. QSAR Model Evaluation

The most simplistic predictive models of chemical properties rely on the similarity principle; especially, the compounds with similar structures have similar properties [21]. Therefore, we selected pleuromutilin derivatives whose side chains were modified but whose tricyclic diterpene structures were untouched, as the molecular target for QSAR predictions.

Ninety-six randomly selected untrained molecules were used for activity prediction with the 2D-QSAR model, and the prediction results were represented by the classification model labels (class-0, class-1, and class-2). The test set output showed the actual negative logarithmic value of minimum inhibitory concentrations (pMICs), as well as the test and prediction model labels, which were subsequently evaluated for comparison.

The model predicts four possible results: true positive (*TP*), false positive (*FP*), false negative (*FN*), and true negative (*TN*). Based on the prediction results, we used precision (precision or positive predictive value, PPV), recall (recall or sensitivity, true positive rate, TPR), *F*1-score (*F*1-value), and the confusion matrix to evaluate the model quality [23,24]. The four results of the model evaluation (Table 1) were calculated using the following formula:

$$Precision=\frac{TP}{TP+FP}$$

$$Recall=\frac{TP}{TP+FN}$$

$$F1-Score=\frac{2\times TP}{2\times TP+FP+FN}$$

**Figure 2:**
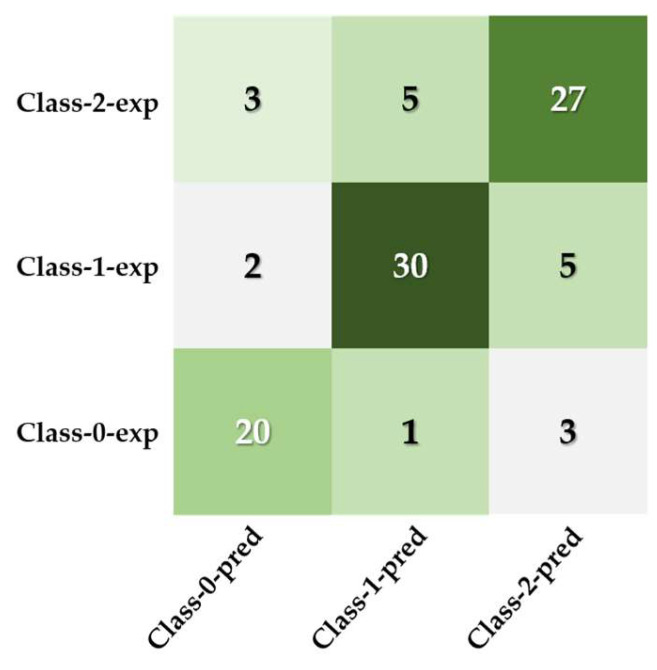
Confusion matrix diagram based on the prediction results. Class-0: pMIC in the range of ≥5.95. Class-1: pMIC in the range of 5.33–5.95. Class-2: pMICs <5.33. Figures in boxes indicate the number of the experimental and predicted molecules.

The confusion matrix, also known as the error matrix, is a standard format for representing accuracy evaluations, which is expressed in the form of a matrix with n rows and n columns. Based on the test result (class-exp.) and the prediction result (class-pred.), the confusion matrix was established as shown in Figure 2. The results showed that the higher the number of two-by-two data in the diagonal grid, the higher the number of experimental results that agree with the predicted results. Finally, we built a 2D-QSAR model with 80% accuracy.

**Figure 3:**
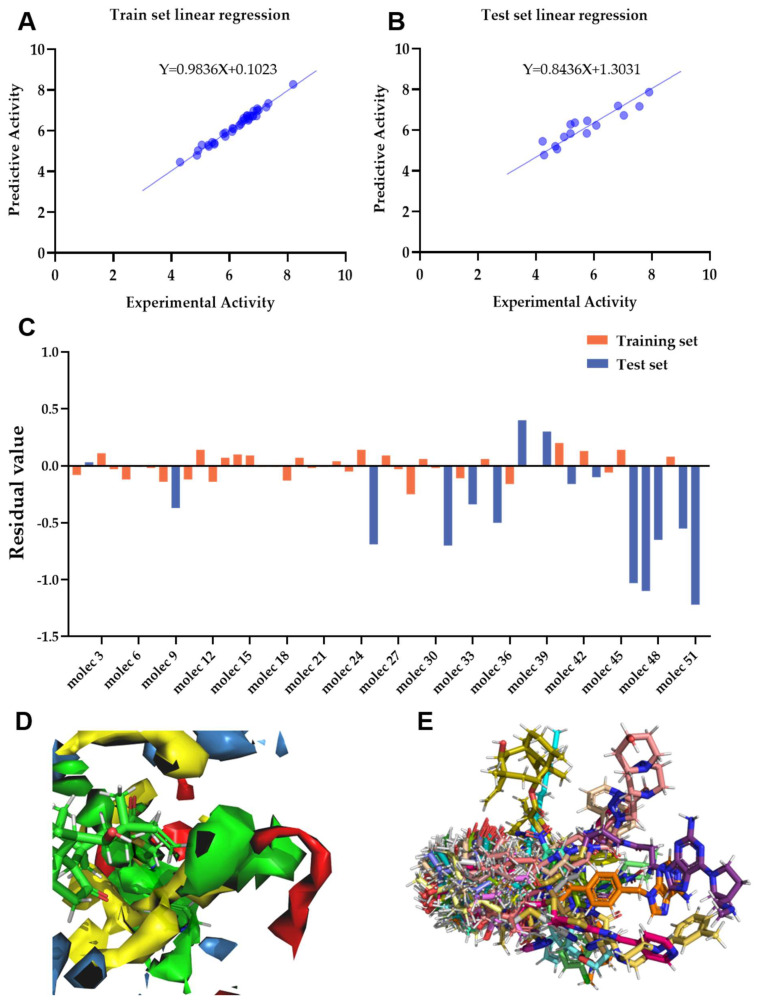
The evaluation results of 3D-QSAR model. (**A**) Training set linear regression; (**B**) test set linear regression; (**C**) residual value (experimental value–predictive value); (**D**) the field contour maps of the CoMFA models; (**E**) aligned molecules displayed by server.

The 3D-QSAR model mainly responds to the accuracy of the model by detecting the correlation coefficient *r*^2^ (the fitting ability of the response model) and the cross-validation coefficient *q*^2^ (the predictive ability of the response model). Generally, *r*^2^ > 0.8 and *q*^2^ > 0.6 indicate a better accuracy of the model [25]. The established model obtained better values of *r*^2^ (0.9836) and *q*^2^ (0.7986). The linear regression plots for the training and test sets are shown in Figure 3A,B, and the regression equation was built as Y = 0.9836X + 0.1023 and Y = 0.8436X + 1.3031. The differences between the experimental and the calculated values for MIC and both molecules in the training set and the test set are presented in Figure 3C. Force field models were visualized used PyMol software 3.8 version (DeLano Scientific LLC, San Carlos, CA, USA), and the template was set using the molecules with the highest antimicrobial activity in the training set (Figure 3D). The pleuromutilin ring and the thiazole ring were surrounded by a yellow profile, indicating that the bulky groups did not increase the antibacterial activity against MRSA. The blue profile was surrounded at 1-methyl-1H-1,2,3-triazole, indicating that it contained electronegative groups which were more beneficial to exhibiting anti-MRSA activity. The 3D view of the aligned molecules is also shown in Figure 3E. This information allows users to confirm the molecular alignment and validate the QSAR model.

### 2.2. Activity Prediction and Screening of New Compounds

Here, we designed and synthesized compounds **1** and **2** as examples to demonstrate the accuracy of our QSAR models. Compounds **1** and **2** contained a novel 7,8-dihydroquinolin-5(6H)-one and biphenyl side chain, respectively, which had not been reported by any study. The predicted results for compounds **1** and **2** are shown in Table 2. The MICs of both compounds predicted by the 2D-QSAR model were in the range of 0–0.5 μg/mL. In the 3D-QSAR model, compound **2** was predicted with higher activity than that of compound **2**.

### 2.3. Syntheses

**Scheme 1:**
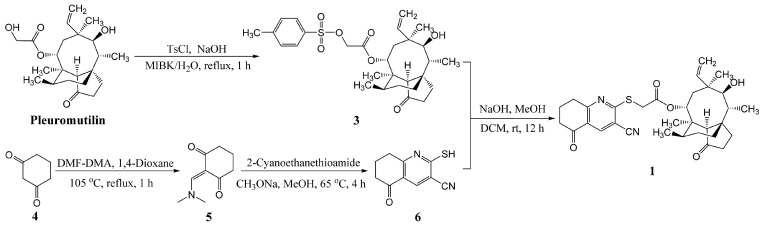
Synthetic route for compound **1**.

**Scheme 2:**
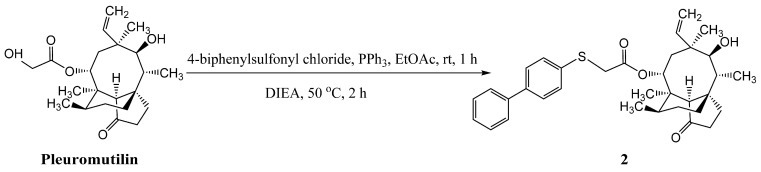
Synthetic route for compound **2**.

To explore the accuracy of the established 2D- and 3D-QSAR models, we performed concise syntheses of compounds **1** and **2**. To synthesize compound **1**, pleuromutilin was used as the starting material and converted into 22-O-tosylpleuromutilin **3**. Compound **6** was synthesized from the nucleophilic addition reaction of cyclohexanedione **4** with 1,1-dimethoxytrimethylamine, followed by cyclization with 2-cyanoethanethioamide. The electrophilic nature of **3** facilitated its nucleophilic substitution reaction with pyridine **6** to yield target compound **1** under the base condition (Scheme 1). Compound **2** was directly obtained by the reaction of pleuromutilin and 4-biphenylsulfonyl chloride with one step (Scheme 2).

**Figure 4:**
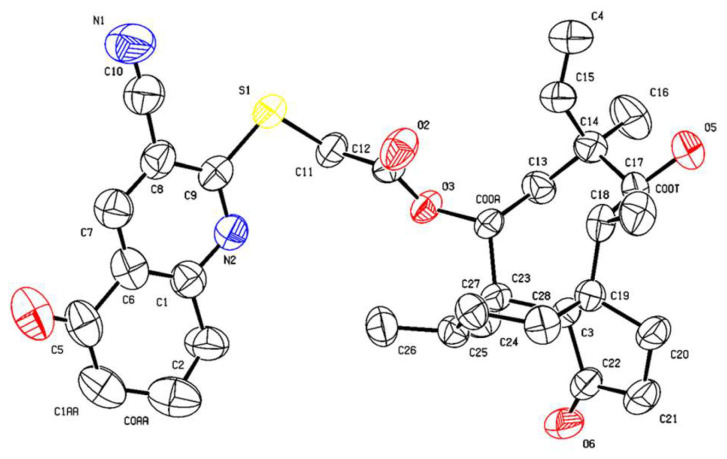
Crystal structure of compound **1** with ellipsoids set at 75% probability.

The structures of the synthesized compounds **1** and **2** were characterized via nuclear magnetic resonance spectroscopy (^1^H NMR and ^13^C NMR, the spectra are shown in Figures S1 and S2). In the ^1^H NMR spectrum of compound **1**, a pyridine proton and six methylene protons in 7,8-dihydroquinolin-5(6H)-one were observed in 8.49 and in the range of 2.39–3.42 ppm, respectively. Additionally, the ^13^C NMR spectra of compound **1** exhibited the characteristic signals of 168.71 and the range of 164.31–140.17 ppm for C=O and C=C(H) in 7,8-dihydroquinolin-5(6H)-one, respectively, which further confirmed the formation of the compounds. For compound **2**, nine typical benzene protons and five characteristic signals for C in biphenyl were observed in 7.48–7.26 ppm in the ^1^H NMR spectrum and in 130.70–126.93 ppm in the ^13^C NMR spectra, respectively. To further confirm the structure of compound **1**, a colorless and block-like crystal (CCDC: 2219819, the crystal data and structural refinement results are shown in Table S1) was obtained using the slow evaporation method at room temperature as highlighted in Figure 4.

### 2.4. Determination of MIC

The MICs of compounds **1** and **2** were determined as shown in Table 3. Compound **1** which bears a carbonitrile-substituted 7,8-dihydroquinolin-5(6H)-one side chain showed the highest inhibitory effect on MRSA and *S. aureus* ATCC 29213 with MIC < 0.0625 μg/mL for both strains, exhibiting a significantly higher activity than that of tiamulin. However, compound 2 with a biphenyl side chain failed to achieve better results, with MIC = 1 μg/mL for both strains. To our knowledge, no pleuromutilin derivative with a 7,8-dihydroquinolin-5(6H)-one side chain had been reported, which made it difficult to evaluate the anti-bacterial activities of compound **1** with structurally similar compounds. The MIC results of compound **1** were consistent with the predicted trends of the 2D-QSAR model, but the 3D-QSAR model was more accurate in the prediction of the activity of compound **2**.

### 2.5. Molecular Docking Study

**Figure 5:**
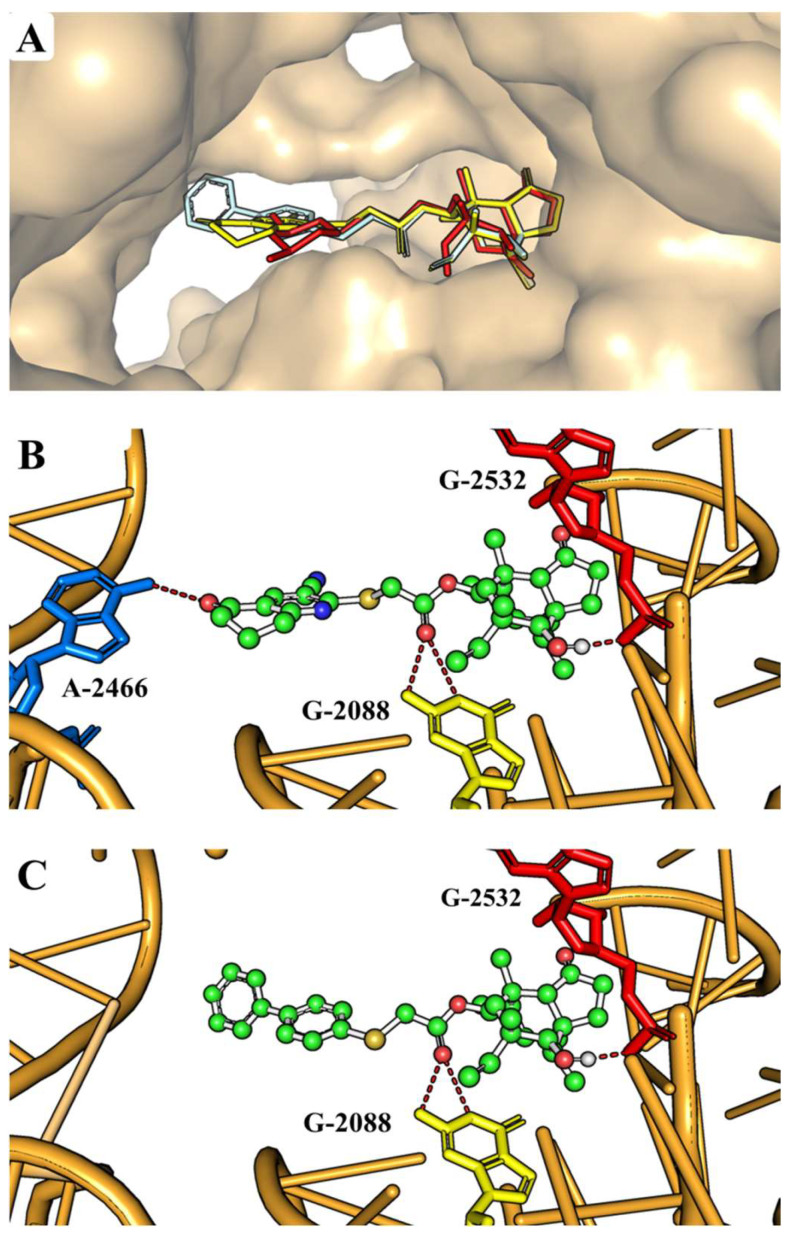
Superimposition of lefamulin (red) and compounds **1** (yellow) and **2** (cyan) docked to the binding pocket of ribosome (**A**). Binding modes of compounds **1** (**B**) and **2** (**C**) to 5H17. Important residues are drawn as sticks. Hydrogen bonds are shown as dashed red lines.

To investigate the possible binding pattern of compounds **1** and **2** to the peptidyl transferase center (PTC) domain of ribosome, molecular docking experiments were carried out using Autodock Vina [26]. Redocking the X-ray structure of lefamulin into 5HL7 [27] gave the best conformation of compounds **1** and **2,** which exhibited a similar binding pattern to that of lefamulin (Figure 5A).

The results showed that the affinity free energy of compound **1** and **2** was −9.69 and −8.13 kcal/mol, respectively. The hydrogen bonds formed between the hydroxyl groups (eight-membered ring) and C=O (ester) of the docked compounds and the residues of G-2532 and G-2088 were the key interactions (Figure 5B,C), which is similar to our previous report [28]. However, the carbonyl in the side chain of compound **1** formed an additional hydrogen bond with A-2466 (Figure 5B) when compared to compound **2**. Furthermore, no π–π or cation–π interaction between the residues and the pyridine or benzene rings of the docked compounds was observed.

### 2.6. ADMET Result

For further evaluation of PK properties, compounds **1** and **2** were evaluated for their absorption, distribution, metabolism, excretion, and toxicity (ADMET) in silico (Table 4). Distribution coefficient P resulted in a poor aqueous solubility but Caco-2 permeability was optimal. Compounds **1** and **2** were considered to be not only non-penetrating of the blood–brain barrier, but also non-inhibitors against CYP450 1A2 and 3A4. The low half-life (T_1/2_) indicated the fast metabolism of **1** and **2**. Furthermore, compounds **1** and **2** were considered low-toxicity by predicting the Ames result and LD_50_.

## 3. Materials and Methods

### 3.1. Build the 2D/3D-QSAR Model

The 2D-QSAR model was built based on a dataset consisting of 955 small molecules with MIC activity values against MRSA (SMILES names and their MIC values were found in Table S2) collected from available reports [22,23,29,30,31,32,33,34,35,36,37,38,39,40,41,42,43,44,45,46,47,48,49,50,51,52,53,54,55,56,57,58,59,60,61,62,63,64,65,66], with MIC ranging from 0 to 256 μg/mL, which were converted to pMIC to build the model. In this study, python 3.8, sklearn, and RDkit packages were used to complete the modeling work [67]. All the structures of selected compounds were converted to SMILES format by ChemDraw software, 17.0 version.

The random forest algorithm in integration science was applied to model the pMIC values of the target dataset for classification, and random forest classifier for decision tree modeling was used to solve the classification problem [68]. Before modeling, according to MIC values, we classified the dataset into three categories, including ≥5.95, 5.33–5.95, and <5.33, which denoted class-0, class-1, and class-2, respectively. The whole dataset was divided into the training set and test set which were randomly assigned by 8:1, the training set was used for model development, and the test set was used for model evaluation.

The building of the 3D-QSAR model requires selecting suitable small-molecule compounds and iteratively debugging the training and test sets. We used Cloud 3D-QSAR by integrating the functions of molecular structure generation, alignment, molecular interaction field (MIF) computing, and results analysis to provide a one-stop solution [69]. The most widely common CoMFA method was applied to our Cloud 3DQSAR server. A grid box step size of 1.0 Å was applied and the energy cutoff was set to 30 kcal/mol. For the MIFs, the steric field used a carbon atom probe, while the electrostatic field used a + 1 charged probe with negligible volume [69]. The partial least-squares (PLS) technique was applied to reduce the dimensionality in a regression context using orthogonal components. The server is free and provided at http://chemyang.ccnu.edu.cn/ccb/server/cloud3dQSAR/ (accessed on 6 December 2023). The relevant date about building the 3D-QSAR model can be found in Tables S3 and S4.

The SMILES and MIC values were uploaded to the website and divided into a training set (36 compounds) and a test set (15 compounds). After setting the iteration number to 300, the dataset was submitted, and the system debugged and built the model. The cloud system utilized hundreds of datasets to develop different CoMFA models. PLS and LOO CV were used to calculate *r*^2^ and *q*^2^ values. Based on the evaluation results, the cloud system displayed the top 50 models as the modeling results.

### 3.2. Chemistry

#### 3.2.1. Synthesis of Compound **3**

Pleuromutilin (379 mg, 1 mmol) in 8 mL DCM was added to Et_3_N (304 mg, 3 mmol). The mixture was stirred and tosyl chloride (210 mg, 1.1 mmol) was slowly added at room temperature. After reaction overnight, the solvent was extracted by 3× NaCl and 3× H_2_O. The combined organic layers were dried with magnesium sulfate and evaporated. Purification by flash chromatography provided compound **3** (274 mg, 72%) as a white solid.

#### 3.2.2. Synthesis of Compound **6**

Cyclohexane-1,3-dione (224 mg, 2 mmol) and 1,1-dimethoxy-N,N-dimethylmethanamine (238 mg, 2 mmol) in 1,4-dioxane were stirred for 2 h at room temperature. Then, the solvent was removed by evaporation to provide the crude product compound **5**. The crude product **5**, 2-cyanoethanethioamide (100 mg, 1 mmol), and sodium methoxide (540 mg, 1 mmol) in 10 mL MeOH were stirred for 3 h at 65 °C. The pH of reaction mixture was then adjusted to 5 with diluted hydrochloric acid, followed by filtering and rinsing with MeOH to obtain 0.165 g of orange substance compound **6**.

#### 3.2.3. Synthesis of Compound **1**

Compound **6** (102 mg, 0.5 mmol) in 5 mL MeOH was mixed with NaOH (22 mg) in 100 μL H_2_O and stirred at room temperature for 10 min. Compound **3** (226 mg, 0.5 mmol) in 10 mL DCM was added to the mixed solutions while stirring overnight. The reaction solution was extracted with DCM (3×), and the combined organic layers were dried with anhydrous sodium sulfate, filtered, and evaporated. Purification by flash chromatography provided compound **1** (200 mg, 75%) as a white solid. IR (KBr): 3447.76, 3214.31, 3115.02, 3018.76, 2919.85, 2851.31, 2249.23, 1687.49, 1594.19, 1495.55, 1436.11, 1376.41, 1222.27, 1166.37, 721.03 cm^−1^; ^1^H NMR (600 MHz, DMSO-d6) δ 8.49 (s, 1H), 6.12 (dd, *J* = 17.8, 11.2 Hz, 1H), 5.54 (d, *J* = 8.4 Hz, 1H), 5.05 (d, *J* = 17.8 Hz, 1H), 4.99 (d, *J* = 11.2 Hz, 1H), 4.53 (d, *J* = 6.2 Hz, 1H), 4.18 (d, *J* = 2.5 Hz, 2H), 3.42 (d, *J* = 6.0 Hz, 1H), 3.02 (d, *J* = 3.4 Hz, 2H), 2.65 (t, *J* = 6.6 Hz, 2H), 2.39 (s, 1H), 2.21–2.16 (m, 1H), 2.09 (d, *J* = 3.6 Hz, 1H), 2.07 (s, 1H), 2.06 (s, 1H), 2.04 (s, 1H), 2.02 (d, *J* = 7.3 Hz, 1H), 1.67–1.58 (m, 2H), 1.48 (d, *J* = 6.0 Hz, 1H), 1.39–1.34 (m, 1H), 1.33 (s, 3H), 1.27 (d, *J* = 6.6 Hz, 1H), 1.26–1.23 (m, 1H), 1.21 (d, *J* = 15.8 Hz, 1H), 1.03 (s, 3H), 0.99 (dd, *J* = 13.9, 4.4 Hz, 1H), 0.80 (d, *J* = 7.0 Hz, 3H), 0.63 (d, *J* = 7.1 Hz, 3H); ^13^C NMR (151 MHz, DMSO) δ 217.17, 196.40, 168.71, 166.86, 164.31, 141.17, 140.17, 124.58, 115.70, 115.33, 105.71, 72.98, 70.79, 57.58, 45.40, 44.47, 43.89, 42.00, 37.90, 36.79, 36.75, 34.46, 33.26, 32.84, 30.51, 28.86, 27.05, 24.89, 21.11, 16.71, 14.82, 11.93; HRMS (ES) calcd [M + H]^+^ for C_32_H_40_N_2_O_5_S 565.2658, found 565.2624.

#### 3.2.4. Synthesis of Compound **2**

Pleuromutilin (189 mg, 0.5 mmol), 4-biphenylsulfonyl chloride (190 mg, 0.75 mmol), and PPh_3_ (590 mg, 2.25 mmol) in 5 mL EtOAc were mixed and stirred at room temperature for 1 h, and then DIEA (129.24 mg, 1 mmol) was added and stirred at 50 °C for 2 h. Purification by flash chromatography provided compound **2** (106 mg, 56%) as a white solid. IR (KBr): 3460.38, 2954.85, 2928.12, 2882.22, 2864.10, 2360.74, 1728.87, 1480.18, 1456.69, 1276.46, 1149.80, 1116.20, 1016.65, 760.96 cm^−1^; ^1^H NMR (600 MHz, Chloroform-d) δ 7.48 (d, *J* = 7.1 Hz, 2H), 7.43 (d, J = 8.4 Hz, 2H), 7.37 (s, 2H), 7.36 (d, *J* = 6.5 Hz, 2H), 7.28 (d, *J* = 8.5 Hz, 1H), 6.34 (dd, *J* = 17.4, 11.0 Hz, 1H), 5.67 (d, *J* = 8.5 Hz, 1H), 5.23 (d, *J* = 11.0 Hz, 1H), 5.07 (d, *J* = 15.9 Hz, 1H), 3.53 (s, 2H), 3.25 (d, J = 6.5 Hz, 1H), 2.24 (t, *J* = 7.0 Hz, 1H), 2.17 (s, 1H), 2.12 (t, *J* = 9.4 Hz, 1H), 1.99 (s, 1H), 1.92 (dd, *J* = 16.1, 8.5 Hz, 1H), 1.68 (d, *J* = 14.5 Hz, 1H), 1.57 (d, *J* = 6.9 Hz, 2H), 1.46 (dd, *J* = 11.5, 2.6 Hz, 1H), 1.37 (s, 1H), 1.34 (s, 3H), 1.26 (s, 2H), 1.21 (s, 1H), 1.02 (s, 3H), 1.01 (s, 1H), 0.79 (d, *J* = 7.0 Hz, 3H), 0.62 (d, *J* = 7.0 Hz, 3H); ^13^C NMR (151 MHz, CDCl_3_) δ 217.05, 167.79, 140.27, 139.93, 138.91, 133.86, 130.70, 128.92, 127.67, 127.50, 126.93, 116.72, 74.62, 69.61, 58.19, 45.45, 44.77, 43.86, 41.78, 37.21, 36.77, 36.00, 34.46, 30.43, 26.84, 26.33, 24.84, 16.75, 15.45, 11.50; HRMS (ES) calcd [M + H]^+^ for C_34_H_42_O_4_S 547.2804, found 547.2835.

### 3.3. In Vitro Efficacy

The MICs of compounds **1** and **2**, as well as tiamulin used as a control drug, were determined to assess their antibacterial activity against MRSA and *S. aureus* ATCC 29213. The MIC values were recorded as the lowest drug concentration fully visible in the 96-well plate at 37 °C to inhibit bacterial growth. Compounds were diluted to working concentrations using ultrapure water containing no more than 5% dimethyl sulfoxide (DMSO). The experiments were performed according to the broth micro-dilution methods, using Mueller–Hinton broth medium (MHB) at half dilution and two replicate tests for each drug.

### 3.4. Molecular Modeling

We used smina [70] to perform the docking. In the protocol, ligands were constructed using chem3D with energy minimization. The X-ray crystal structure (PDB ID: 5HL7) [26] was used as ribosome reporter. After removing water, metal ions, and ligands, the combination pocket was positioned at x, y, and z of 17, −77.9, and −2.6 and the size was set to 20 × 20 × 20 Å. Lefamulin (ligand) was redocked and compared with original structure. The parameter of exhaustiveness arg and num_modes arg were set to 120 and 9, respectively. Interacting intermolecular hydrogen bonds were assessed using LigPlot 2.2 version and PyMol software 3.8 version.

### 3.5. ADMET Evaluation

The Lipinski rule describes the druggability of molecules, which bases pharmacokinetic drug properties such as absorption, distribution, metabolism, and excretion on specific molecular properties. In this study, physicochemical properties such as absorption, distribution, metabolism, excretion, and toxicity of compounds **1** and **2** were predicted using the ADMET online prediction tool. The tool is free and provided at https://admet.scbdd.com/home/index/ (accessed on 6 December 2023).

## 4. Conclusions

To screen the quality of pleuromutilin-like drugs more efficiently, the QSAR model was constructed in this study based on a large amount of experimental data. Random forest was used for establishing the 2D-QSAR model, which fit multiple categorical decision trees on various subsamples of the dataset and used averaging to improve prediction accuracy and control overfitting. The theoretical accuracy of the 2D-QSAR model established in this experiment was predicted as high as 80%. The 3D-QSAR model established in this study displayed good fitting and prediction abilities. We then designed and synthesized compounds **1** and **2** based on the 2D- and 3D-QSAR models. After determining their MIC, compound **1** displayed a higher antibacterial activity (MICs for both MRSA and *S. aureus* were <0.0625 μg/mL) than those of compound **2** and tiamulin, which was consistent with the predicted results of the 2D-QSAR model (0, i.e., the MIC of MRSA was in the range of ≥5.95). The prediction results of the 2D-QSAR model and 3D-QASR model for compound 1 were consistent with the experimental results; for compound 2, the 3D-QSAR model accurately predicted its activity compared to the 2D-QSAR model. This reflects the advantages of the 3D-QSAR: the 3D-QSAR introduces a three-dimensional stereo conformation, built by molecular superposition and iterative replacement of the training and test sets. Molecular docking revealed the improved activity of compound **1** attributed to the additional hydrogen bond with lower binding free energies (ΔGb) than those of compound **2**, further demonstrating the predicted accuracy of QSAR models. Based on our experimental data, the developed 2D- and 3D-QSAR models in this study helped to improve the efficiency of finding highly active pleuromutilin derivatives.

## Figures and Tables

**Table 1:** Evaluation of 2D-QSAR model accuracy.

	Precision	Recall	*F*1-Score	Support
Class-0	0.80	0.83	0.82	24
Class-1	0.83	0.81	0.82	37
Class-2	0.77	0.77	0.77	35

**Table 2:** Results of 2D- and 3D-QSAR model activity prediction.

Compound	2D-QSAR	3D-QSAR (pMIC)
**1**	Class-0	6.22
**2**	Class-0	5.83

**Table 3:** Compound antibacterial activity.

Compound	MIC (μg/mL)	
	MRSA ATCC 43300	*S. aureus* ATCC 29213
Tiamulin	0.0625	0.5
1	<0.0625	<0.0625
2	1	1

**Table 4:** ADMET prediction results of compounds **1** and **2**.

Comp.	Property	Absorption	Distribution	Metabolism		Excretion	Toxicity
	LogP	LogPapp	BBB	CYP450 1A2 Inhibitor	CYP450 3A4 Substrate	T1/2	Ames LD50
**1**	Poor aqueous solubility	Optimal	BBB−	Non-inhibitor	Non-substrate	Low	Negative Low-toxicity
**2**	Poor aqueous solubility	Optimal	BBB−	Non-inhibitor	Non-substrate	Low	Negative Low-toxicity

Note: LogP is classed as optimal (0 < LogP < 3), poor lipid bilayer permeability (<0) and poor aqueous solubility (>3); LogPapp (Caco-2 permeability) is predicted as optimal (≥−5.15 Log unit); BBB (blood–brain barrier) is classed as category 0 (BBB−) and 1 (BBB+); CYP1A2 inhibitor is classed as category 0 (non-inhibitor) and 1 (inhibitor); CYP450 1A2 substrate is classed as category 0 (non-substrate) and 1 (substrate); T_1/2_ (half-life) is classed as high (>8 h), moderate (3 h < T_1/2_ < 8 h), and low (<3 h); Ames (mutagenicity) is classed as category 0 (negative) and category 1 (positive); LD_50_ (acute toxicity) is classed as high-toxicity (1~50 mg/kg), medium-toxicity (51~500 mg/kg), and low-toxicity (501~5000 mg/kg).

## Data Availability

The original contributions presented in the study are included in the article and Supplementary Materials; further inquiries can be directed to the corresponding author.

## Supplementary Materials

The following supporting information can be downloaded at https://www.mdpi.com/article/10.3390/ijms25042256/s1.
