# Supplementary material for: Design and Synthesis of Pleuromutilin Derivatives as Antibacterial Agents Using Quantitative Structure–Activity Relationship Model
Source: Int J Mol Sci. 2024 Feb 13;25(4):2256. doi: 10.3390/ijms25042256 (PMC10888563; doi:10.3390/ijms25042256)
Supplement: Supplementary file 1 [file ijms-25-02256-s001.zip › ijms-2845222-supplementary.pdf]

Supporting Information

Design and Synthesis of Pleuromutilin Derivatives as Antibacterial Agents Using Quantitative Structure–Activity Relationship Model

Jiaming Zhang [1,2], Qinqin Liu [1], Haoxia Zhao [1], Guiyu Li [1], Yunpeng Yi [2,*] and Ruofeng Shang [1,*]

1 Key Laboratory of New Animal Drug Project, Gansu Province/Key Laboratory of Veterinary Pharmaceutical Development, Ministry of Agriculture and Rural Affairs/Lanzhou Institute of Husbandry and Pharmaceutical Sciences of CAAS, Lanzhou 730050, China; jiamingzhang1998@foxmail.com (J.Z.); l18736127157@163.com (Q.L.); haoxiaz0112@163.com (H.Z.); jyly0613@163.com (G.L.)

2 Shandong Provincial Animal and Poultry Green Health Products Creation Engineering Laboratory, Institute of Poultry Science, Shandong Academy of Agricultural Science, Jinan 250023, China

* Correspondence: yiyunpeng@saas.ac.cn (Y.Y.); shangruofeng@caas.cn (R.S.); Tel.: +86-531-66655359 (Y.Y.); +86-931-2115253 (R.S.); Fax: +86-531-66655350 (Y.Y.); +86-931-2115951 (R.S.)

Figure S1. IR, ^{1}H NMR and ^{13}C NMR spectrum of compound **1**.

Figure S2. IR, ^{1}H NMR and ^{13}C NMR spectrum of compound **2**.

Table S1. Summary of crystal data and structural refinement results for compound **1**.

Table S2. Summary of 955 small molecules with MIC (pMIC) activity values against MRSA.

Table S3. Summary of 51 compounds for 3D-QSAR model construction.

Table S4 Summary of experimental versus predictive activity(3D-QSAR).

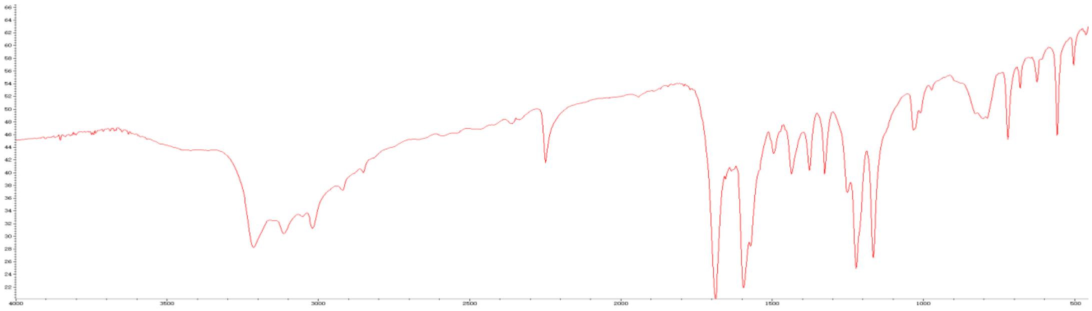

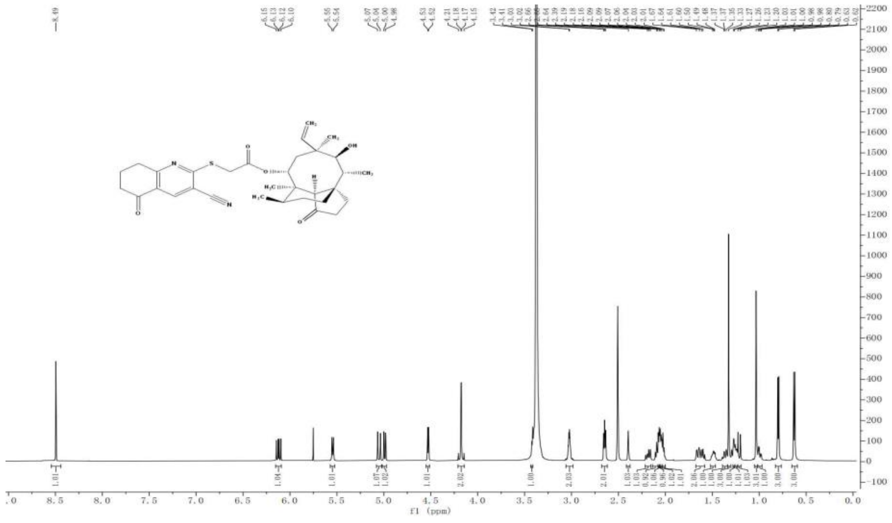

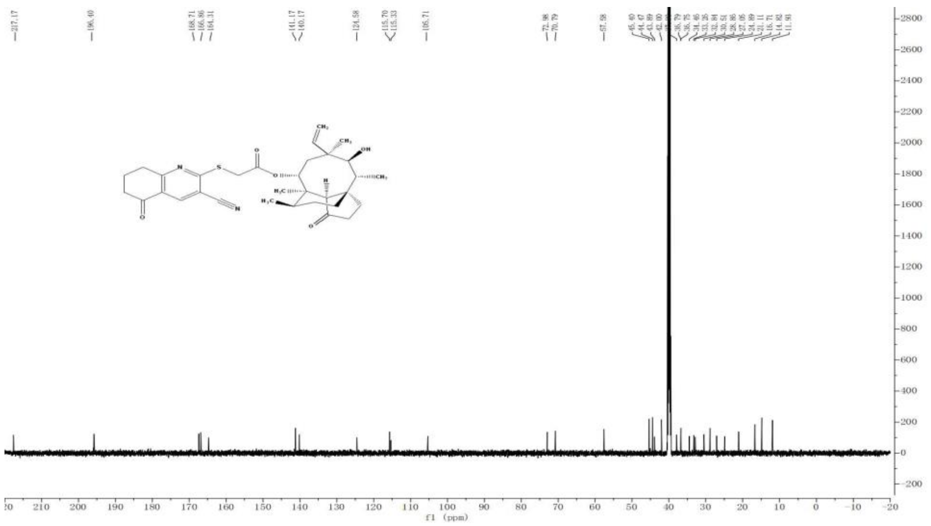

Figure S1. IR, ^{1}H and ^{13}C NMR spectrum of the compound **1**.

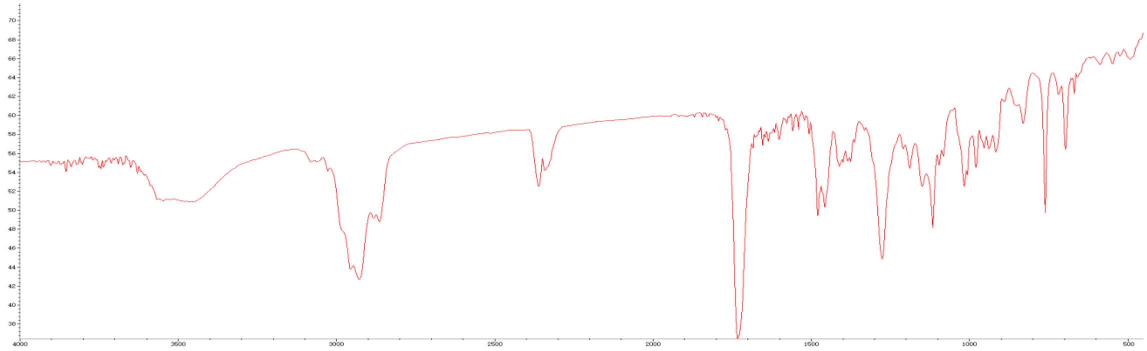

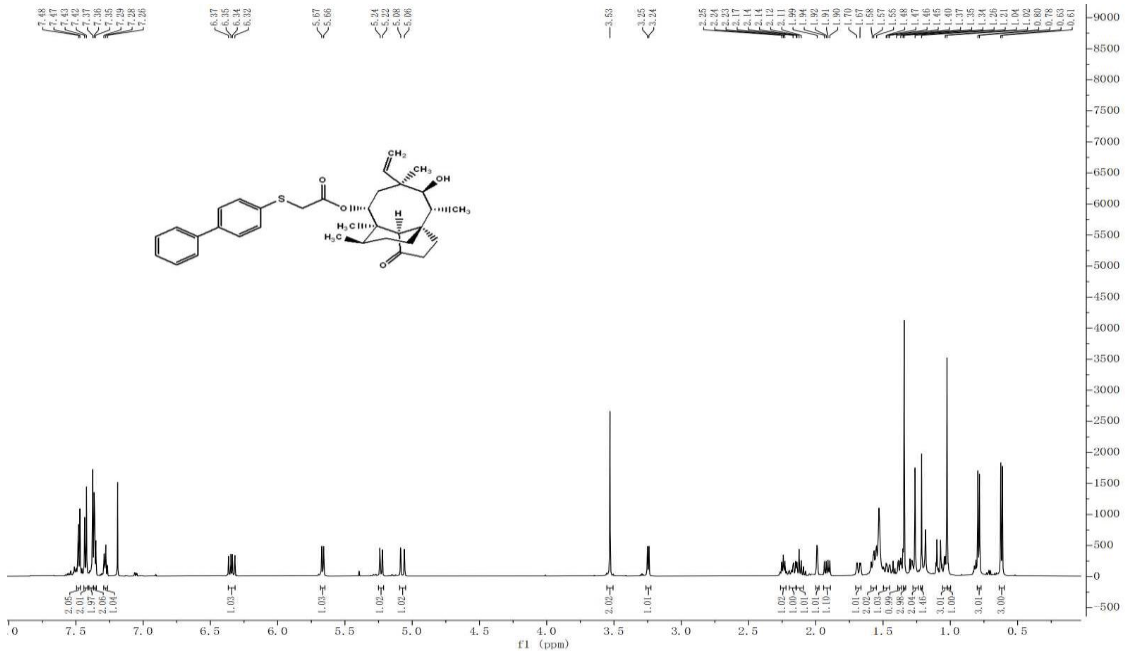

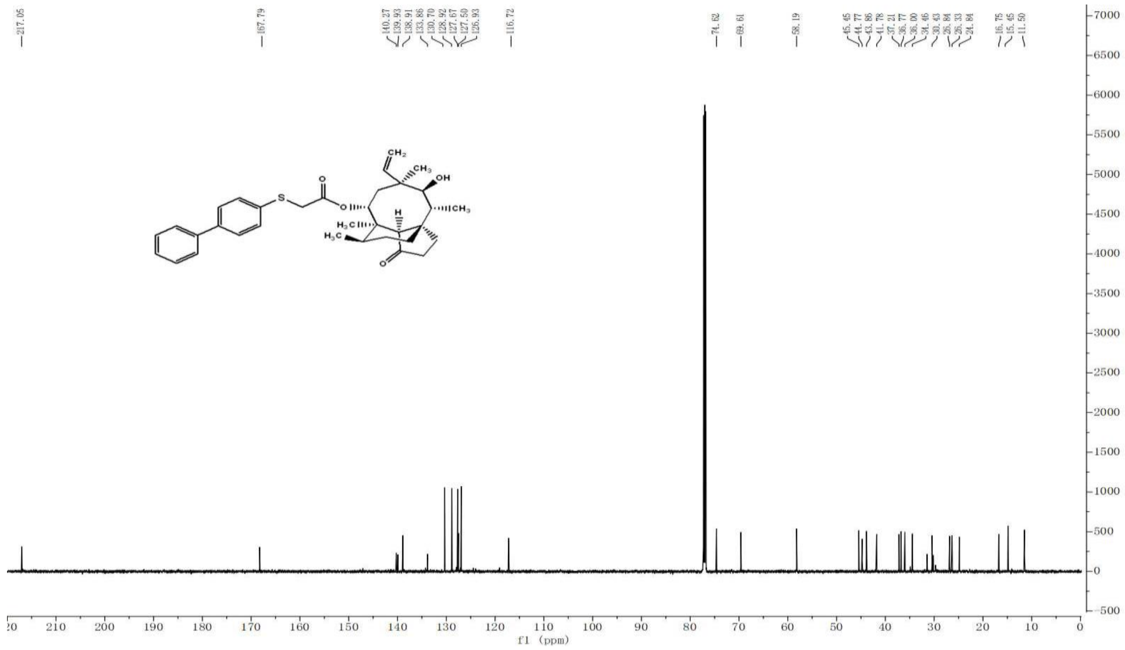

Figure S2. IR, ¹H and ¹³C NMR spectrum of the compound **2**.

Table S1. Summary of crystal data and structural refinement results for compound **1**.

Temperature/K	296
Crystal system	Monoclinic
Space group	*P*2₁
a/Å	9.945 (3)
b/Å	9.315 (2)
c/Å	16.462 (4)
β	103.968 (5)°
Volume/Å³	1480.0 (7)
Z	1
F(000)	524.5332
μ/mm⁻¹	0.15
Radiation	MoKα (λ = 0.71073)
D_x(g/cm³)	1.177
μ/mm⁻¹	0.15
R_{int} 0.027	0.027
(sin θ/λ)$_{max}$ (Å⁻¹)	0.668
R[F² > 2σ(F²)], wR(F²), S	0.079, 0.253, 1.04
No. of reflections	6769
No. of parameters	361
No. of restraints	1
Δρ$_{max}$, Δρ$_{min}$ (e Å⁻³)	0.46, −0.29
Absolute structure parameter	−0.06 (12)
R[F² > 2σ(F²)]	0.079

Table S2. Summary of 955 small molecules with MIC activity values against MRSA.

SMILES	pMIC
O=C(CC1)[C@]2([H])[C@@]31[C@@H](C)[C@H](O)[C@](C)(C=C)C[C@@H](OC(CSCC4=CSC(NC(C5=CC=NC=C5)=O)=N4)=O)[C@]2(C)[C@H](C)CC3	8.20
O=C(CC1)[C@]2([H])[C@@]31[C@@H](C)[C@H](O)[C@](C)(C=C)C[C@@H](OC(CSCC4=CSC(NC(C5=CC=CC(N)=C5)=O)=N4)=O)[C@]2(C)[C@H](C)CC3	7.90
O=C(CC1)[C@]2([H])[C@@]31[C@@H](C)[C@H](O)[C@](C)(C=C)C[C@@H](OC(CSCC4=CSC(NC(C5=CC=C(N)C=C5)=O)=N4)=O)[C@]2(C)[C@H](C)CC3	7.90
O=C(CC1)[C@]2([H])[C@@]31[C@@H](C)[C@H](O)[C@](C)(C=C)C[C@@H](OC(CSC4=CC(C(NC5=CC=C(S(=O)(NC6=CC=CC=N6)=O)C=C5)=O)=CC=C4)=O)[C@]2(C)[C@H](C)CC3	7.67
O=C(CC1)[C@]2([H])[C@@]31[C@@H](C)[C@H](O)[C@](C)(C=C)C[C@@H](OC(CN4CCN(C5=CC=C([N+]([O-])=O)C=C5)CC4)=O)[C@]2(C)[C@H](C)CC3	7.58
O=C(CC1)[C@]2([H])[C@@]31[C@@H](C)[C@H](O)[C@](C)(C=C)C[C@@H](OC(CN4CCN(C5=CC=CC=C5Cl)CC4)=O)[C@]2(C)[C@H](C)CC3	7.57
O=C(CC1)[C@]2([H])[C@@]31[C@@H](C)[C@H](O)[C@](C)(C=C)C[C@@H](OC(CN4CCN(C5=CC=C(Cl)C=C5)CC4)=O)[C@]2(C)[C@H](C)CC3	7.57
O=C(CC1)[C@]2([H])[C@@]31[C@@H](C)[C@H](O)[C@](C)(C=C)C[C@@H](OC(CSC4=C(N)C=CC=C4)=O)[C@]2(C)[C@H](C)CC3	7.49
O=C(CC1)[C@]2([H])[C@@]31[C@@H](C)[C@H](O)[C@](C)(C=C)C[C@@H](OC(CN4N=NC(C5=CC=C(CN6C7=NC=NC(N)=C7N=C6)C=C5)=C4)=O)[C@]2(C)[C@H](C)CC3	7.34
O=C(CC1)[C@]2([H])[C@@]31[C@@H](C)[C@H](O)[C@](C)(C=C)C[C@@H](OC(CSC4=CC(C(NC5=CC=C(S(=O)(N)=O)C=C5)=O)=CC=C4)=O)[C@]2(C)[C@H](C)CC3	7.32
O=C(CC1)[C@]2([H])[C@@]31[C@@H](C)[C@H](O)[C@](C)(C=C)C[C@@H](OC(CN4CCN(C5=CC=CC(Cl)=C5)CC4)=O)[C@]2(C)[C@H](C)CC3	7.27
O=C(CC1)[C@]2([H])[C@@]31[C@@H](C)[C@H](O)[C@](C)(C=C)C[C@@H](OC(CNC4=CC=CC(F)=C4)=O)[C@]2(C)[C@H](C)CC3	7.20
O=C(CC1)[C@]2([H])[C@@]31[C@@H](C)[C@H](O)[C@](C)(C=C)C[C@@H](OC(CSC4CCN(C(CCN5C=NC6=C5N=C(N)N=C6)=O)CC4)=O)[C@]2(C)[C@H](C)CC3	7.13
O=C(CC1)[C@]2([H])[C@@]31[C@@H](C)[C@H](O)[C@](C)(C=C)C[C@@H](OC(CSC4CCN(C(CCN5C=NC6=C5N=CN=C6)=O)CC4)=O)[C@]2(C)[C@H](C)CC3	7.12
O=C(CC1)[C@]2([H])[C@@]31[C@@H](C)[C@H](O)[C@](C)(C=C)C[C@@H](OC(CSC4CCN(C(CCN5C=NC6=C5N=CN=C6N7CCNCC7)=O)CC4)=O)[C@]2(C)[C@H](C)CC3	7.07
CC1C23C(C(CC3)=O)C(C(CC(C=C)(C)C1O)OC(CN4CCN(C(CN5CCN(C6=C([N+]([O-])=O)C=CC=C6)CC5)=O)CC4)=O)(C(CC2)C)C	7.04
O=C(CC1)[C@]2([H])[C@@]31[C@@H](C)[C@H](O)[C@](C)(C=C)C[C@@H](OC(CSC4=CC(CO)=CN=C4)=O)[C@]2(C)[C@H](C)CC3	7.03
O=C(CC1)[C@]2([H])[C@@]31[C@@H](C)[C@H](O)[C@](C)(C=C)C[C@@H](OC(CSC4=CC(CO)=NC=C4)=O)[C@]2(C)[C@H](C)CC3	7.03
O=C(CC1)[C@]2([H])[C@@]31[C@@H](C)[C@H](O)[C@](C)(C=C)C[C@@H](OC(CN4N=NC(C5=CC=CC(N6C7=NC=NC(N)=C7N=C6)=C5)=C4)=O)[C@]2(C)[C@H](C)CC3	7.03
O=C(CC1)[C@]2([H])[C@@]31[C@@H](C)[C@H](O)[C@](C)(C=C)C[C@@H](OC(CN4N=NC(C5=CC=CC(N6C=NC7=C6N=CN=C7N)=C5)=C4)=O)[C@]2(C)[C@H](C)CC3	7.03
O=C(CC1)[C@]2([H])[C@@]31[C@@H](C)[C@H](O)[C@](C)(C=C)C[C@@H](OC(CSCC4=CSC(NC(C5=CC=C([N+]([O-])=O)C=C5)=O)=N4)=O)[C@]2(C)[C@H](C)CC3	7.02
O=C(CC1)[C@]2([H])[C@@]31[C@@H](C)[C@H](O)[C@](C)(C=C)C[C@@H](OC(CSC4=CC(N)=CN=C4)=O)[C@]2(C)[C@H](C)CC3	7.02
O=C1CC[C@]2(CC[C@H]3C)[C@]1([H])[C@]3(C)[C@H](OC(CSC4=NC(NC=C5)=C5C(NC(CN(CC)CC)=O)=N4)=O)C[C@@](C)(C=C)[C@@H](O)[C@@H]2C	7.01
O=C(CC1)[C@]2([H])[C@@]31[C@@H](C)[C@H](O)[C@](C)(C=C)C[C@@H](OC(CSC4=C(NC(CN5CCOCC5)=O)C=CC=C4)=O)[C@]2(C)[C@H](C)CC3	7.01
O=C1CC[C@]2(CC[C@H]3C)[C@]1([H])[C@]3(C)[C@H](OC(CSC4=NC(NC=C5)=C5C(NC(CN6CCCC6)=O)=N4)=O)C[C@@](C)(C=C)[C@@H](O)[C@@H]2C	7.01
O=C(CC1)[C@]2([H])[C@@]31[C@@H](C)[C@H](O)[C@](C)(C=C)C[C@@H](OC(CSC4=NC=CC=C4)=O)[C@]2(C)[C@H](C)CC3	7.00
O=C(CC1)[C@]2([H])[C@@]31[C@@H](C)[C@H](O)[C@](C)(C=C)C[C@@H](OC(CSC4=CC(NC(CN5CCC(O)CC5)=O)=CN=C4)=O)[C@]2(C)[C@H](C)CC3	7.00
O=C(CC1)[C@]2([H])[C@@]31[C@@H](C)[C@H](O)[C@](C)(C=C)C[C@@H](OC(CN4CCN(C(/C=C/C5=CC=C([N+]([O-])=O)C=C5)=O)CC4)=O)[C@]2(C)[C@H](C)CC3	7.00
O=C(CC1)[C@]2([H])[C@@]31[C@@H](C)[C@H](O)[C@](C)(C=C)C[C@@H](OC(CS(SC4=NC5=C(C=CC(OC)=C5)N4)=O)=O)[C@]2(C)[C@H](C)CC3	6.99
O=C(CC1)[C@]2([H])[C@@]31[C@@H](C)[C@H](O)[C@](C)(C=C)C[C@@H](OC(CSCC4=CSC(NC(C5=CC=CC=C5)=O)=N4)=O)[C@]2(C)[C@H](C)CC3	6.99
O=C(CC1)[C@]2([H])[C@@]31[C@@H](C)[C@H](O)[C@](C)(C=C)C[C@@H](OC(CS(SC4=NC(OC)=CC(OC)=N4)=O)=O)[C@]2(C)[C@H](C)CC3	6.99
O=C(CC1)[C@]2([H])[C@@]31[C@@H](C)[C@H](O)[C@](C)(C=C)C[C@@H](OC(CS(SC4=NC5=C(C=CC=C5)S4)=O)=O)[C@]2(C)[C@H](C)CC3	6.98
O=C(CC1)[C@]2([H])[C@@]31[C@@H](C)[C@H](O)[C@](C)(C=C)C[C@@H](OC(CSCC4=CSC(NC(C5=CC=CO5)=O)=N4)=O)[C@]2(C)[C@H](C)CC3	6.98
O=C(CC1)[C@]2([H])[C@@]31[C@@H](C)[C@H](O)[C@](C)(C=C)C[C@@H](OC(CSC4=CC(NC([C@@H](N)CC(C)C)=O)=CN=C4)=O)[C@]2(C)[C@H](C)CC3	6.98
O=C(CC1)[C@]2([H])[C@@]31[C@@H](C)[C@H](O)[C@](C)(C=C)C[C@@H](OC(CN4CCN(C(/C=C/C5=CC=C(N)C=C5)=O)CC4)=O)[C@]2(C)[C@H](C)CC3	6.98
O=C(CC1)[C@]2([H])[C@@]31[C@@H](C)[C@H](O)[C@](C)(C=C)C[C@@H](OC(CS(SC4=NC(N)=CC(N)=N4)=O)=O)[C@]2(C)[C@H](C)CC3	6.96
O=C(CC1)[C@]2([H])[C@@]31[C@@H](C)[C@H](O)[C@](C)(C=C)C[C@@H](OC(CSC4=NN=C(C5=CC=CC(Cl)=C5)N4)=O)[C@]2(C)[C@H](C)CC3	6.96
O=C(CC1)[C@]2([H])[C@@]31[C@@H](C)[C@H](O)[C@](C)(C=C)C[C@@H](OC(CSCCNC(C4=CC=C(OC)C=C4)=O)=O)[C@]2(C)[C@H](C)CC3	6.96
O=C(CC1)[C@]2([H])[C@@]31[C@@H](C)[C@H](O)[C@](C)(C=C)C[C@@H](OC(CSC4=NN=C(C5=CC=CC=C5OC)N4)=O)[C@]2(C)[C@H](C)CC3	6.96
O=C(CC1)[C@]2([H])[C@@]31[C@@H](C)[C@H](O)[C@](C)(C=C)C[C@@H](OC(CN4CCN(C5=CC=CC=C5[N+]([O-])=O)CC4)=O)[C@]2(C)[C@H](C)CC3	6.96
O=C(CC1)[C@]2([H])[C@@]31[C@@H](C)[C@H](O)[C@](C)(C=C)C[C@@H](OC(CN4CCN(C5=CC=CC([N+]([O-])=O)=C5)CC4)=O)[C@]2(C)[C@H](C)CC3	6.96
O=C(CC1)[C@]2([H])[C@@]31[C@@H](C)[C@H](O)[C@](C)(C=C)C[C@@H](OC(CS(SC4=NN=C(N)S4)=O)=O)[C@]2(C)[C@H](C)CC3	6.96
O=C(CC1)[C@]2([H])[C@@]31[C@@H](C)[C@H](O)[C@](C)(C=C)C[C@@H](OC(CS(SC4=NN=C(C)S4)=O)=O)[C@]2(C)[C@H](C)CC3	6.95
O=C(CC1)[C@]2([H])[C@@]31[C@@H](C)[C@H](O)[C@](C)(C=C)C[C@@H](OC(CSC4=NN=C(C5=CC=CC=C5F)N4)=O)[C@]2(C)[C@H](C)CC3	6.95
O=C(CC1)[C@]2([H])[C@@]31[C@@H](C)[C@H](O)[C@](C)(C=C)C[C@@H](OC(CSC4=NN=C(C5=CC=CC=C5C)N4)=O)[C@]2(C)[C@H](C)CC3	6.95
O=C(CC1)[C@]2([H])[C@@]31[C@@H](C)[C@H](O)[C@](C)(C=C)C[C@@H](OC(CS(SC4=NC=CC=N4)=O)=O)[C@]2(C)[C@H](C)CC3	6.94
O=C(CC1)[C@]2([H])[C@@]31[C@@H](C)[C@H](O)[C@](C)(C=C)C[C@@H](OC(CS(SC4=CC=NC=C4)=O)=O)[C@]2(C)[C@H](C)CC3	6.94
O=C(CC1)[C@]2([H])[C@@]31[C@@H](C)[C@H](O)[C@](C)(C=C)C[C@@H](OC(CS(SC4=NC=CC=C4)=O)=O)[C@]2(C)[C@H](C)CC3	6.94
O=C(CC1)[C@]2([H])[C@@]31[C@@H](C)[C@H](O)[C@](C)(C=C)C[C@@H](OC(CSC4=CC(NC(CN)=O)=CN=C4)=O)[C@]2(C)[C@H](C)CC3	6.94
O=C(CC1)[C@]2([H])[C@@]31[C@@H](C)[C@H](O)[C@](C)(C=C)C[C@@H](OC(CN4CCN(C5=CC=CC=C5C)CC4)=O)[C@]2(C)[C@H](C)CC3	6.93
O=C(CC1)[C@]2([H])[C@@]31[C@@H](C)[C@H](O)[C@](C)(C=C)C[C@@H](OC(CNC4=CC=C(F)C=C4)=O)[C@]2(C)[C@H](C)CC3	6.90
O=C(CC1)[C@]2([H])[C@@]31[C@@H](C)[C@H](O)[C@](C)(C=C)C[C@@H](OC(CSC4=CN=CC=C4)=O)[C@]2(C)[C@H](C)CC3	6.88
O=C(CC1)[C@]2([H])[C@@]31[C@@H](C)[C@H](O)[C@](C)(C=C)C[C@@H](OC(CSC4CCN(C(CCN5C=NC6=C5N=C(N)N=C6N(C)C)=O)CC4)=O)[C@]2(C)[C@H](C)CC3	6.84
O=C(CC1)[C@]2([H])[C@@]31[C@@H](C)[C@H](O)[C@](C)(C=C)C[C@@H](OC(CSC4=CC(NC(CN5CC(CO)CCC5)=O)=CN=C4)=O)[C@]2(C)[C@H](C)CC3	6.83
O=C(CC1)[C@]2([H])[C@@]31[C@@H](C)[C@H](O)[C@](C)(C=C)C[C@@H](OC(CSC4CCN(C(CCN5C=NC6=C5N=C(N)N=C6N)=O)CC4)=O)[C@]2(C)[C@H](C)CC3	6.83
O=C(CC1)[C@]2([H])[C@@]31[C@@H](C)[C@H](O)[C@](C)(C=C)C[C@@H](OC(CSC4=CC(NC(CN5CC(O)CCC5)=O)=CN=C4)=O)[C@]2(C)[C@H](C)CC3	6.82
O=C(CC1)[C@]2([H])[C@@]31[C@@H](C)[C@H](O)[C@](C)(C=C)C[C@@H](OC(CSC4CCN(C(CCN5C=NC6=C5C=NC=N6)=O)CC4)=O)[C@]2(C)[C@H](C)CC3	6.81

O=C(CC1)[C@]2([H])[C@@]31[C@@H](C)[C@H](O)[C@](C)(C=C)C[C@@H](OC(CSC4=C(NC(CN5N=NC(CN6CCN(C7=CC=CC([N+]([O-])=O)=C7)CC6)=C5)=O)C=CC=C4)=O)[C@]2(C)[C@H](C)CC3	6.81
O=C(CC1)[C@]2([H])[C@@]31[C@@H](C)[C@H](O)[C@](C)(C=C)C[C@@H](OC(CSC4=C(NC(CN5N=NC(CN6CCN(C7=CC=C([N+]([O-])=O)C=C7)CC6)=C5)=O)C=CC=C4)=O)[C@]2(C)[C@H](C)CC3	6.81
O=C(CC1)[C@]2([H])[C@@]31[C@@H](C)[C@H](O)[C@](C)(C=C)C[C@@H](OC(CSC4CCN(C(CCN5C=NC6=C5N=C(N)N=C6N7CCN(C)CC7)=O)CC4)=O)[C@]2(C)[C@H](C)CC3	6.79
O=C(CC1)[C@]2([H])[C@@]31[C@@H](C)[C@H](O)[C@](C)(C=C)C[C@@H](OC(CSC4CCN(C(CCN5C=NC6=C5N=CN=C6N7CC(N)CCC7)=O)CC4)=O)[C@]2(C)[C@H](C)CC3	6.78
O=C(CC1)[C@]2([H])[C@@]31[C@@H](C)[C@H](O)[C@](C)(C=C)C[C@@H](OC(CSC4CCN(C(CCN5C=NC6=C5N=CN=C6N7CCC(C7)N)=O)CC4)=O)[C@]2(C)[C@H](C)CC3	6.77
O=C(CC1)[C@]2([H])[C@@]31[C@@H](C)[C@H](O)[C@](C)(C=C)C[C@@H](OC(CSC4CCN(C(CCN5C=NC6=C5N=CN=C6N7CC(N)CC7)=O)CC4)=O)[C@]2(C)[C@H](C)CC3	6.77
O=C(CC1)[C@]2([H])[C@@]31[C@@H](C)[C@H](O)[C@](C)(C=C)C[C@@H](OC(CSCC4=CC(C(O)=C(CO)O4)=O)=O)[C@]2(C)[C@H](C)CC3	6.77
O=C(CC1)[C@]2([H])[C@@]31[C@@H](C)[C@H](O)[C@](C)(C=C)C[C@@H](OC(CSC4=C(NC(CN5CCN(C6=CC=CC(O)=C6)CC5)=O)C=CC=C4)=O)[C@]2(C)[C@H](C)CC3	6.75
CC1C23C(C(CC3)=O)C(C(CC(C=C)(C)C1O)OC(CN4CCN(C(CN5CCN(C6=CC=C([N+]([O-])=O)C=C6)CC5)=O)CC4)=O)(C(CC2)C)C	6.74
CC1C23C(C(CC3)=O)C(C(CC(C=C)(C)C1O)OC(CN4CCN(C(CN5CCN(C6=CC=C(Cl)C=C6)CC5)=O)CC4)=O)(C(CC2)C)C	6.74
O=C(CC1)[C@]2([H])[C@@]31[C@@H](C)[C@H](O)[C@](C)(C=C)C[C@@H](OC(N(CC4)C(C5=C4C=CC(N)=C5)=O)=O)[C@]2(C)[C@H](C)CC3	6.73
O=C(CC1)[C@]2([H])[C@@]31[C@@H](C)[C@H](O)[C@](C)(C=C)C[C@@H](OC(CSCC4=CSC(NC(CN5CCN(CCO)CC5)=O)=N4)=O)[C@]2(C)[C@H](C)CC3	6.73
O=C(CC1)[C@]2([H])[C@@]31[C@@H](C)[C@H](O)[C@](C)(C=C)C[C@@H](OC(CSCC4=CSC(NC(CN5CCN(CCO)CC5)=O)=N4)=O)[C@]2(C)[C@H](C)CC3	6.73
O=C(CC1)[C@]2([H])[C@@]31[C@@H](C)[C@H](O)[C@](C)(C=C)C[C@@H](OC(CN4CCN(C(/C=C/C5=C(F)C=CC(F)=C5)=O)CC4)=O)[C@]2(C)[C@H](C)CC3	6.73
O=C(CC1)[C@]2([H])[C@@]31[C@@H](C)[C@H](O)[C@](C)(C=C)C[C@@H](OC(CSC4=CC(CO)=CC=C4)=O)[C@]2(C)[C@H](C)CC3	6.73
O=C(CC1)[C@]2([H])[C@@]31[C@@H](C)[C@H](O)[C@](C)(C=C)C[C@@H](OC(N4C(C(C=CC=C5N)=C5C4)=O)=O)[C@]2(C)[C@H](C)CC3	6.72
O=C(CC1)[C@]2([H])[C@@]31[C@@H](C)[C@H](O)[C@](C)(C=C)C[C@@H](OC(N4C(C(C=C(N)C=C5)=C5C4)=O)=O)[C@]2(C)[C@H](C)CC3	6.72
O=C1CC[C@]2(CC[C@H]3C)[C@]1([H])[C@]3(C)[C@H](OC(CSC4=NC(N)=CC(NC(CN5CCC[C@H](CO)C5)=O)=N4)=O)C[C@@](C)(C=C)[C@@H](O)[C@@H]2C	6.72
O=C(CC1)[C@]2([H])[C@@]31[C@@H](C)[C@H](O)[C@](C)(C=C)C[C@@H](OC(CSC4=NC(NC(CN5CCC[C@H](CO)C5)=O)=CC(N)=N4)=O)[C@]2(C)[C@H](C)CC3	6.72
O=C1CC[C@]2(CC[C@H]3C)[C@]1([H])[C@]3(C)[C@H](OC(CSC4=NC(NC=C5)=C5C(NC(CN6CCCCC6)=O)=N4)=O)C[C@@](C)(C=C)[C@@H](O)[C@@H]2C	6.72
O=C1CC[C@]2(CC[C@H]3C)[C@]1([H])[C@]3(C)[C@H](OC(CSC4=NC(N5CC[C@@H](O)C5)=CC(C)=N4)=O)C[C@@](C)(C=C)[C@@H](O)[C@@H]2C	6.72
O=C1CC[C@]2(CC[C@H]3C)[C@]1([H])[C@]3(C)[C@H](OC(CSC4=NC(N5CCOCC5)=CC(C)=N4)=O)C[C@@](C)(C=C)[C@@H](O)[C@@H]2C	6.72
O=C(CC1)[C@]2([H])[C@@]31[C@@H](C)[C@H](O)[C@](C)(C=C)C[C@@H](OC(CSCC4=CSC(NC(CN5CCC(O)CC5)=O)=N4)=O)[C@]2(C)[C@H](C)CC3	6.71
O=C(CC1)[C@]2([H])[C@@]31[C@@H](C)[C@H](O)[C@](C)(C=C)C[C@@H](OC(CSCC4=CSC(NC(CN5[C@H](CO)CCC5)=O)=N4)=O)[C@]2(C)[C@H](C)CC3	6.71
O=C(CC1)[C@]2([H])[C@@]31[C@@H](C)[C@H](O)[C@](C)(C=C)C[C@@H](OC(CN4CCN(C(NCC5=CC=C(C(F)(F)F)C=C5)=O)CC4)=O)[C@]2(C)[C@H](C)CC3	6.71
O=C(CC1)[C@]2([H])[C@@]31[C@@H](C)[C@H](O)[C@](C)(C=C)C[C@@H](OC(CSC4=C(N)C=NC=C4)=O)[C@]2(C)[C@H](C)CC3	6.71
O=C(CC1)[C@]2([H])[C@@]31[C@@H](C)[C@H](O)[C@](C)(C=C)C[C@@H](OC(CSC4=CC(N)=NC=C4)=O)[C@]2(C)[C@H](C)CC3	6.71
O=C(CC1)[C@]2([H])[C@@]31[C@@H](C)[C@H](O)[C@](C)(C=C)C[C@@H](OC(CSCC4=CSC(NC(C5=CC=C(OC)C=C5)=O)=N4)=O)[C@]2(C)[C@H](C)CC3	6.71
O=C1CC[C@]2(CC[C@H]3C)[C@]1([H])[C@]3(C)[C@H](OC(CSC4=NC(N5CCCC5)=CC(C)=N4)=O)C[C@@](C)(C=C)[C@@H](O)[C@@H]2C	6.70
O=C1CC[C@]2(CC[C@H]3C)[C@]1([H])[C@]3(C)[C@H](OC(CSC4=NC(N)=CC(NC(CN5CC[C@@H](O)C5)=O)=N4)=O)C[C@@](C)(C=C)[C@@H](O)[C@@H]2C	6.70
O=C(CC1)[C@]2([H])[C@@]31[C@@H](C)[C@H](O)[C@](C)(C=C)C[C@@H](OC(CSC4=NC(NC(CN5C[C@@H](O)CC5)=O)=CC(N)=N4)=O)[C@]2(C)[C@H](C)CC3	6.70
O=C(CC1)[C@]2([H])[C@@]31[C@@H](C)[C@H](O)[C@](C)(C=C)C[C@@H](OC(CSC4=NC=CC=N4)=O)[C@]2(C)[C@H](C)CC3	6.70
O=C(CC1)[C@]2([H])[C@@]31[C@@H](C)[C@H](O)[C@](C)(C=C)C[C@@H](OC(CSC4=CC(NC(CN5CCC[C@H]5CO)=O)=CN=C4)=O)[C@]2(C)[C@H](C)CC3	6.70
O=C(CC1)[C@]2([H])[C@@]31[C@@H](C)[C@H](O)[C@](C)(C=C)C[C@@H](OC(CSC4=CC=NC=C4)=O)[C@]2(C)[C@H](C)CC3	6.70
O=C(CC1)[C@]2([H])[C@@]31[C@@H](C)[C@H](O)[C@](C)(C=C)C[C@@H](OC(CSC4=C(NC(CN5CCC(O)CC5)=O)C=CC=C4)=O)[C@]2(C)[C@H](C)CC3	6.70
O=C(CC1)[C@]2([H])[C@@]31[C@@H](C)[C@H](O)[C@](C)(C=C)C[C@@H](OC(CN4N=NC(C5=CC=C(CN(C(N6)=O)C=C(C)C6=O)C=C5)=C4)=O)[C@]2(C)[C@H](C)CC3	6.69
O=C(CC1)[C@]2([H])[C@@]31[C@@H](C)[C@H](O)[C@](C)(C=C)C[C@@H](OC(CSC4=C(NC(CN5CCC(O)C5)=O)C=CC=C4)=O)[C@]2(C)[C@H](C)CC3	6.69
O=C(CC1)[C@]2([H])[C@@]31[C@@H](C)[C@H](O)[C@](C)(C=C)C[C@@H](OC(CSC4=C(NC(C5=CC=CC(F)=C5)=O)C=CC=C4)=O)[C@]2(C)[C@H](C)CC3	6.69
O=C(CC1)[C@]2([H])[C@@]31[C@@H](C)[C@H](O)[C@](C)(C=C)C[C@@H](OC(CSC4=C(NC(CN(CC)CC)=O)C=CC=C4)=O)[C@]2(C)[C@H](C)CC3	6.68
O=C(CC1)[C@]2([H])[C@@]31[C@@H](C)[C@H](O)[C@](C)(C=C)C[C@@H](OC(CSC4=CC(NC(CN5CCCC5)=O)=CN=C4)=O)[C@]2(C)[C@H](C)CC3	6.68
O=C(CC1)[C@]2([H])[C@@]31[C@@H](C)[C@H](O)[C@](C)(C=C)C[C@@H](OC(CSC4=NN(C(C5=CC=C(C)C=C5)=O)C(N)=N4)=O)[C@]2(C)[C@H](C)CC3	6.68
O=C(CC1)[C@]2([H])[C@@]31[C@@H](C)[C@H](O)[C@](C)(C=C)C[C@@H](OC(CN4CCN(C(/C=C/C5=CC=C(O)C=C5)=O)CC4)=O)[C@]2(C)[C@H](C)CC3	6.68
O=C(CC1)[C@]2([H])[C@@]31[C@@H](C)[C@H](O)[C@](C)(C=C)C[C@@H](OC(CSC4=CC(NC([C@@H](N)C(C)C)=O)=CN=C4)=O)[C@]2(C)[C@H](C)CC3	6.67
O=C(CC1)[C@]2([H])[C@@]31[C@@H](C)[C@H](O)[C@](C)(C=C)C[C@@H](OC(CSC4=NN=C(C5=CC=CC([N+]([O-])=O)=C5)O4)=O)[C@]2(C)[C@H](C)CC3	6.67
O=C(CC1)[C@]2([H])[C@@]31[C@@H](C)[C@H](O)[C@](C)(C=C)C[C@@H](OC(CS(SC4=NC5=C(C=CC=C5)O4)=O)=O)[C@]2(C)[C@H](C)CC3	6.67
O=C(CC1)[C@]2([H])[C@@]31[C@@H](C)[C@H](O)[C@](C)(C=C)C[C@@H](OC(CSC4=NN=C(C5=CC=CC([N+]([O-])=O)=C5)N4)=O)[C@]2(C)[C@H](C)CC3	6.67
O=C(CC1)[C@]2([H])[C@@]31[C@@H](C)[C@H](O)[C@](C)(C=C)C[C@@H](OC(CSC4=NN=C(C5=CC=C([N+]([O-])=O)C=C5)N4)=O)[C@]2(C)[C@H](C)CC3	6.67
O=C(CC1)[C@]2([H])[C@@]31[C@@H](C)[C@H](O)[C@](C)(C=C)C[C@@H](OC(CSC4=NN=C(C5=CC=C(N(C)C)C=C5)O4)=O)[C@]2(C)[C@H](C)CC3	6.67
O=C(CC1)[C@]2([H])[C@@]31[C@@H](C)[C@H](O)[C@](C)(C=C)C[C@@H](OC(N4C(C(C=CC(N5CCN(C)CC5)=C6)=C6C4)=O)=O)[C@]2(C)[C@H](C)CC3	6.66
O=C(CC1)[C@]2([H])[C@@]31[C@@H](C)[C@H](O)[C@](C)(C=C)C[C@@H](OC(CSCCNC(C4=C(Cl)C=CC=C4)=O)=O)[C@]2(C)[C@H](C)CC3	6.66
O=C(CC1)[C@]2([H])[C@@]31[C@@H](C)[C@H](O)[C@](C)(C=C)C[C@@H](OC(CSC4=NN=C(C5=CC=CC=C5Cl)N4)=O)[C@]2(C)[C@H](C)CC3	6.66
O=C(CC1)[C@]2([H])[C@@]31[C@@H](C)[C@H](O)[C@](C)(C=C)C[C@@H](OC(CSC4=NN=C(C5=CC=C(Cl)C=C5)N4)=O)[C@]2(C)[C@H](C)CC3	6.66
O=C(CC1)[C@]2([H])[C@@]31[C@@H](C)[C@H](O)[C@](C)(C=C)C[C@@H](OC(CSCCNC(C4=C(OC)C=CC=C4)=O)=O)[C@]2(C)[C@H](C)CC3	6.66
O=C(CC1)[C@]2([H])[C@@]31[C@@H](C)[C@H](O)[C@](C)(C=C)C[C@@H](OC(CSC4=NN=C(C5=CC=CC(OC)=C5)N4)=O)[C@]2(C)[C@H](C)CC3	6.66
O=C(CC1)[C@]2([H])[C@@]31[C@@H](C)[C@H](O)[C@](C)(C=C)C[C@@H](OC(CSC4=NN=C(C5=CC=C(OC)C=C5)N4)=O)[C@]2(C)[C@H](C)CC3	6.66
O=C1CC[C@]2(CC[C@H]3C)[C@]1([H])[C@]3(C)[C@H](OC(CN4CCN(C5=CC=CC=C5[N+]([O-])=O)CC4)=O)C[C@@](C)(C=C)[C@@H](O)[C@@H]2C	6.66
O=C(CC1)[C@]2([H])[C@@]31[C@@H](C)[C@H](O)[C@](C)(C=C)C[C@@H](OC(CSCCNC(C4=CC=CC=C4F)=O)=O)[C@]2(C)[C@H](C)CC3	6.65
O=C(CC1)[C@]2([H])[C@@]31[C@@H](C)[C@H](O)[C@](C)(C=C)C[C@@H](OC(CSCCNC(C4=CC=C(F)C=C4)=O)=O)[C@]2(C)[C@H](C)CC3	6.65
O=C(CC1)[C@]2([H])[C@@]31[C@@H](C)[C@H](O)[C@](C)(C=C)C[C@@H](OC(CSC4=NC(N)=CC(NC(CC)=O)=N4)=O)[C@]2(C)[C@H](C)CC3	6.65
O=C(CC1)[C@]2([H])[C@@]31[C@@H](C)[C@H](O)[C@](C)(C=C)C[C@@H](OC(CSC4=CC(NC([C@H](N)C)=O)=CN=C4)=O)[C@]2(C)[C@H](C)CC3	6.65
O=C(CC1)[C@]2([H])[C@@]31[C@@H](C)[C@H](O)[C@](C)(C=C)C[C@@H](OC(CSC4=NN=C(C5=CC=CC=C5F)O4)=O)[C@]2(C)[C@H](C)CC3	6.65
O=C(CC1)[C@]2([H])[C@@]31[C@@H](C)[C@H](O)[C@](C)(C=C)C[C@@H](OC(CSC4=NN=C(C5=CC=CC(F)=C5)O4)=O)[C@]2(C)[C@H](C)CC3	6.65

O=C(CC1)[C@]2([H])[C@@]31[C@@H](C)[C@H](O)[C@](C)(C=C)C[C@@H](OC(CSCCNC(C4=C(C)C=CC=C4)=O)=O)[C@]2(C)[C@H](C)CC3	6.65
O=C(CC1)[C@]2([H])[C@@]31[C@@H](C)[C@H](O)[C@](C)(C=C)C[C@@H](OC(CSCCNC(C4=CC=C(C)C=C4)=O)=O)[C@]2(C)[C@H](C)CC3	6.65
O=C(CC1)[C@]2([H])[C@@]31[C@@H](C)[C@H](O)[C@](C)(C=C)C[C@@H](OC(CSC4=NN=C(C5=CC=CC(F)=C5)N4)=O)[C@]2(C)[C@H](C)CC3	6.65
O=C(CC1)[C@]2([H])[C@@]31[C@@H](C)[C@H](O)[C@](C)(C=C)C[C@@H](OC(CSC4=NN=C(C5=CC=C(F)C=C5)N4)=O)[C@]2(C)[C@H](C)CC3	6.65
O=C(CC1)[C@]2([H])[C@@]31[C@@H](C)[C@H](O)[C@](C)(C=C)C[C@@H](OC(CNC4=CC=CC(Br)=C4)=O)[C@]2(C)[C@H](C)CC3	6.65
O=C(CC1)[C@]2([H])[C@@]31[C@@H](C)[C@H](O)[C@](C)(C=C)C[C@@H](OC(CNC4=CC=C(Br)C=C4)=O)[C@]2(C)[C@H](C)CC3	6.65
O=C(CC1)[C@]2([H])[C@@]31[C@@H](C)[C@H](O)[C@](C)(C=C)C[C@@H](OC(CSC4=NN=C(C5=CC=CC=C5O)O4)=O)[C@]2(C)[C@H](C)CC3	6.65
O=C(CC1)[C@]2([H])[C@@]31[C@@H](C)[C@H](O)[C@](C)(C=C)C[C@@H](OC(CSC4=NN=C(C5=CC=C(O)C=C5)O4)=O)[C@]2(C)[C@H](C)CC3	6.65
O=C(CC1)[C@]2([H])[C@@]31[C@@H](C)[C@H](O)[C@](C)(C=C)C[C@@H](OC(CSC4=NN=C(C5=CC=C(N)C=C5)O4)=O)[C@]2(C)[C@H](C)CC3	6.65
O=C(CC1)[C@]2([H])[C@@]31[C@@H](C)[C@H](O)[C@](C)(C=C)C[C@@H](OC(CSC4=NN=C(C5=CC=CC(C)=C5)O4)=O)[C@]2(C)[C@H](C)CC3	6.65
O=C(CC1)[C@]2([H])[C@@]31[C@@H](C)[C@H](O)[C@](C)(C=C)C[C@@H](OC(CSC4=NN=C(C5=CC=C(C)C=C5)N4)=O)[C@]2(C)[C@H](C)CC3	6.64
O=C(CC1)[C@]2([H])[C@@]31[C@@H](C)[C@H](O)[C@](C)(C=C)C[C@@H](OC(CS(SC4=NC(O)=CC(C)=N4)=O)=O)[C@]2(C)[C@H](C)CC3	6.64
O=C1CC[C@]2(CC[C@H]3C)[C@]1([H])[C@]3(C)[C@H](OC(CSC4=NC(O)=CC(NC(C)=O)=N4)=O)C[C@@](C)(C=C)[C@@H](O)[C@@H]2C	6.64
O=C(CC1)[C@]2([H])[C@@]31[C@@H](C)[C@H](O)[C@](C)(C=C)C[C@@H](OC(CSC4=NN=C(C5=CC=CS5)O4)=O)[C@]2(C)[C@H](C)CC3	6.64
O=C(CC1)[C@]2([H])[C@@]31[C@@H](C)[C@H](O)[C@](C)(C=C)C[C@@H](OC(CSC4=NN=C(C5=CC=NC=C5)O4)=O)[C@]2(C)[C@H](C)CC3	6.64
O=C(CC1)[C@]2([H])[C@@]31[C@@H](C)[C@H](O)[C@](C)(C=C)C[C@@H](OC(CN4CCN(C5=CC=C(C)C=C5)CC4)=O)[C@]2(C)[C@H](C)CC3	6.63
O=C(CC1)[C@]2([H])[C@@]31[C@@H](C)[C@H](O)[C@](C)(C=C)C[C@@H](OC(CSC4=NN=C(C5=CC=CO5)O4)=O)[C@]2(C)[C@H](C)CC3	6.63
O=C(CC1)[C@]2([H])[C@@]31[C@@H](C)[C@H](O)[C@](C)(C=C)C[C@@H](OC(CN4CCN(C5=CC=CC=C5)CC4)=O)[C@]2(C)[C@H](C)CC3	6.62
O=C(CC1)[C@]2([H])[C@@]31[C@@H](C)[C@H](O)[C@](C)(C=C)C[C@@H](OC(CSC4=CC(C=NO)=CC=N4)=O)[C@]2(C)[C@H](C)CC3	6.61
O=C(CC1)[C@]2([H])[C@@]31[C@@H](C)[C@H](O)[C@](C)(C=C)C[C@@H](OC(CSC4=NC=NC5=C4C=NN5)=O)[C@]2(C)[C@H](C)CC3	6.61
O=C(CC1)[C@]2([H])[C@@]31[C@@H](C)[C@H](O)[C@](C)(C=C)C[C@@H](OC(CSC4=NC=NC5=C4C=NN5)=O)[C@]2(C)[C@H](C)CC3	6.61
O=C(CC1)[C@]2([H])[C@@]31[C@@H](C)[C@H](O)[C@](C)(C=C)C[C@@H](OC(CNC4=CC=CC(Cl)=C4)=O)[C@]2(C)[C@H](C)CC3	6.61
O=C(CC1)[C@]2([H])[C@@]31[C@@H](C)[C@H](O)[C@](C)(C=C)C[C@@H](OC(CNC4=CC=C(Cl)C=C4)=O)[C@]2(C)[C@H](C)CC3	6.61
O=C(CC1)[C@]2([H])[C@@]31[C@@H](C)[C@H](O)[C@](C)(C=C)C[C@@H](OC(CSCC4=CSC(N)=N4)=O)[C@]2(C)[C@H](C)CC3	6.61
O=C(CC1)[C@]2([H])[C@@]31[C@@H](C)[C@H](O)[C@](C)(C=C)C[C@@H](OC(CSC4=NC(N)=CC(O)=N4)=O)[C@]2(C)[C@H](C)CC3	6.61
O=C1CC[C@]2(CC[C@H]3C)[C@]1([H])[C@]3(C)[C@H](OC(CSC4=NC(N)=CC(N)=N4)=O)C[C@@](C)(C=C)[C@@H](O)[C@@H]2C	6.60
O=C(CC1)[C@]2([H])[C@@]31[C@@H](C)[C@H](O)[C@](C)(C=C)C[C@@H](OC(CSC4=NC(N)=CC(N)=N4)=O)[C@]2(C)[C@H](C)CC3	6.60
O=C(CC1)[C@]2([H])[C@@]31[C@@H](C)[C@H](O)[C@](C)(C=C)C[C@@H](OC(CSC4=C(CO)C=CC=N4)=O)[C@]2(C)[C@H](C)CC3	6.60
O=C(CC1)[C@]2([H])[C@@]31[C@@H](C)[C@H](O)[C@](C)(C=C)C[C@@H](OC(N4C(C(C=C(F)C=C5)=C5C4)=O)=O)[C@]2(C)[C@H](C)CC3	6.60
O=C(CC1)[C@]2([H])[C@@]31[C@@H](C)[C@H](O)[C@](C)(C=C)C[C@@H](OC(CN4CCN(C(/C=C/C5=CC=CC=C5[N+]([O-])=O)=O)CC4)=O)[C@]2(C)[C@H](C)CC3	6.60
O=C(CC1)[C@]2([H])[C@@]31[C@@H](C)[C@H](O)[C@](C)(C=C)C[C@@H](OC(CSC4=NC=CC(N)=N4)=O)[C@]2(C)[C@H](C)CC3	6.59
O=C(CC1)[C@]2([H])[C@@]31[C@@H](C)[C@H](O)[C@](C)(C=C)C[C@@H](OC(CSC4=NC=CC(N)=N4)=O)[C@]2(C)[C@H](C)CC3	6.59
O=C(CC1)[C@]2([H])[C@@]31[C@@H](C)[C@H](O)[C@](C)(C=C)C[C@@H](OC(N4C(C(C=CC=C5)=C5C4)=O)=O)[C@]2(C)[C@H](C)CC3	6.58
O=C(CC1)[C@]2([H])[C@@]31[C@@H](C)[C@H](O)[C@](C)(C=C)C[C@@H](OC(CSC4CCN(C(CCN5C=NC6=C5N=C(N)N=C6N7CCOCC7)=O)CC4)=O)[C@]2(C)[C@H](C)CC3	6.58
O=C(CC1)[C@]2([H])[C@@]31[C@@H](C)[C@H](O)[C@](C)(C=C)C[C@@H](OC(CN4CCN(C)CC4)=O)[C@]2(C)[C@H](C)CC3	6.57
O=C(CC1)[C@]2([H])[C@@]31[C@@H](C)[C@H](O)[C@](C)(C=C)C[C@@H](OC(CSC4CCN(C(CCN5C=NC6=C5N=C(N)N=C6NC)=O)CC4)=O)[C@]2(C)[C@H](C)CC3	6.54
O=C(CC1)[C@]2([H])[C@@]31[C@@H](C)[C@H](O)[C@](C)(C=C)C[C@@H](OC(CSC4CCN(C(CCCN5C=NC6=C5N=C(N)N=C6)=O)CC4)=O)[C@]2(C)[C@H](C)CC3	6.53
O=C(CC1)[C@]2([H])[C@@]31[C@@H](C)[C@H](O)[C@](C)(C=C)C[C@@H](OC(CSC4=NC(C=CC(N)=C5)=C5N4)=O)[C@]2(C)[C@H](C)CC3	6.53
O=C(CC1)[C@]2([H])[C@@]31[C@@H](C)[C@H](O)[C@](C)(C=C)C[C@@H](OC(CSC4CCN(C(/C=C/N5C=NC6=C5N=C(N)N=C6)=O)CC4)=O)[C@]2(C)[C@H](C)CC3	6.52
O=C(CC1)[C@]2([H])[C@@]31[C@@H](C)[C@H](O)[C@](C)(C=C)C[C@@H](OC(CSCC4=CC(C(O)=CO4)=O)=O)[C@]2(C)[C@H](C)CC3	6.52
O=C(CC1)[C@]2([H])[C@@]31[C@@H](C)[C@H](O)[C@](C)(C=C)C[C@@H](OC(CSC4=C([N+]([O-])=O)C=CC=N4)=O)[C@]2(C)[C@H](C)CC3	6.52
O=C(CC1)[C@]2([H])[C@@]31[C@@H](C)[C@H](O)[C@](C)(C=C)C[C@@H](OC(CSC4=CC(NC(CN5CC[C@@H](O)C5)=O)=CN=C4)=O)[C@]2(C)[C@H](C)CC3	6.52
O=C(CC1)[C@]2([H])[C@@]31[C@@H](C)[C@H](O)[C@](C)(C=C)C[C@@H](OC(CSC4=C(CO)C=NC=C4)=O)[C@]2(C)[C@H](C)CC3	6.51
O=C(CC1)[C@]2([H])[C@@]31[C@@H](C)[C@H](O)[C@](C)(C=C)C[C@@H](OC(CSC4CCN(C(CN5C=NC6=C5N=CN=C6)=O)CC4)=O)[C@]2(C)[C@H](C)CC3	6.50
O=C(CC1)[C@]2([H])[C@@]31[C@@H](C)[C@H](O)[C@](C)(C=C)C[C@@H](OC(CSC4=CC(NC([C@H]5CCCCN5)=O)=CN=C4)=O)[C@]2(C)[C@H](C)CC3	6.50
O=C(CC1)[C@]2([H])[C@@]31[C@@H](C)[C@H](O)[C@](C)(C=C)C[C@@H](OC(CSC4CCN(C(CCN5C=NC6=C5N=C(N)N=C6N7CCC(N(C)C)CC7)=O)CC4)=O)[C@]2(C)[C@H](C)CC3	6.50
O=C(CC1)[C@]2([H])[C@@]31[C@@H](C)[C@H](O)[C@](C)(C=C)C[C@@H](OC(CSC4=C(NC(CN5N=NC(CN6CCN(C7=CC=CC(O)=C7)CC6)=C5)=O)C=CC=C4)=O)[C@]2(C)[C@H](C)CC3	6.50
O=C(CC1)[C@]2([H])[C@@]31[C@@H](C)[C@H](O)[C@](C)(C=C)C[C@@H](OC(CSC4=CC(O)=CN=C4)=O)[C@]2(C)[C@H](C)CC3	6.49
O=C(CC1)[C@]2([H])[C@@]31[C@@H](C)[C@H](O)[C@](C)(C=C)C[C@@H](OC(CSC4=CC(NC([C@H]5CCCN5)=O)=CN=C4)=O)[C@]2(C)[C@H](C)CC3	6.49
O=C1CC[C@]2(CC[C@H]3C)[C@]1([H])[C@]3(C)[C@H](OC(CSC4=NC(N5CCN(CCO)CC5)=CC(C)=N4)=O)C[C@@](C)(C=C)[C@@H](O)[C@@H]2C	6.49
O=C1CC[C@]2(CC[C@H]3C)[C@]1([H])[C@]3(C)[C@H](OC(CSC4=NC(N5CCC(CCO)CC5)=CC(C)=N4)=O)C[C@@](C)(C=C)[C@@H](O)[C@@H]2C	6.49
O=C(CC1)[C@]2([H])[C@@]31[C@@H](C)[C@H](O)[C@](C)(CC)C[C@@H](OC(CSC(CC4)CCN4C(CCN5C=NC6=C5N=C(N)N=C6N7CCNCC7)=O)=O)[C@]2(C)[C@H](C)CC3	6.49
O=C(CC1)[C@]2([H])[C@@]31[C@@H](C)[C@H](O)[C@](C)(C=C)C[C@@H](OC(CSC4CCN(C(CCN5C=NC6=C5N=C(N)N=C6N7CC(C)NCC7)=O)CC4)=O)[C@]2(C)[C@H](C)CC3	6.49
O=C(CC1)[C@]2([H])[C@@]31[C@@H](C)[C@H](O)[C@](C)(C=C)C[C@@H](OC(CSC4CCN(C(CCN5C=NC6=C5N=CN=C6N7CC(NC)CC7)=O)CC4)=O)[C@]2(C)[C@H](C)CC3	6.49
O=C(CC1)[C@]2([H])[C@@]31[C@@H](C)[C@H](O)[C@](C)(C=C)C[C@@H](OC(CSC4CCN(C(CCN5C=NC6=C5N=C(N)N=C6N7CC(N(C)C)CC7)=O)CC4)=O)[C@]2(C)[C@H](C)CC3	6.49
O=C(CC1)[C@]2([H])[C@@]31[C@@H](C)[C@H](O)[C@](C)(C=C)C[C@@H](OC(CSC4CCN(C(CCN5C=NC6=C5N=CN=C6N7CCC(NC)CC7)=O)CC4)=O)[C@]2(C)[C@H](C)CC3	6.49
O=C(CC1)[C@]2([H])[C@@]31[C@@H](C)[C@H](O)[C@](C)(C=C)C[C@@H](OC(CSC4=CC(C(NC5=CC=C(S(=O)(NC6=NC=CS6)=O)C=C5)=O)=CC=C4)=O)[C@]2(C)[C@H](C)CC3	6.48

O=C(CC1)[C@]2([H])[C@@]31[C@@H](C)[C@H](O)[C@](C)(C=C)C[C@@H](OC(CSC4CCN(C(CCN5C=NC6=C5N=CN=C6N7CCC(N)CC7)=O)CC4)=O)[C@]2(C)[C@H](C)CC3	6.48
O=C(CC1)[C@]2([H])[C@@]31[C@@H](C)[C@H](O)[C@](C)(C=C)C[C@@H](OC(CS[C@H]4CC[C@H](C(OCC)=O)C[C@H]4NS(=O)(C5=CC=C(C(C)=O)C=C5)=O)=O)[C@]2(C)[C@H](C)CC3	6.47
O=C(CC1)[C@]2([H])[C@@]31[C@@H](C)[C@H](O)[C@](C)(C=C)C[C@@H](OC(CSC4CCN(C(CCN5C=NC6=C5N=CN=C6N7C[C@H](N)CC7)=O)CC4)=O)[C@]2(C)[C@H](C)CC3	6.47
O=C(CC1)[C@]2([H])[C@@]31[C@@H](C)[C@H](O)[C@](C)(C=C)C[C@@H](OC(CN4N=NC(C5=CC=C(CN6C7=NC=NC(N8CCN(C)CC8)=C7N=C6)C=C5)=C4)=O)[C@]2(C)[C@H](C)CC3	6.47
O=C(CC1)[C@]2([H])[C@@]31[C@@H](C)[C@H](O)[C@](C)(C=C)C[C@@H](OC(CS[C@H]4CC[C@H](C(OCC)=O)C[C@H]4NS(=O)(C5=CC=C(OC)C=C5)=O)=O)[C@]2(C)[C@H](C)CC3	6.47
O=C(CC1)[C@]2([H])[C@@]31[C@@H](C)[C@H](O)[C@](C)(C=C)C[C@@H](OC(CSCCCN(C(CCN4C=NC5=C4N=CN=C5N6CCC(N)C6)=O)C)=O)[C@]2(C)[C@H](C)CC3	6.46
O=C1CC[C@]2(CC[C@H]3C)[C@]1([H])[C@]3(C)[C@H](OC(CSC4=NC(N5CCC[C@H](CO)C5)=CC(C)=N4)=O)C[C@@](C)(C=C)[C@@H](O)[C@@H]2C	6.46
O=C(N(CC1)CCN1CC(O[C@@H]([C@]2(C)[C@H](C)CC3)C[C@@](C)(C=C)[C@@H](O)[C@H](C)[C@@]43[C@@]2([H])C(CC4)=O)=O)[C@H](CC5=CC=C(O)C=C5)NC(OC(C)(C)C)=O	6.45
O=C(CC1)[C@]2([H])[C@@]31[C@@H](C)[C@H](O)[C@](C)(C=C)C[C@@H](OC(CN4N=NC(C5=CC=C(CN6C7=NC=NC(N(CC)CC)=C7N=C6)C=C5)=C4)=O)[C@]2(C)[C@H](C)CC3	6.45
O=C(CC1)[C@]2([H])[C@@]31[C@@H](C)[C@H](O)[C@](C)(C=C)C[C@@H](OC(CSC4=C(NC(CN5N=NC(CN6CCN(C)CC6)=C5)=O)C=CC=C4)=O)[C@]2(C)[C@H](C)CC3	6.45
O=C1CC[C@]2(CC[C@H]3C)[C@]1([H])[C@]3(C)[C@H](OC(CSC4=NC(N5CCC[C@@H](O)C5)=CC(C)=N4)=O)C[C@@](C)(C=C)[C@@H](O)[C@@H]2C	6.45
O=C1CC[C@]2(CC[C@H]3C)[C@]1([H])[C@]3(C)[C@H](OC(CSC4=NC(N5CCC(O)CC5)=CC(C)=N4)=O)C[C@@](C)(C=C)[C@@H](O)[C@@H]2C	6.45
O=C(CC1)[C@]2([H])[C@@]31[C@@H](C)[C@H](O)[C@](C)(C=C)C[C@@H](OC(CN4CCN(C(CN5CCN(C6=CC=CC=C6[N+]([O-])=O)CC5)=O)CC4)=O)[C@]2(C)[C@H](C)CC3	6.44
O=C(CC1)[C@]2([H])[C@@]31[C@@H](C)[C@H](O)[C@](C)(C=C)C[C@@H](OC(CN4CCN(C(CN5CCN(C6=CC=CC([N+]([O-])=O)=C6)CC5)=O)CC4)=O)[C@]2(C)[C@H](C)CC3	6.44
CC1C23C(C(CC3)=O)C(C(CC(C=C)(C)C1O)OC(CN4CCN(C(CN5CCN(C6=CC([N+]([O-])=O)=CC=C6)CC5)=O)CC4)=O)(C(CC2)C)C	6.44
O=C(CC1)[C@]2([H])[C@@]31[C@@H](C)[C@H](O)[C@](C)(C=C)C[C@@H](OC(CSC4=C(NC(CN5CCN(C6=CC=CC=C6)CC5)=O)C=CC=C4)=O)[C@]2(C)[C@H](C)CC3	6.44
O=C(CC1)[C@]2([H])[C@@]31[C@@H](C)[C@H](O)[C@](C)(C=C)C[C@@H](OC(CN4CCN(C(CN5CCN(C6=CC=C(Cl)C=C6)CC5)=O)CC4)=O)[C@]2(C)[C@H](C)CC3	6.44
CC1C23C(C(CC3)=O)C(C(CC(C=C)(C)C1O)OC(CN4CCN(C(CN5CCN(C6=CC(Cl)=CC=C6)CC5)=O)CC4)=O)(C(CC2)C)C	6.44
O=C1CC[C@]2(CC[C@H]3C)[C@]1([H])[C@]3(C)[C@H](OC(CSC4=NC(NC=C5)=C5C(NC(CN6CCC(CO)CC6)=O)=N4)=O)C[C@@](C)(C=C)[C@@H](O)[C@@H]2C	6.44
O=C(CC1)[C@]2([H])[C@@]31[C@@H](C)[C@H](O)[C@](C)(C=C)C[C@@H](OC(CSC4=C(NC(CN5N=NC(CN(CC)CC)=C5)=O)C=CC=C4)=O)[C@]2(C)[C@H](C)CC3	6.43
CC1C23C(C(CC3)=O)C(C(CC(C=C)(C)C1O)OC(CN4CCN(C(CN5CCN(C6=CC(OC)=CC=C6)CC5)=O)CC4)=O)(C(CC2)C)C	6.43
O=C(CC1)[C@]2([H])[C@@]31[C@@H](C)[C@H](O)[C@](C)(C=C)C[C@@H](OC(CSC4=C(NC(CN5N=NC(CN6CCCC6)=C5)=O)C=CC=C4)=O)[C@]2(C)[C@H](C)CC3	6.43
O=C(CC1)[C@]2([H])[C@@]31[C@@H](C)[C@H](O)[C@](C)(C=C)C[C@@H](OC(CN4CCN(C(/C=C/C5=C(F)C=C(Br)C=C5)=O)CC4)=O)[C@]2(C)[C@H](C)CC3	6.43
O=C1CC[C@]2(CC[C@H]3C)[C@]1([H])[C@]3(C)[C@H](OC(CSC4=NC(NC=C5)=C5C(NC(CN6CCC(O)CC6)=O)=N4)=O)C[C@@](C)(C=C)[C@@H](O)[C@@H]2C	6.43
O=C(CC1)[C@]2([H])[C@@]31[C@@H](C)[C@H](O)[C@](C)(C=C)C[C@@H](OC(CN4CCN(C(CN5CCN(C6=CC=CC(O)=C6)CC5)=O)CC4)=O)[C@]2(C)[C@H](C)CC3	6.42
O=C(CC1)[C@]2([H])[C@@]31[C@@H](C)[C@H](O)[C@](C)(C=C)C[C@@H](OC(CN4N=NC(C5=CC=C(CN6C(C(C=CC=C7)=C7C6=O)=O)C=C5)=C4)=O)[C@]2(C)[C@H](C)CC3	6.42
CC1C23C(C(CC3)=O)C(C(CC(C=C)(C)C1O)OC(CN4CCN(C(CN5CCN(C6=C(C)C=CC=C6)CC5)=O)CC4)=O)(C(CC2)C)C	6.42
CC1C23C(C(CC3)=O)C(C(CC(C=C)(C)C1O)OC(CN4CCN(C(CN5CCN(C6=CC=C(C)C=C6)CC5)=O)CC4)=O)(C(CC2)C)C	6.42
O=C(CC1)[C@]2([H])[C@@]31[C@@H](C)[C@H](O)[C@](C)(C=C)C[C@@H](OC(N4C(C(C=CC(N)=C5)=C5C4)=O)=O)[C@]2(C)[C@H](C)CC3	6.42
O=C(CC1)[C@]2([H])[C@@]31[C@@H](C)[C@H](O)[C@](C)(C=C)C[C@@H](OC(CSC4=NN=C(C)N4/N=C/C5=CC=C(C6=CC=CC=N6)C=C5)=O)[C@]2(C)[C@H](C)CC3	6.42
O=C(CC1)[C@]2([H])[C@@]31[C@@H](C)[C@H](O)[C@](C)(C=C)C[C@@H](OC(CSCC4=CSC(NC(C5=CC=CC=C5[N+]([O-])=O)=O)=N4)=O)[C@]2(C)[C@H](C)CC3	6.42
O=C1CC[C@]2(CC[C@H]3C)[C@]1([H])[C@]3(C)[C@H](OC(CSC4=NC(NC=C5)=C5C(NC(CN6CCOCC6)=O)=N4)=O)C[C@@](C)(C=C)[C@@H](O)[C@@H]2C	6.42
O=C(CC1)[C@]2([H])[C@@]31[C@@H](C)[C@H](O)[C@](C)(C=C)C[C@@H](OC(CN4N=NC(C5=CC=CC(CN6C7=NC=NC(N)=C7N=C6)=C5)=C4)=O)[C@]2(C)[C@H](C)CC3	6.42
O=C(CC1)[C@]2([H])[C@@]31[C@@H](C)[C@H](O)[C@](C)(C=C)C[C@@H](OC(CSC4=C(NC(CN5N=NC(CN(C)C)=C5)=O)C=CC=C4)=O)[C@]2(C)[C@H](C)CC3	6.42
CC1C23C(C(CC3)=O)C(C(CC(C=C)(C)C1O)OC(CN4CCN(C(CN5CCN(C6=CC=CC=C6)CC5)=O)CC4)=O)(C(CC2)C)C	6.41
O=C1CC[C@]2(CC[C@H]3C)[C@]1([H])[C@]3(C)[C@H](OC(CSC4=NC(N5CCNCC5)=CC(C)=N4)=O)C[C@@](C)(C=C)[C@@H](O)[C@@H]2C	6.41
O=C1CC[C@]2(CC[C@H]3C)[C@]1([H])[C@]3(C)[C@H](OC(CSCC(NC4=CC=C(NCC5=CC=CC=C5)C=C4C)=O)=O)C[C@@](C)(C=C)[C@@H](O)[C@@H]2C	6.41
O=C1CC[C@]2(CC[C@H]3C)[C@]1([H])[C@]3(C)[C@H](OC(CSC4=NC(N)=CC(NC(CN5CCC(O)CC5)=O)=N4)=O)C[C@@](C)(C=C)[C@@H](O)[C@@H]2C	6.41
O=C(CC1)[C@]2([H])[C@@]31[C@@H](C)[C@H](O)[C@](C)(C=C)C[C@@H](OC(CSC4=NC(NC(CN5CCC(O)CC5)=O)=CC(N)=N4)=O)[C@]2(C)[C@H](C)CC3	6.41
O=C(CC1)[C@]2([H])[C@@]31[C@@H](C)[C@H](O)[C@](C)(C=C)C[C@@H](OC(N4CC5=C(C=CC=C5OCCN6C7=CC=CN=C7N=C6)C4=O)=O)[C@]2(C)[C@H](C)CC3	6.41
O=C(CC1)[C@]2([H])[C@@]31[C@@H](C)[C@H](O)[C@](C)(C=C)C[C@@H](OC(CSC4=NC=CN=C4)=O)[C@]2(C)[C@H](C)CC3	6.40
O=C1CC[C@]2(CC[C@H]3C)[C@]1([H])[C@]3(C)[C@H](OC(CSC4=NC(N)=CC(NC(CN5CCOCC5)=O)=N4)=O)C[C@@](C)(C=C)[C@@H](O)[C@@H]2C	6.40
O=C(CC1)[C@]2([H])[C@@]31[C@@H](C)[C@H](O)[C@](C)(C=C)C[C@@H](OC(CSC4=NC(NC(CN5CCOCC5)=O)=CC(N)=N4)=O)[C@]2(C)[C@H](C)CC3	6.40
O=C1CC[C@]2(CC[C@H]3C)[C@]1([H])[C@]3(C)[C@H](OC(CSC4=NC(N)=CC(NC(CN5CCCCC5)=O)=N4)=O)C[C@@](C)(C=C)[C@@H](O)[C@@H]2C	6.40
O=C(CC1)[C@]2([H])[C@@]31[C@@H](C)[C@H](O)[C@](C)(C=C)C[C@@H](OC(CSC4=NC(NC(CN5CCCCC5)=O)=CC(N)=N4)=O)[C@]2(C)[C@H](C)CC3	6.40
O=C(CC1)[C@]2([H])[C@@]31[C@@H](C)[C@H](O)[C@](C)(C=C)C[C@@H](OC(CSCC4=CSC(NC(CC5=CC=CC=C5)=O)=N4)=O)[C@]2(C)[C@H](C)CC3	6.40
O=C1CC[C@]2(CC[C@H]3C)[C@]1([H])[C@]3(C)[C@H](OC(CN4CCN(C(NCC5=CC=C([N+]([O-])=O)C=C5)=O)CC4)=O)C[C@@](C)(C=C)[C@@H](O)[C@@H]2C	6.40
O=C(CC1)[C@]2([H])[C@@]31[C@@H](C)[C@H](O)[C@](C)(C=C)C[C@@H](OC(CSC4=C(NC(C5=CC=CC(Cl)=C5)=O)C=CC=C4)=O)[C@]2(C)[C@H](C)CC3	6.40
O=C1CC[C@]2(CC[C@H]3C)[C@]1([H])[C@]3(C)[C@H](OC(CSC4=NC(NC=C5)=C5C(NC(CNC6CC6)=O)=N4)=O)C[C@@](C)(C=C)[C@@H](O)[C@@H]2C	6.40
O=C(CC1)[C@]2([H])[C@@]31[C@@H](C)[C@H](O)[C@](C)(C=C)C[C@@H](OC(CSCC4=CSC(NC(CN(C)CCO)=O)=N4)=O)[C@]2(C)[C@H](C)CC3	6.40

O=C(CC1)[C@]2([H])[C@@]31[C@@H](C)[C@H](O)[C@](C)(C=C)C[C@@H](OC(CSCC4=CSC(NC(CN5CCCC5)=O)=N4)=O)[C@]2(C)[C@H](C)CC3	6.39
O=C(CC1)[C@]2([H])[C@@]31[C@@H](C)[C@H](O)[C@](C)(C=C)C[C@@H](OC(CSC4=NN=C(C5=CC=CC(Br)=C5)O4)=O)[C@]2(C)[C@H](C)CC3	6.39
O=C(CC1)[C@]2([H])[C@@]31[C@@H](C)[C@H](O)[C@](C)(C=C)C[C@@H](OC(CSCC4=CC(C(O)=C(CN5CCCCC5)O4)=O)=O)[C@]2(C)[C@H](C)CC3	6.39
O=C(CC1)[C@]2([H])[C@@]31[C@@H](C)[C@H](O)[C@](C)(C=C)C[C@@H](OC(CSC4=NN(C(C5=CC=CC(Cl)=C5)=O)C(N)=N4)=O)[C@]2(C)[C@H](C)CC3	6.39
O=C(CC1)[C@]2([H])[C@@]31[C@@H](C)[C@H](O)[C@](C)(C=C)C[C@@H](OC(CSC4=NN=C(NC(C5=CC=CC(N)=C5)=O)S4)=O)[C@]2(C)[C@H](C)CC3	6.39
O=C(CC1)[C@]2([H])[C@@]31[C@@H](C)[C@H](O)[C@](C)(C=C)C[C@@H](OC(CSC4=NN(C(C5=CC=C(OC)C=C5)=O)C(N)=N4)=O)[C@]2(C)[C@H](C)CC3	6.39
O=C(CC1)[C@]2([H])[C@@]31[C@@H](C)[C@H](O)[C@](C)(C=C)C[C@@H](OC(CSC4=NN(C(C5=CC=CC(OC)=C5)=O)C(N)=N4)=O)[C@]2(C)[C@H](C)CC3	6.39
O=C(CC1)[C@]2([H])[C@@]31[C@@H](C)[C@H](O)[C@](C)(C=C)C[C@@H](OC(CN4CCN(C(/C=C/C5=CC(OC)=CC=C5)=O)CC4)=O)[C@]2(C)[C@H](C)CC3	6.39
O=C(CC1)[C@]2([H])[C@@]31[C@@H](C)[C@H](O)[C@](C)(C=C)C[C@@H](OC(CSC4=NN=C(C5=CC=CC=C5C(F)(F)F)N4)=O)[C@]2(C)[C@H](C)CC3	6.38
O=C(CC1)[C@]2([H])[C@@]31[C@@H](C)[C@H](O)[C@](C)(C=C)C[C@@H](OC(CSC4=C(NC(C5=C(C)C=CC=C5)=O)C=CC=C4)=O)[C@]2(C)[C@H](C)CC3	6.38
O=C(CC1)[C@]2([H])[C@@]31[C@@H](C)[C@H](O)[C@](C)(C=C)C[C@@H](OC(CSC4=CC(NC([C@@H](N)[C@@H](C)CC)=O)=CN=C4)=O)[C@]2(C)[C@H](C)CC3	6.38
O=C(CC1)[C@]2([H])[C@@]31[C@@H](C)[C@H](O)[C@](C)(C=C)C[C@@H](OC(CSC(C)(C)CNC(C4=C(OC)C=CC=C4)=O)=O)[C@]2(C)[C@H](C)CC3	6.38
O=C(CC1)[C@]2([H])[C@@]31[C@@H](C)[C@H](O)[C@](C)(C=C)C[C@@H](OC(CSC(C)(C)CNC(C4=CC=C(OC)C=C4)=O)=O)[C@]2(C)[C@H](C)CC3	6.38
O=C1CC[C@]2(CC[C@H]3C)[C@]1([H])[C@]3(C)[C@H](OC(CSCC(NC4=CC=C(NCCC)C=C4C)=O)=O)C[C@@](C)(C=C)[C@@H](O)[C@@H]2C	6.38
O=C1CC[C@]2(CC[C@H]3C)[C@]1([H])[C@]3(C)[C@H](OC(CSCC(NC4=CC=C(NC(C)C)C=C4C)=O)=O)C[C@@](C)(C=C)[C@@H](O)[C@@H]2C	6.38
O=C(CC1)[C@]2([H])[C@@]31[C@@H](C)[C@H](O)[C@](C)(C=C)C[C@@H](OC(CSC4=NN(C(C5=CC=CC(F)=C5)=O)C(N)=N4)=O)[C@]2(C)[C@H](C)CC3	6.38
O=C(CC1)[C@]2([H])[C@@]31[C@@H](C)[C@H](O)[C@](C)(C=C)C[C@@H](OC(CSC(C)(C)CNC([C@H](C)C4=CC=CC=C4)=O)=O)[C@]2(C)[C@H](C)CC3	6.38
O=C(CC1)[C@]2([H])[C@@]31[C@@H](C)[C@H](O)[C@](C)(C=C)C[C@@H](OC(CSC4=C(NC(CN5CCCC5)=O)C=CC=C4)=O)[C@]2(C)[C@H](C)CC3	6.38
O=C(CC1)[C@]2([H])[C@@]31[C@@H](C)[C@H](O)[C@](C)(C=C)C[C@@H](OC(CSC4=NN=C(C)N4/N=C/C5=CC=C(O)C=C5)=O)[C@]2(C)[C@H](C)CC3	6.38
O=C(CC1)[C@]2([H])[C@@]31[C@@H](C)[C@H](O)[C@](C)(C=C)C[C@@H](OC(CSC4=NN(C(C5=CC=CC(C)=C5)=O)C(N)=N4)=O)[C@]2(C)[C@H](C)CC3	6.38
O=C(CC1)[C@]2([H])[C@@]31[C@@H](C)[C@H](O)[C@](C)(C=C)C[C@@H](OC(N4C(C(C=C(N5CCC[C@H]5C(O)=O)C=C6)=C6C4)=O)=O)[C@]2(C)[C@H](C)CC3	6.37
O=C(CC1)[C@]2([H])[C@@]31[C@@H](C)[C@H](O)[C@](C)(C=C)C[C@@H](OC(CS(S(C4=NC5=C(C=CC=C5)S4)=O)=O)=O)[C@]2(C)[C@H](C)CC3	6.37
O=C(CC1)[C@]2([H])[C@@]31[C@@H](C)[C@H](O)[C@](C)(C=C)C[C@@H](OC(CSC4=CC(NC(CNCCO)=O)=CN=C4)=O)[C@]2(C)[C@H](C)CC3	6.37
O=C1CC[C@]2(CC[C@H]3C)[C@]1([H])[C@]3(C)[C@H](OC(CN4CCN(C(CNC5CCCCC5)=O)CC4)=O)C[C@@](C)(C=C)[C@@H](O)[C@@H]2C	6.37
O=C1CC[C@]2(CC[C@H]3C)[C@]1([H])[C@]3(C)[C@H](OC(CSCC(NC4=CC=C(NCC)C=C4C)=O)=O)C[C@@](C)(C=C)[C@@H](O)[C@@H]2C	6.37
O=C(CC1)[C@]2([H])[C@@]31[C@@H](C)[C@H](O)[C@](C)(C=C)C[C@@H](OC(CSC(C)(C)CNC(C4=CC=C(N)C=C4)=O)=O)[C@]2(C)[C@H](C)CC3	6.37
O=C(CC1)[C@]2([H])[C@@]31[C@@H](C)[C@H](O)[C@](C)(C=C)C[C@@H](OC(CSC(C)(C)CNC(C4=C(C)C=CC=C4)=O)=O)[C@]2(C)[C@H](C)CC3	6.37
O=C(CC1)[C@]2([H])[C@@]31[C@@H](C)[C@H](O)[C@](C)(C=C)C[C@@H](OC(CSC(C)(C)CNC(C4=CC=C(C)C=C4)=O)=O)[C@]2(C)[C@H](C)CC3	6.37
O=C(CC1)[C@]2([H])[C@@]31[C@@H](C)[C@H](O)[C@](C)(C=C)C[C@@H](OC(CSC4=NN=C(C5=CC=CC=C5[N+]([O-])=O)N4)=O)[C@]2(C)[C@H](C)CC3	6.37
O=C(CC1)[C@]2([H])[C@@]31[C@@H](C)[C@H](O)[C@](C)(C=C)C[C@@H](OC(CSC4=NN(C(C5=CC=CC=C5)=O)C(N)=N4)=O)[C@]2(C)[C@H](C)CC3	6.37
O=C(CC1)[C@]2([H])[C@@]31[C@@H](C)[C@H](O)[C@](C)(C=C)C[C@@H](OC(CSC(C)(C)CNC(CN(CC)CC)=O)=O)[C@]2(C)[C@H](C)CC3	6.36
O=C(CC1)[C@]2([H])[C@@]31[C@@H](C)[C@H](O)[C@](C)(C=C)C[C@@H](OC(CSCCNC(C4=CC=CC(Cl)=C4)=O)=O)[C@]2(C)[C@H](C)CC3	6.36
O=C(CC1)[C@]2([H])[C@@]31[C@@H](C)[C@H](O)[C@](C)(C=C)C[C@@H](OC(CSCCNC(C4=CC=C(Cl)C=C4)=O)=O)[C@]2(C)[C@H](C)CC3	6.36
O=C(CC1)[C@]2([H])[C@@]31[C@@H](C)[C@H](O)[C@](C)(C=C)C[C@@H](OC(CSC4=NN=C(C5=CC=CC(Cl)=C5)O4)=O)[C@]2(C)[C@H](C)CC3	6.36
O=C(CC1)[C@]2([H])[C@@]31[C@@H](C)[C@H](O)[C@](C)(C=C)C[C@@H](OC(CSC4=CC(NC(CN(C)C)=O)=CN=C4)=O)[C@]2(C)[C@H](C)CC3	6.36
O=C(CC1)[C@]2([H])[C@@]31[C@@H](C)[C@H](O)[C@](C)(C=C)C[C@@H](OC(CSC4=NN=C(C5=CC=C(OC)C=C5)O4)=O)[C@]2(C)[C@H](C)CC3	6.36
O=C(CC1)[C@]2([H])[C@@]31[C@@H](C)[C@H](O)[C@](C)(C=C)C[C@@H](OC(N(CC4)C(C5=C4C=C(OC)C([N+]([O-])=O)=C5)=O)=O)[C@]2(C)[C@H](C)CC3	6.36
O=C1CC[C@]2(CC[C@H]3C)[C@]1([H])[C@]3(C)[C@H](OC(CN4CCN(C5=CC=CC([N+]([O-])=O)=C5)CC4)=O)C[C@@](C)(C=C)[C@@H](O)[C@@H]2C	6.36
O=C(CC1)[C@]2([H])[C@@]31[C@@H](C)[C@H](O)[C@](C)(C=C)C[C@@H](OC(CSCCNC(C4=CC=CC(F)=C4)=O)=O)[C@]2(C)[C@H](C)CC3	6.35
O=C(CC1)[C@]2([H])[C@@]31[C@@H](C)[C@H](O)[C@](C)(C=C)C[C@@H](OC(CS(SC4=NC5=C(C=CC=C5)N4)=O)=O)[C@]2(C)[C@H](C)CC3	6.35
O=C1CC[C@]2(CC[C@H]3C)[C@]1([H])[C@]3(C)[C@H](OC(CSCC(NC4=CC=C(N)C=C4C)=O)=O)C[C@@](C)(C=C)[C@@H](O)[C@@H]2C	6.35
O=C(CC1)[C@]2([H])[C@@]31[C@@H](C)[C@H](O)[C@](C)(C=C)C[C@@H](OC(CSCCNC(C4=CC=CC(C)=C4)=O)=O)[C@]2(C)[C@H](C)CC3	6.35
O=C(CC1)[C@]2([H])[C@@]31[C@@H](C)[C@H](O)[C@](C)(C=C)C[C@@H](OC(CSC4=NN=C(C5=CC=CC=C5N)O4)=O)[C@]2(C)[C@H](C)CC3	6.35
O=C1CC[C@]2(CC[C@H]3C)[C@]1([H])[C@]3(C)[C@H](OC(CSC4=NC(OCC)=CC(C)=N4)=O)C[C@@](C)(C=C)[C@@H](O)[C@@H]2C	6.34
O=C(CC1)[C@]2([H])[C@@]31[C@@H](C)[C@H](O)[C@](C)(C=C)C[C@@H](OC(CN4CCN(C5=CC=CC(OC)=C5)CC4)=O)[C@]2(C)[C@H](C)CC3	6.34
O=C(CC1)[C@]2([H])[C@@]31[C@@H](C)[C@H](O)[C@](C)(C=C)C[C@@H](OC(CN4CCN(C5=CC=C(OC)C=C5)CC4)=O)[C@]2(C)[C@H](C)CC3	6.34
O=C1CC[C@]2(CC[C@H]3C)[C@]1([H])[C@]3(C)[C@H](OC(CN4CCN(C5=CC=CC(OC)=C5)CC4)=O)C[C@@](C)(C=C)[C@@H](O)[C@@H]2C	6.34
O=C1CC[C@]2(CC[C@H]3C)[C@]1([H])[C@]3(C)[C@H](OC(CSC4=NC(N(C)C)=CC(C)=N4)=O)C[C@@](C)(C=C)[C@@H](O)[C@@H]2C	6.34
O=C(CC1)[C@]2([H])[C@@]31[C@@H](C)[C@H](O)[C@](C)(C=C)C[C@@H](OC(CSC4=NN=C(C5=CC=CC(C)=C5)N4)=O)[C@]2(C)[C@H](C)CC3	6.34
O=C(CC1)[C@]2([H])[C@@]31[C@@H](C)[C@H](O)[C@](C)(C=C)C[C@@H](OC(CSC4=NN=C(C5=CN=CC=N5)O4)=O)[C@]2(C)[C@H](C)CC3	6.33
O=C(CC1)[C@]2([H])[C@@]31[C@@H](C)[C@H](O)[C@](C)(C=C)C[C@@H](OC(CSC4=NN=C(C5=CC=CC=N5)O4)=O)[C@]2(C)[C@H](C)CC3	6.33
O=C(CC1)[C@]2([H])[C@@]31[C@@H](C)[C@H](O)[C@](C)(C=C)C[C@@H](OC(CN4CCN(C5=CC=CC(O)=C5)CC4)=O)[C@]2(C)[C@H](C)CC3	6.33
O=C1CC[C@]2(CC[C@H]3C)[C@]1([H])[C@]3(C)[C@H](OC(CN4CCN(C5=CC=CC(O)=C5)CC4)=O)C[C@@](C)(C=C)[C@@H](O)[C@@H]2C	6.33
O=C(CC1)[C@]2([H])[C@@]31[C@@H](C)[C@H](O)[C@](C)(C=C)C[C@@H](OC(CSC4=NN=C(C5=CC=CC=C5)O4)=O)[C@]2(C)[C@H](C)CC3	6.33
O=C1CC[C@]2(CC[C@H]3C)[C@]1([H])[C@]3(C)[C@H](OC(CSC4=NC(OC)=CC(C)=N4)=O)C[C@@](C)(C=C)[C@@H](O)[C@@H]2C	6.33
O=C(CC1)[C@]2([H])[C@@]31[C@@H](C)[C@H](O)[C@](C)(C=C)C[C@@H](OC(CN4CCN(C5=CC=CC(C)=C5)CC4)=O)[C@]2(C)[C@H](C)CC3	6.33
O=C(CC1)[C@]2([H])[C@@]31[C@@H](C)[C@H](O)[C@](C)(C=C)C[C@@H](OC(CN4N=NC(C5=CC=C(CO)C=C5)=C4)=O)[C@]2(C)[C@H](C)CC3	6.33
O=C(CC1)[C@]2([H])[C@@]31[C@@H](C)[C@H](O)[C@](C)(C=C)C[C@@H](OC(CSC4=CC(/C=N\OC)=CN=C4)=O)[C@]2(C)[C@H](C)CC3	6.33
O=C(CC1)[C@]2([H])[C@@]31[C@@H](C)[C@H](O)[C@](C)(C=C)C[C@@H](OC(CNC45CC(CC(C)C5)CC(C)C4)=O)[C@]2(C)[C@H](C)CC3	6.32
O=C(CC1)[C@]2([H])[C@@]31[C@@H](C)[C@H](O)[C@](C)(C=C)C[C@@H](OC(CSC4=NC(C=CC=C5)=C5S4)=O)[C@]2(C)[C@H](C)CC3	6.32
O=C(CC1)[C@]2([H])[C@@]31[C@@H](C)[C@H](O)[C@](C)(C=C)C[C@@H](OC(CSC4=NC(C=CC=C5)=C5S4)=O)[C@]2(C)[C@H](C)CC3	6.32
O=C(CC1)[C@]2([H])[C@@]31[C@@H](C)[C@H](O)[C@](C)(C=C)C[C@@H](OC(CS(SC4=NN=CS4)=O)=O)[C@]2(C)[C@H](C)CC3	6.32
O=C(CC1)[C@]2([H])[C@@]31[C@@H](C)[C@H](O)[C@](C)(C=C)C[C@@H](OC(CSC4=NC(C=CC=C5)=C5N4)=O)[C@]2(C)[C@H](C)CC3	6.31
O=C(CC1)[C@]2([H])[C@@]31[C@@H](C)[C@H](O)[C@](C)(C=C)C[C@@H](OC(CSC4=NC(C=CC=C5)=C5N4)=O)[C@]2(C)[C@H](C)CC3	6.31
O=C(CC1)[C@]2([H])[C@@]31[C@@H](C)[C@H](O)[C@](C)(C=C)C[C@@H](OC(CSCC4=CSC(N)=N4)=O)[C@]2(C)[C@H](C)CC3	6.31
O=C(CC1)[C@]2([H])[C@@]31[C@@H](C)[C@H](O)[C@](C)(C=C)C[C@@H](OC(CSCC4=CSC(N)=N4)=O)[C@]2(C)[C@H](C)CC3	6.31
O=C1CC[C@]2(CC[C@H]3C)[C@]1([H])[C@]3(C)[C@H](OC(CSC4=NC(O)=CC(N)=N4)=O)C[C@@](C)(C=C)[C@@H](O)[C@@H]2C	6.30
O=C1CC[C@]2(CC[C@H]3C)[C@]1([H])[C@]3(C)[C@H](OC(CSC4=NC(O)=CC(C)=N4)=O)C[C@@](C)(C=C)[C@@H](O)[C@@H]2C	6.30
O=C(CC1)[C@]2([H])[C@@]31[C@@H](C)[C@H](O)[C@](C)(C=C)C[C@@H](OC(CSC[C@H](O4)CNC4=O)=O)[C@]2(C)[C@H](C)CC3	6.30
O=C(CC1)[C@]2([H])[C@@]31[C@@H](C)[C@H](O)[C@](C)(C=C)C[C@@H](OC(CSC[C@H](O4)CNC4=O)=O)[C@]2(C)[C@H](C)CC3	6.30

O=C(CC1)[C@]2([H])[C@@]31[C@@H](C)[C@H](O)[C@](C)(C=C)C[C@@H](OC(N(CC4)C(C5=C4C=CC=C5)=O)=O)[C@]2(C)[C@H](C)CC3	6.30
O=C(CC1)[C@]2([H])[C@@]31[C@@H](C)[C@H](O)[C@](C)(C=C)C[C@@H](OC(CNCCC4=CC=CS4)=O)[C@]2(C)[C@H](C)CC3	6.29
O=C(CC1)[C@]2([H])[C@@]31[C@@H](C)[C@H](O)[C@](C)(C=C)C[C@@H](OC(CSCC4=C(O)C(C=C(CO)O4)=O)=O)[C@]2(C)[C@H](C)CC3	6.29
O=C(CC1)[C@]2([H])[C@@]31[C@@H](C)[C@H](O)[C@](C)(C=C)C[C@@H](OC(C[N+]4=CC=C(N(C)C)C=C4)=O)[C@]2(C)[C@H](C)CC3	6.29
O=C(CC1)[C@]2([H])[C@@]31[C@@H](C)[C@H](O)[C@](C)(C=C)C[C@@H](OC(CN4CCCC(CO)C4)=O)[C@]2(C)[C@H](C)CC3	6.28
O=C(CC1)[C@]2([H])[C@@]31[C@@H](C)[C@H](O)[C@](C)(C=C)C[C@@H](OC(CSC4=NN=CC=C4)=O)[C@]2(C)[C@H](C)CC3	6.28
O=C(CC1)[C@]2([H])[C@@]31[C@@H](C)[C@H](O)[C@](C)(C=C)C[C@@H](OC(CSC4CCN(C(CCN5C=NC6=C5N=C(N)N=C6N7CCCC7)=O)CC4)=O)[C@]2(C)[C@H](C)CC3	6.28
O=C(CC1)[C@]2([H])[C@@]31[C@@H](C)[C@H](O)[C@](C)(C=C)C[C@@H](OC(CSC4=C(NC(CN5N=NC(CN6CCN(C7=CC=CC=C7[N+]([O-])=O)CC6)=C5)=O)C=CC=C4)=O)[C@]2(C)[C@H](C)CC3	6.21
O=C(CC1)[C@]2([H])[C@@]31[C@@H](C)[C@H](O)[C@](C)(C=C)C[C@@H](OC(C[N+]4(CCC(O)C4)C)=O)[C@]2(C)[C@H](C)CC3	6.27
O=C(CC1)[C@]2([H])[C@@]31[C@@H](C)[C@H](O)[C@](C)(C=C)C[C@@H](OC(CSCC4=CC(C(O)=C(CN(C)C)O4)=O)=O)[C@]2(C)[C@H](C)CC3	6.27
O=C(CC1)[C@]2([H])[C@@]31[C@@H](C)[C@H](O)[C@](C)(C=C)C[C@@H](OC(CSC4=C(NC(CN5N=NC(CN6CCN(C7=CC=C(Cl)C=C7)CC6)=C5)=O)C=CC=C4)=O)[C@]2(C)[C@H](C)CC3	6.21
O=C(CC1)[C@]2([H])[C@@]31[C@@H](C)[C@H](O)[C@](C)(C=C)C[C@@H](OC(CSC4=C(NC(CN5N=NC(CN6CCN(C7=CC=CC=C7OC)CC6)=C5)=O)C=CC=C4)=O)[C@]2(C)[C@H](C)CC3	6.20
O=C(CC1)[C@]2([H])[C@@]31[C@@H](C)[C@H](O)[C@](C)(C=C)C[C@@H](OC(CN4CCC(O)C4)=O)[C@]2(C)[C@H](C)CC3	6.25
O=C(CC1)[C@]2([H])[C@@]31[C@@H](C)[C@H](O)[C@](C)(C=C)C[C@@H](OC(C[N+]4(CCCC4)C)=O)[C@]2(C)[C@H](C)CC3	6.25
O=C(CC1)[C@]2([H])[C@@]31[C@@H](C)[C@H](O)[C@](C)(C=C)C[C@@H](OC(CSC4=C(NC(CN5N=NC(CN6CCN(C7=CC=CC=C7O)CC6)=C5)=O)C=CC=C4)=O)[C@]2(C)[C@H](C)CC3	6.20
O=C(CC1)[C@]2([H])[C@@]31[C@@H](C)[C@H](O)[C@](C)(C=C)C[C@@H](OC(CSC4CCN(C(CCN5C=NC6=C5N=CN=C6N7CC(CNC)CC7)=O)CC4)=O)[C@]2(C)[C@H](C)CC3	6.19
O=C(CC1)[C@]2([H])[C@@]31[C@@H](C)[C@H](O)[C@](C)(C=C)C[C@@H](OC(CSC4=C(NC(CN5N=NC(CN6CCN(C7=CC=CC=C7)CC6)=C5)=O)C=CC=C4)=O)[C@]2(C)[C@H](C)CC3	6.19
O=C(CC1)[C@]2([H])[C@@]31[C@@H](C)[C@H](O)[C@](C)(CC)C[C@@H](OC(COC(CC4)CCN4C(CCN5C=NC6=C5N=CN=C6N7CCC(C7)N)=O)=O)[C@]2(C)[C@H](C)CC3	6.19
O=C(CC1)[C@]2([H])[C@@]31[C@@H](C)[C@H](O)[C@](C)(C=C)C[C@@H](OC(CS[C@H]4CC[C@H](C(OCC)=O)C[C@H]4NS(=O)(C5=CC=C(NC(C)=O)C=C5)=O)=O)[C@]2(C)[C@H](C)CC3	6.18
O=C(CC1)[C@]2([H])[C@@]31[C@@H](C)[C@H](O)[C@](C)(C=C)C[C@@H](OC(CSC4CCN(C(CCN5C=NC6=C5N=C(N)N=C6N7CCNCC7)=O)CC4)=O)[C@]2(C)[C@H](C)CC3	6.18
O=C(CC1)[C@]2([H])[C@@]31[C@@H](C)[C@H](O)[C@](C)(C=C)C[C@@H](OC(CSC4CCN(C(CCN5C=NC6=C5N=C(N)N=C6N7CCNCC7)=O)CC4)=O)[C@]2(C)[C@H](C)CC3	6.18
O=C(CC1)[C@]2([H])[C@@]31[C@@H](C)[C@H](O)[C@](C)(C=C)C[C@@H](OC(CSC4CCN(C(CCN5C=NC6=C5N=CN=C6N7CC(CN)CC7)=O)CC4)=O)[C@]2(C)[C@H](C)CC3	6.18
O=C(CC1)[C@]2([H])[C@@]31[C@@H](C)[C@H](O)[C@](C)(C=C)C[C@@H](OC(CS[C@H]4CC[C@H](C(OCC)=O)C[C@H]4NS(=O)(C5=CC=C(Cl)C=C5)=O)=O)[C@]2(C)[C@H](C)CC3	6.17
O=C(CC1)[C@]2([H])[C@@]31[C@@H](C)[C@H](O)[C@](C)(C=C)C[C@@H](OC(CSCC(C4)CN4C(CCN5C=NC6=C5N=C(N)N=C6N7CCNCC7)=O)=O)[C@]2(C)[C@H](C)CC3	6.17
O=C(CC1)[C@]2([H])[C@@]31[C@@H](C)[C@H](O)[C@](C)(C=C)C[C@@H](OC(CSC4CCCN(C(CCN5C=NC6=C5N=CN=C6N7CCC(C7)N)=O)C4)=O)[C@]2(C)[C@H](C)CC3	6.17
O=C(CC1)[C@]2([H])[C@@]31[C@@H](C)[C@H](O)[C@](C)(C=C)C[C@@H](OC(CSC4CCN(C(CCN5C=NC6=C5N=CN=C6N7C[C@@H](N)CC7)=O)CC4)=O)[C@]2(C)[C@H](C)CC3	6.17
O=C(CC1)[C@]2([H])[C@@]31[C@@H](C)[C@H](O)[C@](C)(C=C)C[C@@H](OC(CSC4=C(NC(CN5N=NC(CN6CCC(CCO)CC6)=C5)=O)C=CC=C4)=O)[C@]2(C)[C@H](C)CC3	6.17
O=C(CC1)[C@]2([H])[C@@]31[C@@H](C)[C@H](O)[C@](C)(C=C)C[C@@H](OC(CN4N=NC(C5=CC=CC(CN6C7=NC=NC(N8CCN(C)CC8)=C7N=C6)=C5)=C4)=O)[C@]2(C)[C@H](C)CC3	6.17
O=C1CC[C@]2(CC[C@H]3C)[C@]1([H])[C@]3(C)[C@H](OC(CSC4=NC(NC=C5)=C5C(NC(CN6CCC(N7CCCCC7)CC6)=O)=N4)=O)C[C@@](C)(C=C)[C@@H](O)[C@@H]2C	6.17
O=C1CC[C@]2(CC[C@H]3C)[C@]1([H])[C@]3(C)[C@H](OC(CSC4=NC(NC=C5)=C5C(NC(CN6CCN(C7=CC=CC=N7)CC6)=O)=N4)=O)C[C@@](C)(C=C)[C@@H](O)[C@@H]2C	6.16
O=C(CC1)[C@]2([H])[C@@]31[C@@H](C)[C@H](O)[C@](C)(C=C)C[C@@H](OC(CN4N=NC(C5=CC=CC=C5CN6C7=NC(N)=NC(N(CC)CC)=C7N=C6)=C4)=O)[C@]2(C)[C@H](C)CC3	6.16
O=C(CC1)[C@]2([H])[C@@]31[C@@H](C)[C@H](O)[C@](C)(C=C)C[C@@H](OC(CN4N=NC(C5=CC=CC(CN6C7=NC(N)=NC(N(CC)CC)=C7N=C6)=C5)=C4)=O)[C@]2(C)[C@H](C)CC3	6.16
O=C(CC1)[C@]2([H])[C@@]31[C@@H](C)[C@H](O)[C@](C)(C=C)C[C@@H](OC(CSC4=C(NC(CN5N=NC(CN6CCCC(CO)C6)=C5)=O)C=CC=C4)=O)[C@]2(C)[C@H](C)CC3	6.16
O=C(CC1)[C@]2([H])[C@@]31[C@@H](C)[C@H](O)[C@](C)(C=C)C[C@@H](OC(CSCCN)=O)[C@]2(C)[C@H](C)CC3	6.24
O=C(CC1)[C@]2([H])[C@@]31[C@@H](C)[C@H](O)[C@](C)(C=C)C[C@@H](OC(CSC4CCN(C(CCN5C=NC6=C5N=CN=C6N)=O)CC4)=O)[C@]2(C)[C@H](C)CC3	6.23
O=C(CC1)[C@]2([H])[C@@]31[C@@H](C)[C@H](O)[C@](C)(C=C)C[C@@H](OC(CSCCN(C(CCN4C=NC5=C4N=CN=C5N6CCC(N)C6)=O)C)=O)[C@]2(C)[C@H](C)CC3	6.15
O=C(CC1)[C@]2([H])[C@@]31[C@@H](C)[C@H](O)[C@](C)(C=C)C[C@@H](OC(CS[C@H]4CC[C@H](C(OCC)=O)C[C@H]4NS(=O)(C5=CC=CC=C5)=O)=O)[C@]2(C)[C@H](C)CC3	6.15
O=C(CC1)[C@]2([H])[C@@]31[C@@H](C)[C@H](O)[C@](C)(C=C)C[C@@H](OC(CN4CCN(C5=CC=C(C(/C=C/C6=CC=C([N+]([O-])=O)C=C6)=O)C=C5)CC4)=O)[C@]2(C)[C@H](C)CC3	6.14
O=C(CC1)[C@]2([H])[C@@]31[C@@H](C)[C@H](O)[C@](C)(C=C)C[C@@H](OC(CN4N=NC(C5=CC=C(CN6C7=NC=NC(NCCN)=C7N=C6)C=C5)=C4)=O)[C@]2(C)[C@H](C)CC3	6.14
O=C(CC1)[C@]2([H])[C@@]31[C@@H](C)[C@H](O)[C@](C)(C=C)C[C@@H](OC(CN4N=NC(C5=CC=C(CN6C7=NC=NC(NC(N)=N)=C7N=C6)C=C5)=C4)=O)[C@]2(C)[C@H](C)CC3	6.14
O=C(CC1)[C@]2([H])[C@@]31[C@@H](C)[C@H](O)[C@](C)(C=C)C[C@@H](OC(CSC4=C(NC(CN5N=NC(CN6CCOCC6)=C5)=O)C=CC=C4)=O)[C@]2(C)[C@H](C)CC3	6.14
O=C(CC1)[C@]2([H])[C@@]31[C@@H](C)[C@H](O)[C@](C)(C=C)C[C@@H](OC(C[N+](C)(C)C)=O)[C@]2(C)[C@H](C)CC3	6.23

O=C(CC1)[C@]2([H])[C@@]31[C@@H](C)[C@H](O)[C@](C)(C=C)C[C@@H](OC(CSC4=C(NC(CN5N=NC(CN6CCCCC6)=C5)=O)C=CC=C4)=O)[C@]2(C)[C@H](C)CC3	6.14
O=C(CC1)[C@]2([H])[C@@]31[C@@H](C)[C@H](O)[C@](C)(C=C)C[C@@H](OC(CSC4=CC(C=NO)=CN=C4)=O)[C@]2(C)[C@H](C)CC3	6.22
O=C(CC1)[C@]2([H])[C@@]31[C@@H](C)[C@H](O)[C@](C)(C=C)C[C@@H](OC(CN(C)C)=O)[C@]2(C)[C@H](C)CC3	6.21
O=C(CC1)[C@]2([H])[C@@]31[C@@H](C)[C@H](O)[C@](C)(C=C)C[C@@H](OC(CN4CCN(C(CN5CCN(C6=CC=CC=C6Cl)CC5)=O)CC4)=O)[C@]2(C)[C@H](C)CC3	6.14
CC1C23C(C(CC3)=O)C(C(CC(C=C)(C)C1O)OC(CN4CCN(C(CN5CCN(C6=C(Cl)C=CC=C6)CC5)=O)CC4)=O)(C(CC2)C)C	6.14
O=C(CC1)[C@]2([H])[C@@]31[C@@H](C)[C@H](O)[C@](C)(C=C)C[C@@H](OC(CN4CCN(C(/C=C/C5=C(OC)C=CC=C5)=O)CC4)=O)[C@]2(C)[C@H](C)CC3	6.21
O=C(CC1)[C@]2([H])[C@@]31[C@@H](C)[C@H](O)[C@](C)(C=C)C[C@@H](OC(CSCC4=CSC(NC(C5=CC=C(Cl)C=C5Cl)=O)=N4)=O)[C@]2(C)[C@H](C)CC3	6.13
O=C(CC1)[C@]2([H])[C@@]31[C@@H](C)[C@H](O)[C@](C)(C=C)C[C@@H](OC(CN4CCN(C(CN5CCN(C6=CC=C(OC)C=C6)CC5)=O)CC4)=O)[C@]2(C)[C@H](C)CC3	6.13
CC1C23C(C(CC3)=O)C(C(CC(C=C)(C)C1O)OC(CN4CCN(C(CN5CCN(C6=C(OC)C=CC=C6)CC5)=O)CC4)=O)(C(CC2)C)C	6.13
CC1C23C(C(CC3)=O)C(C(CC(C=C)(C)C1O)OC(CN4CCN(C(CN5CCN(C6=CC=C(OC)C=C6)CC5)=O)CC4)=O)(C(CC2)C)C	6.13
O=C1CC[C@]2(CC[C@H]3C)[C@]1([H])[C@]3(C)[C@H](OC(CN4CCN(C([C@H](CCSC)NC(OC(C)(C)C)=O)=O)CC4)=O)C[C@@](C)(C=C)[C@@H](O)[C@@H]2C	6.13
O=C(CC1)[C@]2([H])[C@@]31[C@@H](C)[C@H](O)[C@](C)(C=C)C[C@@H](OC(CN4CCN(C5=CC=C(C(/C=C/C6=CC=C(N)C=C6)=O)C=C5)CC4)=O)[C@]2(C)[C@H](C)CC3	6.13
O=C(CC1)[C@]2([H])[C@@]31[C@@H](C)[C@H](O)[C@](C)(C=C)C[C@@H](OC(N4CC5=C(C=CC=C5OCCN6C=NC(C7=CC=CN=C7)=C6)C4=O)=O)[C@]2(C)[C@H](C)CC3	6.13
CC1C23C(C(CC3)=O)C(C(CC(C=C)(C)C1O)OC(CN4CCN(C(CN5CCN(C6=CC(O)=CC=C6)CC5)=O)CC4)=O)(C(CC2)C)C	6.12
O=C(CC1)[C@]2([H])[C@@]31[C@@H](C)[C@H](O)[C@](C)(C=C)C[C@@H](OC(N4C(C(C=C(NC(CN5CCN(CCO)CC5)=O)C=C6)=C6C4)=O)=O)[C@]2(C)[C@H](C)CC3	6.12
O=C(CC1)[C@]2([H])[C@@]31[C@@H](C)[C@H](O)[C@](C)(C=C)C[C@@H](OC(CSC4=NN=C(C)N4/N=C/C5=CC=C(N6CCOCC6)C=C5)=O)[C@]2(C)[C@H](C)CC3	6.12
O=C(CC1)[C@]2([H])[C@@]31[C@@H](C)[C@H](O)[C@](C)(C=C)C[C@@H](OC(CN4CCN(C(CN5CCN(C6=CC=CC=C6C)CC5)=O)CC4)=O)[C@]2(C)[C@H](C)CC3	6.12
O=C(CC1)[C@]2([H])[C@@]31[C@@H](C)[C@H](O)[C@](C)(C=C)C[C@@H](OC(CN4CCN(C(CN5CCN(C6=CC=CC(C)=C6)CC5)=O)CC4)=O)[C@]2(C)[C@H](C)CC3	6.12
CC1C23C(C(CC3)=O)C(C(CC(C=C)(C)C1O)OC(CN4CCN(C(CN5CCN(C6=CC(C)=CC=C6)CC5)=O)CC4)=O)(C(CC2)C)C	6.12
O=C(CC1)[C@]2([H])[C@@]31[C@@H](C)[C@H](O)[C@](C)(C=C)C[C@@H](OC(CN4CCN(C(/C=C/C5=CC=CC=C5F)=O)CC4)=O)[C@]2(C)[C@H](C)CC3	6.20
O=C(CC1)[C@]2([H])[C@@]31[C@@H](C)[C@H](O)[C@](C)(C=C)C[C@@H](OC(CSCC4=CSC(NC([C@@]([H])(N)CC5=CC=CC=C5)=O)=N4)=O)[C@]2(C)[C@H](C)CC3	6.12
O=C(CC1)[C@]2([H])[C@@]31[C@@H](C)[C@H](O)[C@](C)(C=C)C[C@@H](OC(CN4CCN(C(/C=C/C5=CC=C(F)C=C5)=O)CC4)=O)[C@]2(C)[C@H](C)CC3	6.20
O=C(CC1)[C@]2([H])[C@@]31[C@@H](C)[C@H](O)[C@](C)(C=C)C[C@@H](OC(CN4N=NC(C5=CC=CC=C5CN6C7=NC=NC(N)=C7N=C6)=C4)=O)[C@]2(C)[C@H](C)CC3	6.12
O=C(CC1)[C@]2([H])[C@@]31[C@@H](C)[C@H](O)[C@](C)(C=C)C[C@@H](OC(N(CC4)C(C5=C4C=C(OCCCCN6CCOCC6)C=C5)=O)=O)[C@]2(C)[C@H](C)CC3	6.11
O=C(CC1)[C@]2([H])[C@@]31[C@@H](C)[C@H](O)[C@](C)(C=C)C[C@@H](OC(N(CC4)C(C5=C4C=C(OCCCN6CCN(C)CC6)C=C5)=O)=O)[C@]2(C)[C@H](C)CC3	6.11
O=C(CC1)[C@]2([H])[C@@]31[C@@H](C)[C@H](O)[C@](C)(C=C)C[C@@H](OC(CN4CCN(C(CN5CCN(C6=CC=CC=C6)CC5)=O)CC4)=O)[C@]2(C)[C@H](C)CC3	6.11
O=C(CC1)[C@]2([H])[C@@]31[C@@H](C)[C@H](O)[C@](C)(C=C)C[C@@H](OC(N4C(C(C=C(NC(CCN5CCN(C)CC5)=O)C=C6)=C6C4)=O)=O)[C@]2(C)[C@H](C)CC3	6.11
O=C(CC1)[C@]2([H])[C@@]31[C@@H](C)[C@H](O)[C@](C)(C=C)C[C@@H](OC(CN4N=NC(CN5CCN(C6=CC=C([N+]([O-])=O)C=C6)CC5)=C4)=O)[C@]2(C)[C@H](C)CC3	6.11
O=C(CC1)[C@]2([H])[C@@]31[C@@H](C)[C@H](O)[C@](C)(C=C)C[C@@H](OC(CSCC4=CSC(NC(CN(CCC)CCC)=O)=N4)=O)[C@]2(C)[C@H](C)CC3	6.11
O=C1CC[C@]2(CC[C@H]3C)[C@]1([H])[C@]3(C)[C@H](OC(CN4CCN(C(NCC5=CC=C(C(F)(F)F)C=C5)=O)CC4)=O)C[C@@](C)(C=C)[C@@H](O)[C@@H]2C	6.11
O=C(CC1)[C@]2([H])[C@@]31[C@@H](C)[C@H](O)[C@](C)(C=C)C[C@@H](OC(CSCC4=CSC(NC(CN5CCN(C)CC5)=O)=N4)=O)[C@]2(C)[C@H](C)CC3	6.11
O=C(CC1)[C@]2([H])[C@@]31[C@@H](C)[C@H](O)[C@](C)(C=C)C[C@@H](OC(CSCC4=CSC(NC(CNC5CCCCC5)=O)=N4)=O)[C@]2(C)[C@H](C)CC3	6.11
O=C1CC[C@]2(CC[C@H]3C)[C@]1([H])[C@]3(C)[C@H](OC(CSC4=NC(N5CCC(CO)CC5)=CC(C)=N4)=O)C[C@@](C)(C=C)[C@@H](O)[C@@H]2C	6.15
O=C(CC1)[C@]2([H])[C@@]31[C@@H](C)[C@H](O)[C@](C)(C=C)C[C@@H](OC(CN4N=NC(C5=CC=C(N6C=NC7=C6N=CN=C7N)C=C5)=C4)=O)[C@]2(C)[C@H](C)CC3	6.11
O=C(CC1)[C@]2([H])[C@@]31[C@@H](C)[C@H](O)[C@](C)(C=C)C[C@@H](OC(CN4N=NC(C5=CC=C(N6C7=NC=NC(N)=C7N=C6)C=C5)=C4)=O)[C@]2(C)[C@H](C)CC3	6.11
O=C(CC1)[C@]2([H])[C@@]31[C@@H](C)[C@H](O)[C@](C)(C=C)C[C@@H](OC(N(CC4)C(C5=C4C=C(OCCCN6CCOCC6)C=C5)=O)=O)[C@]2(C)[C@H](C)CC3	6.11
O=C(CC1)[C@]2([H])[C@@]31[C@@H](C)[C@H](O)[C@](C)(C=C)C[C@@H](OC(N(CC4)C(C5=C4C=C(OCCN6CCN(C)CC6)C=C5)=O)=O)[C@]2(C)[C@H](C)CC3	6.10
O=C(CC1)[C@]2([H])[C@@]31[C@@H](C)[C@H](O)[C@](C)(C=C)C[C@@H](OC(N4C(C(C=C(NC(CCN5CCOCC5)=O)C=C6)=C6C4)=O)=O)[C@]2(C)[C@H](C)CC3	6.10
O=C(CC1)[C@]2([H])[C@@]31[C@@H](C)[C@H](O)[C@](C)(C=C)C[C@@H](OC(N(CC4)C(C5=C4C=C(OCCCN6CCCCC6)C=C5)=O)=O)[C@]2(C)[C@H](C)CC3	6.10
O=C(CC1)[C@]2([H])[C@@]31[C@@H](C)[C@H](O)[C@](C)(C=C)C[C@@H](OC(N4C(C(C=C(NC(CN5CCN(C)CC5)=O)C=C6)=C6C4)=O)=O)[C@]2(C)[C@H](C)CC3	6.10
O=C(CC1)[C@]2([H])[C@@]31[C@@H](C)[C@H](O)[C@](C)(C=C)C[C@@H](OC(CSCC4=CSC(NC(CN5CCOCC5)=O)=N4)=O)[C@]2(C)[C@H](C)CC3	6.10
O=C(CC1)[C@]2([H])[C@@]31[C@@H](C)[C@H](O)[C@](C)(C=C)C[C@@H](OC(CSCC4=CSC(NC(CN5CCCCC5)=O)=N4)=O)[C@]2(C)[C@H](C)CC3	6.10
O=C(CC1)[C@]2([H])[C@@]31[C@@H](C)[C@H](O)[C@](C)(C=C)C[C@@H](OC(CSCC4=CSC(NC(CN5CCCCC5)=O)=N4)=O)[C@]2(C)[C@H](C)CC3	6.10
O=C(CC1)[C@]2([H])[C@@]31[C@@H](C)[C@H](O)[C@](C)(C=C)C[C@@H](OC(CSCC4=CSC(NC(C5=CC=CC=C5O)=O)=N4)=O)[C@]2(C)[C@H](C)CC3	6.10
O=C(CC1)[C@]2([H])[C@@]31[C@@H](C)[C@H](O)[C@](C)(C=C)C[C@@H](OC(CSC4=C(NC(CN5CCN(C)CC5)=O)C=CC=C4)=O)[C@]2(C)[C@H](C)CC3	6.10
O=C(CC1)[C@]2([H])[C@@]31[C@@H](C)[C@H](O)[C@](C)(C=C)C[C@@H](OC(CSC4=NN=C(NC([C@H](C)C5=CC=CC=C5)=O)S4)=O)[C@]2(C)[C@H](C)CC3	6.10
O=C(CC1)[C@]2([H])[C@@]31[C@@H](C)[C@H](O)[C@](C)(C=C)C[C@@H](OC(CN4CCN(C(NCC5=CC=C([N+]([O-])=O)C=C5)=O)CC4)=O)[C@]2(C)[C@H](C)CC3	6.10
O=C(CC1)[C@]2([H])[C@@]31[C@@H](C)[C@H](O)[C@](C)(C=C)C[C@@H](OC(CSC4=C(NC(C5=C(Cl)C=CC=C5)=O)C=CC=C4)=O)[C@]2(C)[C@H](C)CC3	6.10
O=C(CC1)[C@]2([H])[C@@]31[C@@H](C)[C@H](O)[C@](C)(C=C)C[C@@H](OC(CSC4=C(NC(C5=CC=C(Cl)C=C5)=O)C=CC=C4)=O)[C@]2(C)[C@H](C)CC3	6.10
O=C(CC1)[C@]2([H])[C@@]31[C@@H](C)[C@H](O)[C@](C)(C=C)C[C@@H](OC(N(CC4)C(C5=C4C=C(OCCN6CCOCC6)C=C5)=O)=O)[C@]2(C)[C@H](C)CC3	6.10
O=C(CC1)[C@]2([H])[C@@]31[C@@H](C)[C@H](O)[C@](C)(C=C)C[C@@H](OC(CSC4=NN=C(C)N4/N=C/C5=CC=C(N(C)C)C=C5)=O)[C@]2(C)[C@H](C)CC3	6.09
O=C(CC1)[C@]2([H])[C@@]31[C@@H](C)[C@H](O)[C@](C)(C=C)C[C@@H](OC(N4C(C(C=C(NC(CN5CCOCC5)=O)C=C6)=C6C4)=O)=O)[C@]2(C)[C@H](C)CC3	6.09
O=C(CC1)[C@]2([H])[C@@]31[C@@H](C)[C@H](O)[C@](C)(C=C)C[C@@H](OC(N(CC4)C(C5=C4C=C(OCCN6CCCCC6)C=C5)=O)=O)[C@]2(C)[C@H](C)CC3	6.09
O=C(CC1)[C@]2([H])[C@@]31[C@@H](C)[C@H](O)[C@](C)(C=C)C[C@@H](OC(CSCC4=CSC(NC(CN(CC)CC)=O)=N4)=O)[C@]2(C)[C@H](C)CC3	6.09
O=C(CC1)[C@]2([H])[C@@]31[C@@H](C)[C@H](O)[C@](C)(C=C)C[C@@H](OC(CSCC4=CC(C(OC)=CO4)=O)=O)[C@]2(C)[C@H](C)CC3	6.15

O=C1CC[C@]2(CC[C@H]3C)[C@]1([H])[C@]3(C)[C@H](OC(CSC4=NC(N)=CC(NC(CN(CC)CC)=O)=N4)=O)C[C@@](C)(C=C)[C@@H](O)[C@@H]2C	6.09
O=C(CC1)[C@]2([H])[C@@]31[C@@H](C)[C@H](O)[C@](C)(C=C)C[C@@H](OC(CSC4=NC(NC(CN(CC)CC)=O)=CC(N)=N4)=O)[C@]2(C)[C@H](C)CC3	6.09
O=C(CC1)[C@]2([H])[C@@]31[C@@H](C)[C@H](O)[C@](C)(C=C)C[C@@H](OC(CSC4=NN(C(C5=CC=CC=C5Cl)=O)C(N)=N4)=O)[C@]2(C)[C@H](C)CC3	6.09
O=C(CC1)[C@]2([H])[C@@]31[C@@H](C)[C@H](O)[C@](C)(C=C)C[C@@H](OC(CSC4=NN(C(C5=CC=C(Cl)C=C5)=O)C(N)=N4)=O)[C@]2(C)[C@H](C)CC3	6.09
O=C1CC[C@]2(CC[C@H]3C)[C@]1([H])[C@]3(C)[C@H](OC(CSC4=NC(N)=CC(NC(CN5CCCC5)=O)=N4)=O)C[C@@](C)(C=C)[C@@H](O)[C@@H]2C	6.09
O=C(CC1)[C@]2([H])[C@@]31[C@@H](C)[C@H](O)[C@](C)(C=C)C[C@@H](OC(CSC4=NC(NC(CN5CCCC5)=O)=CC(N)=N4)=O)[C@]2(C)[C@H](C)CC3	6.09
O=C(CC1)[C@]2([H])[C@@]31[C@@H](C)[C@H](O)[C@](C)(C=C)C[C@@H](OC(CSC4=NN=C(C)N4/N=C/C5=CC=C(Cl)C=C5)=O)[C@]2(C)[C@H](C)CC3	6.09
O=C1CC[C@]2(CC[C@H]3C)[C@]1([H])[C@]3(C)[C@H](OC(CSCC(NC4=CC=C(NCCCC)C=C4C)=O)=O)C[C@@](C)(C=C)[C@@H](O)[C@@H]2C	6.09
O=C(CC1)[C@]2([H])[C@@]31[C@@H](C)[C@H](O)[C@](C)(C=C)C[C@@H](OC(CN4CCN(C(NC5=CC=C([N+]([O-])=O)C=C5)=O)CC4)=O)[C@]2(C)[C@H](C)CC3	6.09
O=C(CC1)[C@]2([H])[C@@]31[C@@H](C)[C@H](O)[C@](C)(C=C)C[C@@H](OC(CS(SC4=NC5=C(C=CC(Cl)=C5)S4)=O)=O)[C@]2(C)[C@H](C)CC3	6.09
O=C(CC1)[C@]2([H])[C@@]31[C@@H](C)[C@H](O)[C@](C)(C=C)C[C@@H](OC(CN4CCN(C(NCC5=CC=C(OC)C=C5)=O)CC4)=O)[C@]2(C)[C@H](C)CC3	6.09
O=C(CC1)[C@]2([H])[C@@]31[C@@H](C)[C@H](O)[C@](C)(C=C)C[C@@H](OC(CSC4=C(NC(C5=C(F)C=CC=C5)=O)C=CC=C4)=O)[C@]2(C)[C@H](C)CC3	6.08
O=C(CC1)[C@]2([H])[C@@]31[C@@H](C)[C@H](O)[C@](C)(C=C)C[C@@H](OC(CSC4=NN=C(C5=CC=C(C(F)(F)F)C=C5)N4)=O)[C@]2(C)[C@H](C)CC3	6.08
O=C(CC1)[C@]2([H])[C@@]31[C@@H](C)[C@H](O)[C@](C)(C=C)C[C@@H](OC(CSC4=NN=C(NC(N5CCCCC5)=O)S4)=O)[C@]2(C)[C@H](C)CC3	6.08
O=C(CC1)[C@]2([H])[C@@]31[C@@H](C)[C@H](O)[C@](C)(C=C)C[C@@H](OC(CSC(C)(C)CNC(C4=C(Cl)C=CC=C4)=O)=O)[C@]2(C)[C@H](C)CC3	6.08
O=C(CC1)[C@]2([H])[C@@]31[C@@H](C)[C@H](O)[C@](C)(C=C)C[C@@H](OC(CSCC4=CSC(NC(C5CCCN5)=O)=N4)=O)[C@]2(C)[C@H](C)CC3	6.08
O=C(CC1)[C@]2([H])[C@@]31[C@@H](C)[C@H](O)[C@](C)(C=C)C[C@@H](OC(CSC4=C(NC(C5=CC=C(C)C=C5)=O)C=CC=C4)=O)[C@]2(C)[C@H](C)CC3	6.08
O=C(CC1)[C@]2([H])[C@@]31[C@@H](C)[C@H](O)[C@](C)(C=C)C[C@@H](OC(CSC4=NN(C(C5=CC=C(F)C=C5)=O)C(N)=N4)=O)[C@]2(C)[C@H](C)CC3	6.08
O=C1CC[C@]2(CC[C@H]3C)[C@]1([H])[C@]3(C)[C@H](OC(CSCC(NC4=CC=C(NCC=C)C=C4C)=O)=O)C[C@@](C)(C=C)[C@@H](O)[C@@H]2C	6.08
O=C(CC1)[C@]2([H])[C@@]31[C@@H](C)[C@H](O)[C@](C)(C=C)C[C@@H](OC(CSC4=NN=C(C)N4/N=C/C5=CC=CC=C5F)=O)[C@]2(C)[C@H](C)CC3	6.08
O=C(CC1)[C@]2([H])[C@@]31[C@@H](C)[C@H](O)[C@](C)(C=C)C[C@@H](OC(CSC4=NN=C(C)N4/N=C/C5=CC=CC(F)=C5)=O)[C@]2(C)[C@H](C)CC3	6.08
O=C(CC1)[C@]2([H])[C@@]31[C@@H](C)[C@H](O)[C@](C)(C=C)C[C@@H](OC(CSC4=NN=C(C)N4/N=C/C5=CC=CC=C5O)=O)[C@]2(C)[C@H](C)CC3	6.08
O=C(CC1)[C@]2([H])[C@@]31[C@@H](C)[C@H](O)[C@](C)(C=C)C[C@@H](OC(CSC4=NN=C(C)N4/N=C/C5=CC=CC(O)=C5)=O)[C@]2(C)[C@H](C)CC3	6.08
O=C(CC1)[C@]2([H])[C@@]31[C@@H](C)[C@H](O)[C@](C)(C=C)C[C@@H](OC(CSC4=NN=C(C)N4/N=C/C5=CC=CC=C5[N+]([O-])=O)=O)[C@]2(C)[C@H](C)CC3	6.08
O=C(CC1)[C@]2([H])[C@@]31[C@@H](C)[C@H](O)[C@](C)(C=C)C[C@@H](OC(CSC4=NN(C(C5=CC=CC=C5C)=O)C(N)=N4)=O)[C@]2(C)[C@H](C)CC3	6.08
O=C(CC1)[C@]2([H])[C@@]31[C@@H](C)[C@H](O)[C@](C)(C=C)C[C@@H](OC(N4C(C(C=CC=C5NC(C(O)=O)C(C)C)=C5C4)=O)=O)[C@]2(C)[C@H](C)CC3	6.08
O=C(CC1)[C@]2([H])[C@@]31[C@@H](C)[C@H](O)[C@](C)(C=C)C[C@@H](OC(CSC4=NN=C(C)N4/N=C/C5=CC=C(C)C=C5)=O)[C@]2(C)[C@H](C)CC3	6.07
O=C(CC1)[C@]2([H])[C@@]31[C@@H](C)[C@H](O)[C@](C)(C=C)C[C@@H](OC(CSC4=CC(CO)=CC=N4)=O)[C@]2(C)[C@H](C)CC3	6.14
O=C1CC[C@]2(CC[C@H]3C)[C@]1([H])[C@]3(C)[C@H](OC(CN4CCN(C(CN(CCC)CCC)=O)CC4)=O)C[C@@](C)(C=C)[C@@H](O)[C@@H]2C	6.07
CC1C23C(C(CC3)=O)C(C(CC(C=C)(C)C1O)OC(CN4CCN(C(CN5CCN(C)CC5)=O)CC4)=O)(C(CC2)C)C	6.07
O=C1CC[C@]2(CC[C@H]3C)[C@]1([H])[C@]3(C)[C@H](OC(CN4CCN(C(NCC5=C([CF3])C=CC=C5)=O)CC4)=O)C[C@@](C)(C=C)[C@@H](O)[C@@H]2C	6.07
O=C(CC1)[C@]2([H])[C@@]31[C@@H](C)[C@H](O)[C@](C)(C=C)C[C@@H](OC(CN4CCN(C(NCC5=CC=CC=C5)=O)CC4)=O)[C@]2(C)[C@H](C)CC3	6.06
O=C1CC[C@]2(CC[C@H]3C)[C@]1([H])[C@]3(C)[C@H](OC(CN4CCN(C(CNC5=CC=CC=C5)=O)CC4)=O)C[C@@](C)(C=C)[C@@H](O)[C@@H]2C	6.06
O=C(CC1)[C@]2([H])[C@@]31[C@@H](C)[C@H](O)[C@](C)(C=C)C[C@@H](OC(CSC4=NN=C(C)N4/N=C/C5=CC=CC=C5)=O)[C@]2(C)[C@H](C)CC3	6.06
O=C1CC[C@]2(CC[C@H]3C)[C@]1([H])[C@]3(C)[C@H](OC(CN4CCN(C5=CC=C([N+]([O-])=O)C=C5)CC4)=O)C[C@@](C)(C=C)[C@@H](O)[C@@H]2C	6.06
O=C(CC1)[C@]2([H])[C@@]31[C@@H](C)[C@H](O)[C@](C)(C=C)C[C@@H](OC(N4C(C(C=C(NC(C)C(O)=O)C=C5)=C5C4)=O)=O)[C@]2(C)[C@H](C)CC3	6.05
O=C(CC1)[C@]2([H])[C@@]31[C@@H](C)[C@H](O)[C@](C)(C=C)C[C@@H](OC(N4C(C(C=CC(N5CCOCC5)=C6)=C6C4)=O)=O)[C@]2(C)[C@H](C)CC3	6.05
O=C1CC[C@]2(CC[C@H]3C)[C@]1([H])[C@]3(C)[C@H](OC(CN4CCN(C(CN(CC)CC)=O)CC4)=O)C[C@@](C)(C=C)[C@@H](O)[C@@H]2C	6.05
O=C(CC1)[C@]2([H])[C@@]31[C@@H](C)[C@H](O)[C@](C)(C=C)C[C@@H](OC(CS(SC4=NN=C(S)S4)=O)=O)[C@]2(C)[C@H](C)CC3	6.05
O=C1CC[C@]2(CC[C@H]3C)[C@]1([H])[C@]3(C)[C@H](OC(CN4CCN(C(CN5CCCC5)=O)CC4)=O)C[C@@](C)(C=C)[C@@H](O)[C@@H]2C	6.05
O=C(CC1)[C@]2([H])[C@@]31[C@@H](C)[C@H](O)[C@](C)(C=C)C[C@@H](OC(CSCC4=C(O)C(C=CO4)=O)=O)[C@]2(C)[C@H](C)CC3	6.14
O=C(CC1)[C@]2([H])[C@@]31[C@@H](C)[C@H](O)[C@](C)(C=C)C[C@@H](OC(CS(S(C4=NN=C(C)S4)=O)=O)=O)[C@]2(C)[C@H](C)CC3	6.05
O=C(CC1)[C@]2([H])[C@@]31[C@@H](C)[C@H](O)[C@](C)(C=C)C[C@@H](OC(CN4CCN(C5=CC=CC=C5OC)CC4)=O)[C@]2(C)[C@H](C)CC3	6.04
O=C(CC1)[C@]2([H])[C@@]31[C@@H](C)[C@H](O)[C@](C)(C=C)C[C@@H](OC(CSC4=NN=C(C5=CC=C(C)C=C5)O4)=O)[C@]2(C)[C@H](C)CC3	6.04
O=C(CC1)[C@]2([H])[C@@]31[C@@H](C)[C@H](O)[C@](C)(C=C)C[C@@H](OC(CSC4=CC(C=NN)=CN=C4)=O)[C@]2(C)[C@H](C)CC3	6.14
O=C1CC[C@]2(CC[C@H]3C)[C@]1([H])[C@]3(C)[C@H](OC(CSC4=NC(N5CCN(C)CC5)=CC(C)=N4)=O)C[C@@](C)(C=C)[C@@H](O)[C@@H]2C	6.13
O=C(CC1)[C@]2([H])[C@@]31[C@@H](C)[C@H](O)[C@](C)(C=C)C[C@@H](OC(CN4CCN(C5=CC=CC=C5O)CC4)=O)[C@]2(C)[C@H](C)CC3	6.03
O=C1CC[C@]2(CC[C@H]3C)[C@]1([H])[C@]3(C)[C@H](OC(CN4CCN(C5=CC=CC=C5O)CC4)=O)C[C@@](C)(C=C)[C@@H](O)[C@@H]2C	6.03
O=C(CC1)[C@]2([H])[C@@]31[C@@H](C)[C@H](O)[C@](C)(C=C)C[C@@H](OC(CS(S(C4=NC=CC=C4)=O)=O)=O)[C@]2(C)[C@H](C)CC3	6.03
O=C(CC1)[C@]2([H])[C@@]31[C@@H](C)[C@H](O)[C@](C)(C=C)C[C@@H](OC(CN4N=NC(C5=CC=CC(CO)=C5)=C4)=O)[C@]2(C)[C@H](C)CC3	6.03
O=C1CC[C@]2(CC[C@H]3C)[C@]1([H])[C@]3(C)[C@H](OC(CN4CCN(C(CN(C)C)=O)CC4)=O)C[C@@](C)(C=C)[C@@H](O)[C@@H]2C	6.03
O=C(CC1)[C@]2([H])[C@@]31[C@@H](C)[C@H](O)[C@](C)(C=C)C[C@@H](OC(CN4N=NC(C5=CC=CC=C5C#N)=C4)=O)[C@]2(C)[C@H](C)CC3	6.03
O=C(CC1)[C@]2([H])[C@@]31[C@@H](C)[C@H](O)[C@](C)(C=C)C[C@@H](OC(CS(SC4=NNC(N)=N4)=O)=O)[C@]2(C)[C@H](C)CC3	6.02
O=C(CC1)[C@]2([H])[C@@]31[C@@H](C)[C@H](O)[C@](C)(C=C)C[C@@H](OC(N4C(C(C=CC=C5[N+]([O-])=O)=C5C4)=O)=O)[C@]2(C)[C@H](C)CC3	6.02
O=C(CC1)[C@]2([H])[C@@]31[C@@H](C)[C@H](O)[C@](C)(C=C)C[C@@H](OC(N4C(C(C=C([N+]([O-])=O)C=C5)=C5C4)=O)=O)[C@]2(C)[C@H](C)CC3	6.02
O=C1CC[C@]2(CC[C@H]3C)[C@]1([H])[C@]3(C)[C@H](OC(CN4CCN(C5=CC=CC=C5)CC4)=O)C[C@@](C)(C=C)[C@@H](O)[C@@H]2C	6.02
[H][C@]12CC[C@](C[C@](C1)(SCC(O[C@@]3(C[C@@](C=C)([C@@](O)([C@](C)([C@]45CCC([C@]4([C@]3([C@](C)(CC5)[H])C)[H])=O)[H])[H])C)[H])=O)[H])(N2C)[H]	6.02
O=C1CC[C@]2(CC[C@H]3C)[C@]1([H])[C@]3(C)[C@H](OC(CSC4=CC=CC(C(O)=O)=C4)=O)C[C@@](C)(C=C)[C@@H](O)[C@@H]2C	6.01
O=C(CC1)[C@]2([H])[C@@]31[C@@H](C)[C@H](O)[C@](C)(C=C)C[C@@H](OC(CSC4=CC(C=NN)=CC=N4)=O)[C@]2(C)[C@H](C)CC3	6.01
O=C(CC1)[C@]2([H])[C@@]31[C@@H](C)[C@H](O)[C@](C)(C=C)C[C@@H](OC(CS(SC4=NNC=N4)=O)=O)[C@]2(C)[C@H](C)CC3	6.01
O=C(CC1)[C@]2([H])[C@@]31[C@@H](C)[C@H](O)[C@](C)(C=C)C[C@@H](OC(CS(SC4=NNC=N4)=O)=O)[C@]2(C)[C@H](C)CC3	6.01
O=C(CC1)[C@]2([H])[C@@]31[C@@H](C)[C@H](O)[C@](C)(C=C)C[C@@H](OC(CSCC4=CC(C(O)=C(CN5CCCC(O)C5)O4)=O)=O)[C@]2(C)[C@H](C)CC3	6.10
O=C1CC[C@]2(CC[C@H]3C)[C@]1([H])[C@]3(C)[C@H](OC(CSCCN(CC)CC)=O)C[C@@](C)(C=C)[C@@H](O)[C@@H]2C	5.99
O=C(CC1)[C@]2([H])[C@@]31[C@@H](C)[C@H](O)[C@](C)(C=C)C[C@@H](OC(CSC4=NN=C(N)S4)=O)[C@]2(C)[C@H](C)CC3	5.99
O=C(CC1)[C@]2([H])[C@@]31[C@@H](C)[C@H](O)[C@](C)(C=C)C[C@@H](OC(CSC4=CC(C#N)=CC=N4)=O)[C@]2(C)[C@H](C)CC3	6.12
O=C(CC1)[C@]2([H])[C@@]31[C@@H](C)[C@H](O)[C@](C)(C=C)C[C@@H](OC(CNCCC4=CC=CS4)=O)[C@]2(C)[C@H](C)CC3	5.99
O=C(CC1)[C@]2([H])[C@@]31[C@@H](C)[C@H](O)[C@](C)(C=C)C[C@@H](OC(CSC4=NC=CC(C)=N4)=O)[C@]2(C)[C@H](C)CC3	5.99
O=C(CC1)[C@]2([H])[C@@]31[C@@H](C)[C@H](O)[C@](C)(C=C)C[C@@H](OC(CSC4=NC=CC(C)=N4)=O)[C@]2(C)[C@H](C)CC3	5.99
O=C(CC1)[C@]2([H])[C@@]31[C@@H](C)[C@H](O)[C@](C)(C=C)C[C@@H](OC(CNCCS(=O)(C)=O)=O)[C@]2(C)[C@H](C)CC3	5.99

O=C(CC1)[C@]2([H])[C@@]31[C@@H](C)[C@H](O)[C@](C)(C=C)C[C@@H](OC(CNCCC4=CC=CC=C4)=O)[C@]2(C)[C@H](C)CC3	5.98
O=C(CC1)[C@]2([H])[C@@]31[C@@H](C)[C@H](O)[C@](C)(C=C)C[C@@H](OC(CSC4=NC=CS4)=O)[C@]2(C)[C@H](C)CC3	5.98
O=C(CC1)[C@]2([H])[C@@]31[C@@H](C)[C@H](O)[C@](C)(C=C)C[C@@H](OC(CSC4=CC=CS4)=O)[C@]2(C)[C@H](C)CC3	5.98
O=C(CC1)[C@]2([H])[C@@]31[C@@H](C)[C@H](O)[C@](C)(C=C)C[C@@H](OC(CSC4=NC=CN4C)=O)[C@]2(C)[C@H](C)CC3	5.98
O=C(CC1)[C@]2([H])[C@@]31[C@@H](C)[C@H](O)[C@](C)(C=C)C[C@@H](OC(CSCC4=CC=CO4)=O)[C@]2(C)[C@H](C)CC3	5.98
O=C(CC1)[C@]2([H])[C@@]31[C@@H](C)[C@H](O)[C@](C)(C=C)C[C@@H](OC(CSC4=NC=CN4C)=O)[C@]2(C)[C@H](C)CC3	5.98
O=C(CC1)[C@]2([H])[C@@]31[C@@H](C)[C@H](O)[C@](C)(C=C)C[C@@H](OC(CSCC4=CC=CO4)=O)[C@]2(C)[C@H](C)CC3	5.98
O=C(CC1)[C@]2([H])[C@@]31[C@@H](C)[C@H](O)[C@](C)(C=C)C[C@@H](OC(CNCCSC)=O)[C@]2(C)[C@H](C)CC3	5.96
O=C(CC1)[C@]2([H])[C@@]31[C@@H](C)[C@H](O)[C@](C)(C=C)C[C@@H](OC(CSC4=CC(F)=CN=C4)=O)[C@]2(C)[C@H](C)CC3	6.12
O=C(CC1)[C@]2([H])[C@@]31[C@@H](C)[C@H](O)[C@](C)(C=C)C[C@@H](OC(CN4CCOCC4)=O)[C@]2(C)[C@H](C)CC3	5.95
O=C(CC1)[C@]2([H])[C@@]31[C@@H](C)[C@H](O)[C@](C)(C=C)C[C@@H](OC(CSC4=CC=C(N)C=N4)=O)[C@]2(C)[C@H](C)CC3	6.11
O=C(CC1)[C@]2([H])[C@@]31[C@@H](C)[C@H](O)[C@](C)(C=C)C[C@@H](OC(CN4CCN(C(/C=C/C5=CC=CC=C5Cl)=O)CC4)=O)[C@]2(C)[C@H](C)CC3	5.91
O=C(CC1)[C@]2([H])[C@@]31[C@@H](C)[C@H](O)[C@](C)(C=C)C[C@@H](OC(CN4CCN(C(/C=C/C5=CC(Cl)=CC=C5)=O)CC4)=O)[C@]2(C)[C@H](C)CC3	5.91
O=C(CC1)[C@]2([H])[C@@]31[C@@H](C)[C@H](O)[C@](C)(C=C)C[C@@H](OC(CSC4=C(NC(CN5N=NC(CN6CCN(C7=CC=CC=C7Cl)CC6)=C5)=O)C=CC=C4)=O)[C@]2(C)[C@H](C)CC3	5.90
O=C(CC1)[C@]2([H])[C@@]31[C@@H](C)[C@H](O)[C@](C)(C=C)C[C@@H](OC(CSC4=C(NC(CN5N=NC(CN6CCN(C7=CC=C(OC)C=C7)CC6)=C5)=O)C=CC=C4)=O)[C@]2(C)[C@H](C)CC3	5.90
O=C(CC1)[C@]2([H])[C@@]31[C@@H](C)[C@H](O)[C@](C)(C=C)C[C@@H](OC(CN4CCN(C(/C=C/C5=CC(F)=CC=C5)=O)CC4)=O)[C@]2(C)[C@H](C)CC3	5.90
O=C(CC1)[C@]2([H])[C@@]31[C@@H](C)[C@H](O)[C@](C)(C=C)C[C@@H](OC(CN4CCN(C(/C=C/C5=CC(C)=CC=C5)=O)CC4)=O)[C@]2(C)[C@H](C)CC3	5.90
O=C(CC1)[C@]2([H])[C@@]31[C@@H](C)[C@H](O)[C@](C)(C=C)C[C@@H](OC(CSC4=C(NC(CN5N=NC(CN6CCN(C7=CC=CC=C7F)CC6)=C5)=O)C=CC=C4)=O)[C@]2(C)[C@H](C)CC3	5.90
O=C(CC1)[C@]2([H])[C@@]31[C@@H](C)[C@H](O)[C@](C)(C=C)C[C@@H](OC(CSC4=C(NC(CN5N=NC(CN6CCN(C7=CC=CC(F)=C7)CC6)=C5)=O)C=CC=C4)=O)[C@]2(C)[C@H](C)CC3	5.90
O=C(CC1)[C@]2([H])[C@@]31[C@@H](C)[C@H](O)[C@](C)(C=C)C[C@@H](OC(CSC4=C(NC(CN5N=NC(CN6CCN(C7=CC=C(F)C=C7)CC6)=C5)=O)C=CC=C4)=O)[C@]2(C)[C@H](C)CC3	5.90
O=C(CC1)[C@]2([H])[C@@]31[C@@H](C)[C@H](O)[C@](C)(C=C)C[C@@H](OC(CSC4=C(NC(CN5N=NC(CN6CCN(C7=CC=CC=C7C)CC6)=C5)=O)C=CC=C4)=O)[C@]2(C)[C@H](C)CC3	5.89
O=C(CC1)[C@]2([H])[C@@]31[C@@H](C)[C@H](O)[C@](C)(C=C)C[C@@H](OC(CSC4=C(NC(CN5N=NC(CN6CCN(C7=CC=CC(C)=C7)CC6)=C5)=O)C=CC=C4)=O)[C@]2(C)[C@H](C)CC3	5.89
O=C(CC1)[C@]2([H])[C@@]31[C@@H](C)[C@H](O)[C@](C)(C=C)C[C@@H](OC(CSC4=C(NC(CN5N=NC(CN6CCN(C7=CC=C(C)C=C7)CC6)=C5)=O)C=CC=C4)=O)[C@]2(C)[C@H](C)CC3	5.89
O=C(CC1)[C@]2([H])[C@@]31[C@@H](C)[C@H](O)[C@](C)(C=C)C[C@@H](OC(CS[C@H]4CC[C@H](C(OCC)=O)C[C@H]4NS(=O)(C5=CC=C(Br)C=C5)=O)=O)[C@]2(C)[C@H](C)CC3	5.89
O=C(CC1)[C@]2([H])[C@@]31[C@@H](C)[C@H](O)[C@](C)(C=C)C[C@@H](OC(CSC4=C(N)C=CC=N4)=O)[C@]2(C)[C@H](C)CC3	5.89
O=C(CC1)[C@]2([H])[C@@]31[C@@H](C)[C@H](O)[C@](C)(C=C)C[C@@H](OC(CN4CCN(C(CN5N=NC(CN6CCN(C7=CC=C([N+]([O-])=O)C=C7)CC6)=C5)=O)CC4)=O)[C@]2(C)[C@H](C)CC3	5.89
O=C(CC1)[C@]2([H])[C@@]31[C@@H](C)[C@H](O)[C@](C)(C=C)C[C@@H](OC(CSC4CCN(C(CCN5C=NC6=C5N=C(N)N=C6N7CCC(N)CC7)=O)CC4)=O)[C@]2(C)[C@H](C)CC3	5.88
O=C(CC1)[C@]2([H])[C@@]31[C@@H](C)[C@H](O)[C@](C)(C=C)C[C@@H](OC(CSC4CCN(C(CCN5C=NC6=C5N=C(N)N=C6N7CC(N)CCC7)=O)CC4)=O)[C@]2(C)[C@H](C)CC3	5.88
O=C(CC1)[C@]2([H])[C@@]31[C@@H](C)[C@H](O)[C@](C)(C=C)C[C@@H](OC(CSC4CCN(C(CCN5C=NC6=C5N=C(N)N=C6N7CC(NC)CC7)=O)CC4)=O)[C@]2(C)[C@H](C)CC3	5.88
O=C(CC1)[C@]2([H])[C@@]31[C@@H](C)[C@H](O)[C@](C)(C=C)C[C@@H](OC(CN4CCN(C(CN5N=NC(CN6CCN(C7=CC=C(Cl)C=C7)CC6)=C5)=O)CC4)=O)[C@]2(C)[C@H](C)CC3	5.88
O=C(CC1)[C@]2([H])[C@@]31[C@@H](C)[C@H](O)[C@](C)(C=C)C[C@@H](OC(CSC4CCCN(C(CCN5C=NC6=C5N=C(N)N=C6N7CCNCC7)=O)C4)=O)[C@]2(C)[C@H](C)CC3	5.88
O=C(CC1)[C@]2([H])[C@@]31[C@@H](C)[C@H](O)[C@](C)(C=C)C[C@@H](OC(CSC4CCN(C(CCN5C=NC6=C5N=C(N)N=C6N7CC(N)CC7)=O)CC4)=O)[C@]2(C)[C@H](C)CC3	5.88
O=C(CC1)[C@]2([H])[C@@]31[C@@H](C)[C@H](O)[C@](C)(C=C)C[C@@H](OC(CSC4CCN(C(CCN5C=NC6=C5N=C(N)N=C6N7CC(NC)C7)=O)CC4)=O)[C@]2(C)[C@H](C)CC3	5.88
O=C(CC1)[C@]2([H])[C@@]31[C@@H](C)[C@H](O)[C@](C)(C=C)C[C@@H](OC(CNCCCC4=CC=CC=C4)=O)[C@]2(C)[C@H](C)CC3	5.88
O=C(CC1)[C@]2([H])[C@@]31[C@@H](C)[C@H](O)[C@](C)(C=C)C[C@@H](OC(CS[C@H]4CC[C@H](C(OCC)=O)C[C@H]4NS(=O)(C5=CC=C([N+]([O-])=O)C=C5)=O)=O)[C@]2(C)[C@H](C)CC3	5.87
O=C(CC1)[C@]2([H])[C@@]31[C@@H](C)[C@H](O)[C@](C)(C=C)C[C@@H](OC(CN4CCN(C5=CC=C(C6NC(N)=NC(C7=CC=CC=C7[N+]([O-])=O)=C6)C=C5)CC4)=O)[C@]2(C)[C@H](C)CC3	5.87
O=C(CC1)[C@]2([H])[C@@]31[C@@H](C)[C@H](O)[C@](C)(C=C)C[C@@H](OC(CSC(C4)CCN4C(CCN5C=NC6=C5N=C(N)N=C6N7CCNCC7)=O)=O)[C@]2(C)[C@H](C)CC3	5.87
O=C(CC1)[C@]2([H])[C@@]31[C@@H](C)[C@H](O)[C@](C)(C=C)C[C@@H](OC(CS[C@H](C4)CCN4C(CCN5C=NC6=C5N=C(N)N=C6N7CCNCC7)=O)=O)[C@]2(C)[C@H](C)CC3	5.87
O=C(CC1)[C@]2([H])[C@@]31[C@@H](C)[C@H](O)[C@](C)(C=C)C[C@@H](OC(CSC4CCN(C(CCN5C=NC6=C5N=C(N)N=C6N7CC(N)C7)=O)CC4)=O)[C@]2(C)[C@H](C)CC3	5.87
O=C(CC1)[C@]2([H])[C@@]31[C@@H](C)[C@H](O)[C@](C)(C=C)C[C@@H](OC(CS[C@H]4CC[C@H](C(OCC)=O)C[C@H]4NS(=O)(C5=CC=C(C#N)C=C5)=O)=O)[C@]2(C)[C@H](C)CC3	5.86
O=C1CC[C@]2(CC[C@H]3C)[C@]1([H])[C@]3(C)[C@H](OC(CSC4=NC(NC=C5)=C5C(NC(CN6CCN(C7=CC=CC=C7)CC6)=O)=N4)=O)C[C@@](C)(C=C)[C@@H](O)[C@@H]2C	5.86
O=C(CC1)[C@]2([H])[C@@]31[C@@H](C)[C@H](O)[C@](C)(C=C)C[C@@H](OC(CSC4=C(NC(CN5CCN(C6=CC=CC(Cl)=C6)CC5)=O)C=CC=C4)=O)[C@]2(C)[C@H](C)CC3	5.86
O=C(CC1)[C@]2([H])[C@@]31[C@@H](C)[C@H](O)[C@](C)(C=C)C[C@@H](OC(CS[C@H]4CC[C@H](C(OCC)=O)C[C@H]4NS(=O)(C5=CC=C(F)C=C5)=O)=O)[C@]2(C)[C@H](C)CC3	5.86
O=C(CC1)[C@]2([H])[C@@]31[C@@H](C)[C@H](O)[C@](C)(C=C)C[C@@H](OC(CN4CCN(C(NCC5=CC=C(NC(CN6CCOCC6)=O)C=C5)=O)CC4)=O)[C@]2(C)[C@H](C)CC3	5.86

O=C(CC1)[C@]2([H])[C@@]31[C@@H](C)[C@H](O)[C@](C)(C=C)C[C@@H](OC(CS[C@H]4CC[C@H](C(OCC)=O)C[C@H]4NS(=O)(C5=CC=C(C)C=C5)=O)=O)[C@]2(C)[C@H](C)CC3	5.86
O=C(CC1)[C@]2([H])[C@@]31[C@@H](C)[C@H](O)[C@](C)(C=C)C[C@@H](OC(CN4N=NC(C5=CC=CC=C5CN6C7=NC=NC(N(CC)CC)=C7N=C6)=C4)=O)[C@]2(C)[C@H](C)CC3	5.85
O=C(CC1)[C@]2([H])[C@@]31[C@@H](C)[C@H](O)[C@](C)(C=C)C[C@@H](OC(CN4N=NC(C5=CC=CC(CN6C7=NC=NC(N(CC)CC)=C7N=C6)=C5)=C4)=O)[C@]2(C)[C@H](C)CC3	5.85
O=C(CC1)[C@]2([H])[C@@]31[C@@H](C)[C@H](O)[C@](C)(C=C)C[C@@H](OC(CSC4=C(NC(CN5N=NC(CN6CCC(O)CC6)=C5)=O)C=CC=C4)=O)[C@]2(C)[C@H](C)CC3	5.85
O=C(CC1)[C@]2([H])[C@@]31[C@@H](C)[C@H](O)[C@](C)(C=C)C[C@@H](OC(CN4CCN(CC4)C(CCN5C=NC6=C5N=CN=C6N7CCC(C7)N)=O)=O)[C@]2(C)[C@H](C)CC3	5.85
O=C(CC1)[C@]2([H])[C@@]31[C@@H](C)[C@H](O)[C@](C)(C=C)C[C@@H](OC(CSC4=C(NC(CN5CCN(C6=C(O)C=CC=C6)CC5)=O)C=CC=C4)=O)[C@]2(C)[C@H](C)CC3	5.85
O=C(CC1)[C@]2([H])[C@@]31[C@@H](C)[C@H](O)[C@](C)(C=C)C[C@@H](OC(CSC4=C(NC(CN5CCN(C6=CC=C(O)C=C6)CC5)=O)C=CC=C4)=O)[C@]2(C)[C@H](C)CC3	5.85
O=C(CC1)[C@]2([H])[C@@]31[C@@H](C)[C@H](O)[C@](C)(C=C)C[C@@H](OC(CN4CCN(C5=CC=C(C(/C=C/C6=CC=CC([N+]([O-])=O)=C6)=O)C=C5)CC4)=O)[C@]2(C)[C@H](C)CC3	5.84
O=C(CC1)[C@]2([H])[C@@]31[C@@H](C)[C@H](O)[C@](C)(C=C)C[C@@H](OC(CN4CCN(C5=CC=C(/C=C/C(C6=CC=CC=C6[N+]([O-])=O)=O)C=C5)CC4)=O)[C@]2(C)[C@H](C)CC3	5.84
O=C(CC1)[C@]2([H])[C@@]31[C@@H](C)[C@H](O)[C@](C)(C=C)C[C@@H](OC(CN4CCN(C5=CC=C(C(/C=C/C6=CC=C(C=C6)N(C)C)=O)C=C5)CC4)=O)[C@]2(C)[C@H](C)CC3	5.84
O=C1CC[C@]2(CC[C@H]3C)[C@]1([H])[C@]3(C)[C@H](OC(CSC4=NC(NC=C5)=C5C(NC(CN6CCC(N(C)C)CC6)=O)=N4)=O)C[C@@](C)(C=C)[C@@H](O)[C@@H]2C	5.84
O=C(CC1)[C@]2([H])[C@@]31[C@@H](C)[C@H](O)[C@](C)(C=C)C[C@@H](OC(N4CC5=C(C=CC=C5OCCCCN6C=NC(C7=CC=CN=C7)=C6)C4=O)=O)[C@]2(C)[C@H](C)CC3	5.84
O=C(CC1)[C@]2([H])[C@@]31[C@@H](C)[C@H](O)[C@](C)(C=C)C[C@@H](OC(CN4CCN(C(NC5=CC=CC(NC(OC(C)(C)C)=O)=C5)=O)CC4)=O)[C@]2(C)[C@H](C)CC3	5.83
O=C(CC1)[C@]2([H])[C@@]31[C@@H](C)[C@H](O)[C@](C)(C=C)C[C@@H](OC(CN4CCN(C(CN5CCN(C6=CC=CC=C6OC)CC5)=O)CC4)=O)[C@]2(C)[C@H](C)CC3	5.83
O=C1CC[C@]2(CC[C@H]3C)[C@]1([H])[C@]3(C)[C@H](OC(CSC4=NC(N)=CC(NC(CN5CCCC(CCO)C5)=O)=N4)=O)C[C@@](C)(C=C)[C@@H](O)[C@@H]2C	5.83
O=C(CC1)[C@]2([H])[C@@]31[C@@H](C)[C@H](O)[C@](C)(C=C)C[C@@H](OC(CSC4=NC(NC(CN5CCC(CCO)CC5)=O)=CC(N)=N4)=O)[C@]2(C)[C@H](C)CC3	5.83
O=C(CC1)[C@]2([H])[C@@]31[C@@H](C)[C@H](O)[C@](C)(C=C)C[C@@H](OC(CSC4=NN(C(C5=C(F)C(F)=C(F)C(F)=C5F)=O)C(N)=N4)=O)[C@]2(C)[C@H](C)CC3	5.83
O=C1CC[C@]2(CC[C@H]3C)[C@]1([H])[C@]3(C)[C@H](OC(CSC4=NC(NC=C5)=C5C(NC(CN6CCN(C)CC6)=O)=N4)=O)C[C@@](C)(C=C)[C@@H](O)[C@@H]2C	5.82
O=C1CC[C@]2(CC[C@H]3C)[C@]1([H])[C@]3(C)[C@H](OC(CSC4=NC(NC=C5)=C5C(NC(CNC6CCCCC6)=O)=N4)=O)C[C@@](C)(C=C)[C@@H](O)[C@@H]2C	5.82
CC1C23C(C(CC3)=O)C(C(CC(C=C)(C)C1O)OC(CN4CCN(C(CN5CCN(C6=C(O)C=CC=C6)CC5)=O)CC4)=O)(C(CC2)C)C	5.82
O=C(CC1)[C@]2([H])[C@@]31[C@@H](C)[C@H](O)[C@](C)(C=C)C[C@@H](OC(CSC4=NN(C(C5=CC=C(OC(F)(F)F)C=C5)=O)C(N)=N4)=O)[C@]2(C)[C@H](C)CC3	5.82
O=C(CC1)[C@]2([H])[C@@]31[C@@H](C)[C@H](O)[C@](C)(C=C)C[C@@H](OC(CN4CCN(C(/C=C/C5=CC(Br)=CC=C5)=O)CC4)=O)[C@]2(C)[C@H](C)CC3	5.82
O=C(CC1)[C@]2([H])[C@@]31[C@@H](C)[C@H](O)[C@](C)(C=C)C[C@@H](OC(CSCC4=CSC(NC(CN(CCO)CCO)=O)=N4)=O)[C@]2(C)[C@H](C)CC3	5.81
O=C(CC1)[C@]2([H])[C@@]31[C@@H](C)[C@H](O)[C@](C)(C=C)C[C@@H](OC(CSC4=NN(C(C5=CC(Cl)=CC(Cl)=C5)=O)C(N)=N4)=O)[C@]2(C)[C@H](C)CC3	5.81
O=C(CC1)[C@]2([H])[C@@]31[C@@H](C)[C@H](O)[C@](C)(C=C)C[C@@H](OC(CN4N=NC(CN5CCN(C6=CC=CC=C6[N+]([O-])=O)CC5)=C4)=O)[C@]2(C)[C@H](C)CC3	5.81
O=C(CC1)[C@]2([H])[C@@]31[C@@H](C)[C@H](O)[C@](C)(C=C)C[C@@H](OC(CSC4=NN(C(C5=CC=C(C(F)(F)F)C=C5)=O)C(N)=N4)=O)[C@]2(C)[C@H](C)CC3	5.81
O=C(CC1)[C@]2([H])[C@@]31[C@@H](C)[C@H](O)[C@](C)(C=C)C[C@@H](OC(CSC4=NN=C(C)N4/N=C/C5=CC=C(N6CCCC6)C=C5)=O)[C@]2(C)[C@H](C)CC3	5.81
O=C(CC1)[C@]2([H])[C@@]31[C@@H](C)[C@H](O)[C@](C)(C=C)C[C@@H](OC(CN4CCN(C(NCC5=C(C(F)(F)F)C=CC=C5)=O)CC4)=O)[C@]2(C)[C@H](C)CC3	5.81
O=C(CC1)[C@]2([H])[C@@]31[C@@H](C)[C@H](O)[C@](C)(C=C)C[C@@H](OC(CN4CCN(C(NC5=CC=C(Br)C=N5)=O)CC4)=O)[C@]2(C)[C@H](C)CC3	5.81
O=C(CC1)[C@]2([H])[C@@]31[C@@H](C)[C@H](O)[C@](C)(C=C)C[C@@H](OC(CN4N=NC(C5=CC=CC=C5CN(C(N6)=O)C=C(C)C6=O)=C4)=O)[C@]2(C)[C@H](C)CC3	5.81
O=C(CC1)[C@]2([H])[C@@]31[C@@H](C)[C@H](O)[C@](C)(C=C)C[C@@H](OC(CN4N=NC(C5=CC=CC(CN(C(N6)=O)C=C(C)C6=O)=C5)=C4)=O)[C@]2(C)[C@H](C)CC3	5.81
O=C(CC1)[C@]2([H])[C@@]31[C@@H](C)[C@H](O)[C@](C)(C=C)C[C@@H](OC(CSCC4=CSC(NC(CNCC(OC)=O)=O)=N4)=O)[C@]2(C)[C@H](C)CC3	5.80
O=C(CC1)[C@]2([H])[C@@]31[C@@H](C)[C@H](O)[C@](C)(C=C)C[C@@H](OC(CN4CCN(C(NC5=CC=CC(C(F)(F)F)=C5)=O)CC4)=O)[C@]2(C)[C@H](C)CC3	5.80
O=C(CC1)[C@]2([H])[C@@]31[C@@H](C)[C@H](O)[C@](C)(C=C)C[C@@H](OC(CSC4=NN(C(C5=CC=C(F)C=C5Cl)=O)C(N)=N4)=O)[C@]2(C)[C@H](C)CC3	5.80
O=C(CC1)[C@]2([H])[C@@]31[C@@H](C)[C@H](O)[C@](C)(C=C)C[C@@H](OC(CSC4=NN(C(C5=C(F)C=CC=C5Cl)=O)C(N)=N4)=O)[C@]2(C)[C@H](C)CC3	5.80
O=C(CC1)[C@]2([H])[C@@]31[C@@H](C)[C@H](O)[C@](C)(C=C)C[C@@H](OC(CSC4=NN=C(NC(C5=C(OC)C=CC=C5)=O)S4)=O)[C@]2(C)[C@H](C)CC3	5.80
O=C(CC1)[C@]2([H])[C@@]31[C@@H](C)[C@H](O)[C@](C)(C=C)C[C@@H](OC(CSC4=CC(NC(CN5CCC[C@H]5CN)=O)=CN=C4)=O)[C@]2(C)[C@H](C)CC3	5.80
O=C(CC1)[C@]2([H])[C@@]31[C@@H](C)[C@H](O)[C@](C)(C=C)C[C@@H](OC(CS(S(C4=NC5=C(C=CC(Cl)=C5)S4)=O)=O)=O)[C@]2(C)[C@H](C)CC3	5.80
O=C(CC1)[C@]2([H])[C@@]31[C@@H](C)[C@H](O)[C@](C)(C=C)C[C@@H](OC(CN4N=NC(C5=CC=CC(CC6=CC=CC(CO)=C6)=C5)=C4)=O)[C@]2(C)[C@H](C)CC3	5.80
O=C(CC1)[C@]2([H])[C@@]31[C@@H](C)[C@H](O)[C@](C)(C=C)C[C@@H](OC(CSC4=NN=C(C)N4/N=C/C5=CC=CC([N+]([O-])=O)=C5)=O)[C@]2(C)[C@H](C)CC3	5.80
O=C(CC1)[C@]2([H])[C@@]31[C@@H](C)[C@H](O)[C@](C)(C=C)C[C@@H](OC(CSC4=NN=C(C)N4/N=C/C5=CC=C([N+]([O-])=O)C=C5)=O)[C@]2(C)[C@H](C)CC3	5.80
O=C(CC1)[C@]2([H])[C@@]31[C@@H](C)[C@H](O)[C@](C)(C=C)C[C@@H](OC(CSCC4=CSC(NC(CNCCCO)=O)=N4)=O)[C@]2(C)[C@H](C)CC3	5.79
O=C(CC1)[C@]2([H])[C@@]31[C@@H](C)[C@H](O)[C@](C)(C=C)C[C@@H](OC(CSC4=C(NC(C5=C(OC)C=CC=C5)=O)C=CC=C4)=O)[C@]2(C)[C@H](C)CC3	5.79
O=C(CC1)[C@]2([H])[C@@]31[C@@H](C)[C@H](O)[C@](C)(C=C)C[C@@H](OC(CSC4=C(NC(C5=CC=CC(OC)=C5)=O)C=CC=C4)=O)[C@]2(C)[C@H](C)CC3	5.79
O=C(CC1)[C@]2([H])[C@@]31[C@@H](C)[C@H](O)[C@](C)(C=C)C[C@@H](OC(CSC4=C(NC(C5=CC=C(OC)C=C5)=O)C=CC=C4)=O)[C@]2(C)[C@H](C)CC3	5.79
O=C(CC1)[C@]2([H])[C@@]31[C@@H](C)[C@H](O)[C@](C)(C=C)C[C@@H](OC(CN4N=NC(CN5CCN(C6=CC=CC=C6C)CC5)=C4)=O)[C@]2(C)[C@H](C)CC3	5.79
O=C(CC1)[C@]2([H])[C@@]31[C@@H](C)[C@H](O)[C@](C)(C=C)C[C@@H](OC(CN4N=NC(CN5CCN(C6=CC=CC(C)=C6)CC5)=C4)=O)[C@]2(C)[C@H](C)CC3	5.79
O=C1CC[C@]2(CC[C@H]3C)[C@]1([H])[C@]3(C)[C@H](OC(CN4CCN(C([C@H](C)NC(OC(C)(C)C)=O)=O)CC4)=O)C[C@@](C)(C=C)[C@@H](O)[C@@H]2C	5.79
O=C(CC1)[C@]2([H])[C@@]31[C@@H](C)[C@H](O)[C@](C)(C=C)C[C@@H](OC(CN4CCN(C(NC5=CC=CC6=C5C=CC=N6)=O)CC4)=O)[C@]2(C)[C@H](C)CC3	5.79
O=C(CC1)[C@]2([H])[C@@]31[C@@H](C)[C@H](O)[C@](C)(C=C)C[C@@H](OC(CSC4=NN=C(C)N4/N=C/C5=CC=CC=C5Cl)=O)[C@]2(C)[C@H](C)CC3	5.79

O=C(CC1)[C@]2([H])[C@@]31[C@@H](C)[C@H](O)[C@](C)(C=C)C[C@@H](OC(CSC4=NN=C(C)N4/N=C/C5=CC=CC(Cl)=C5)=O)[C@]2(C)[C@H](C)CC3	5.79
O=C(CC1)[C@]2([H])[C@@]31[C@@H](C)[C@H](O)[C@](C)(C=C)C[C@@H](OC(CSC4=NN(C(C5=CC=CC=C5OC)=O)C(N)=N4)=O)[C@]2(C)[C@H](C)CC3	5.79
N[C@@H](CC1=CC=C(O)C=C1)C(N(CC2)CCN2CC(O[C@@H]([C@]3(C)[C@H](C)CC4)C[C@@](C)(C=C)[C@@H](O)[C@H](C)[C@@]54[C@@]3([H])C(CC5)=O)=O)=O	5.79
O=C(CC1)[C@]2([H])[C@@]31[C@@H](C)[C@H](O)[C@](C)(C=C)C[C@@H](OC(CSCC4=CSC(NC(CNCCO)=O)=N4)=O)[C@]2(C)[C@H](C)CC3	5.78
O=C(CC1)[C@]2([H])[C@@]31[C@@H](C)[C@H](O)[C@](C)(C=C)C[C@@H](OC(CSC4=C(NC(C5=CC=C(F)C=C5)=O)C=CC=C4)=O)[C@]2(C)[C@H](C)CC3	5.78
O=C(CC1)[C@]2([H])[C@@]31[C@@H](C)[C@H](O)[C@](C)(C=C)C[C@@H](OC(CSC4=NN=C(NC(N5CCOCC5)=O)S4)=O)[C@]2(C)[C@H](C)CC3	5.78
O=C(CC1)[C@]2([H])[C@@]31[C@@H](C)[C@H](O)[C@](C)(C=C)C[C@@H](OC(CSCC4=CSC(NC([C@@]([H])(N)C(C)C)=O)=N4)=O)[C@]2(C)[C@H](C)CC3	5.78
O=C(CC1)[C@]2([H])[C@@]31[C@@H](C)[C@H](O)[C@](C)(C=C)C[C@@H](OC(CSC4=NN=C(C5=CC=CC(C(F)(F)F)=C5)N4)=O)[C@]2(C)[C@H](C)CC3	5.78
O=C(CC1)[C@]2([H])[C@@]31[C@@H](C)[C@H](O)[C@](C)(C=C)C[C@@H](OC(CSC(C)(C)CNC(C4=CC=C(Cl)C=C4)=O)=O)[C@]2(C)[C@H](C)CC3	5.78
O=C(CC1)[C@]2([H])[C@@]31[C@@H](C)[C@H](O)[C@](C)(C=C)C[C@@H](OC(CSC4=C(NC(C5=CC=CC(C)=C5)=O)C=CC=C4)=O)[C@]2(C)[C@H](C)CC3	5.78
O=C(CC1)[C@]2([H])[C@@]31[C@@H](C)[C@H](O)[C@](C)(C=C)C[C@@H](OC(CSC4=NN=C(C)N4/N=C/C5=CC=CC([N+]#[C-])=C5)=O)[C@]2(C)[C@H](C)CC3	5.78
O=C(CC1)[C@]2([H])[C@@]31[C@@H](C)[C@H](O)[C@](C)(C=C)C[C@@H](OC(CSC4=NN=C(C)N4/N=C/C5=CC=C([N+]#[C-])C=C5)=O)[C@]2(C)[C@H](C)CC3	5.78
O=C(CC1)[C@]2([H])[C@@]31[C@@H](C)[C@H](O)[C@](C)(C=C)C[C@@H](OC(CN4N=NC(/C=C/CN5C(N=CN=C6N)=C6N=C5)=C4)=O)[C@]2(C)[C@H](C)CC3	5.78
O=C(CC1)[C@]2([H])[C@@]31[C@@H](C)[C@H](O)[C@](C)(C=C)C[C@@H](OC(CSC4=NC(N)=CC(NC([C@H](N)C(C)C)=O)=N4)=O)[C@]2(C)[C@H](C)CC3	5.78
O=C1CC[C@]2(CC[C@H]3C)[C@]1([H])[C@]3(C)[C@H](OC(CSC4=NC(O)=CC(NC([C@@H](C)C(C)C)=O)=N4)=O)C[C@@](C)(C=C)[C@@H](O)[C@@H]2C	5.78
O=C(CC1)[C@]2([H])[C@@]31[C@@H](C)[C@H](O)[C@](C)(C=C)C[C@@H](OC(CSC4=NN(C(C5=CC=CC=C5F)=O)C(N)=N4)=O)[C@]2(C)[C@H](C)CC3	5.78
O=C(CC1)[C@]2([H])[C@@]31[C@@H](C)[C@H](O)[C@](C)(C=C)C[C@@H](OC(CSC4=NN=C(C)N4/N=C/C5=CC=C(F)C=C5)=O)[C@]2(C)[C@H](C)CC3	5.78
O=C(CC1)[C@]2([H])[C@@]31[C@@H](C)[C@H](O)[C@](C)(C=C)C[C@@H](OC(CS(S(C4=NC(OC)=CC(OC)=N4)=O)=O)=O)[C@]2(C)[C@H](C)CC3	5.78
O=C(CC1)[C@]2([H])[C@@]31[C@@H](C)[C@H](O)[C@](C)(C=C)C[C@@H](OC(CSC4=NC(C=C([Na])C=C5)=C5N4)=O)[C@]2(C)[C@H](C)CC3.O=S=O	5.78
O=C(CC1)[C@]2([H])[C@@]31[C@@H](C)[C@H](O)[C@](C)(C=C)C[C@@H](OC(CSC4=NC(C=C([Na])C=C5)=C5N4)=O)[C@]2(C)[C@H](C)CC3.O=S=O	5.78
O=C(CC1)[C@]2([H])[C@@]31[C@@H](C)[C@H](O)[C@](C)(C=C)C[C@@H](OC(CN4CCN(C(NC5=CC=C(OC)C=C5)=O)CC4)=O)[C@]2(C)[C@H](C)CC3	5.78
O=C(CC1)[C@]2([H])[C@@]31[C@@H](C)[C@H](O)[C@](C)(C=C)C[C@@H](OC(CN4CCN(C(NCC5=CC=C(N)C=C5)=O)CC4)=O)[C@]2(C)[C@H](C)CC3	5.77
O=C(CC1)[C@]2([H])[C@@]31[C@@H](C)[C@H](O)[C@](C)(C=C)C[C@@H](OC(N4C(C(C=C(NC(C(O)=O)C(C)C)C=C5)=C5C4)=O)=O)[C@]2(C)[C@H](C)CC3	5.77
O=C(CC1)[C@]2([H])[C@@]31[C@@H](C)[C@H](O)[C@](C)(C=C)C[C@@H](OC(CN4CCN(C(NCCC5=CC=CC=C5)=O)CC4)=O)[C@]2(C)[C@H](C)CC3	5.77
O=C1CC[C@]2(CC[C@H]3C)[C@]1([H])[C@]3(C)[C@H](OC(CSC4=NC(OCCN)=CC(C)=N4)=O)C[C@@](C)(C=C)[C@@H](O)[C@@H]2C	5.77
O=C(CC1)[C@]2([H])[C@@]31[C@@H](C)[C@H](O)[C@](C)(C=C)C[C@@H](OC(CSC4=NN=C(C)N4/N=C/C5=CC=CC=C5C)=O)[C@]2(C)[C@H](C)CC3	5.77
O=C(CC1)[C@]2([H])[C@@]31[C@@H](C)[C@H](O)[C@](C)(C=C)C[C@@H](OC(CSC4=NN=C(C)N4/N=C/C5=CC=CC(C)=C5)=O)[C@]2(C)[C@H](C)CC3	5.77
O=C(CC1)[C@]2([H])[C@@]31[C@@H](C)[C@H](O)[C@](C)(C=C)C[C@@H](OC(CN4CCN(C(/C=C/C5=CC=C(C)C=C5)=O)CC4)=O)[C@]2(C)[C@H](C)CC3	5.77
O=C(CC1)[C@]2([H])[C@@]31[C@@H](C)[C@H](O)[C@](C)(C=C)C[C@@H](OC(CN4CCN(C(/C(C)=C/C5=CC=CC=C5)=O)CC4)=O)[C@]2(C)[C@H](C)CC3	5.77
O=C1CC[C@]2(CC[C@H]3C)[C@]1([H])[C@]3(C)[C@H](OC(CSC4=NC(N)=CC(NC(CN(C)C)=O)=N4)=O)C[C@@](C)(C=C)[C@@H](O)[C@@H]2C	5.77
O=C(CC1)[C@]2([H])[C@@]31[C@@H](C)[C@H](O)[C@](C)(C=C)C[C@@H](OC(CSC4=NC(NC(CN(C)C)=O)=CC(N)=N4)=O)[C@]2(C)[C@H](C)CC3	5.77
O=C1CC[C@]2(CC[C@H]3C)[C@]1([H])[C@]3(C)[C@H](OC(CN4CCN(C(CN5CCN(C)CC5)=O)CC4)=O)C[C@@](C)(C=C)[C@@H](O)[C@@H]2C	5.77
O=C(CC1)[C@]2([H])[C@@]31[C@@H](C)[C@H](O)[C@](C)(C=C)C[C@@H](OC(CSC(C)(C)CNC(C4=C(N)C=CC=C4)=O)=O)[C@]2(C)[C@H](C)CC3	5.77
O=C(CC1)[C@]2([H])[C@@]31[C@@H](C)[C@H](O)[C@](C)(C=C)C[C@@H](OC(CSC(C)(C)CNC(C4=CC(C)=CC=C4)=O)=O)[C@]2(C)[C@H](C)CC3	5.77
O=C(CC1)[C@]2([H])[C@@]31[C@@H](C)[C@H](O)[C@](C)(C=C)C[C@@H](OC(CN4CCN(C(NC5=CC=CC(F)=C5)=O)CC4)=O)[C@]2(C)[C@H](C)CC3	5.77
O=C1CC[C@]2(CC[C@H]3C)[C@]1([H])[C@]3(C)[C@H](OC(CN4CCN(C(NC5=CC=CC(F)=C5)=O)CC4)=O)C[C@@](C)(C=C)[C@@H](O)[C@@H]2C	5.77
O=C(CC1)[C@]2([H])[C@@]31[C@@H](C)[C@H](O)[C@](C)(C=C)C[C@@H](OC(CN4CCN(C(NC5=CC=CC(N)=N5)=O)CC4)=O)[C@]2(C)[C@H](C)CC3	5.76
O=C(CC1)[C@]2([H])[C@@]31[C@@H](C)[C@H](O)[C@](C)(C=C)C[C@@H](OC(CN4CCN(C(NC5=CC=CC(N)=C5)=O)CC4)=O)[C@]2(C)[C@H](C)CC3	5.76
O=C(CC1)[C@]2([H])[C@@]31[C@@H](C)[C@H](O)[C@](C)(C=C)C[C@@H](OC(CN4CCN(C(NC5=CC=C(N)C=C5)=O)CC4)=O)[C@]2(C)[C@H](C)CC3	5.76
O=C(CC1)[C@]2([H])[C@@]31[C@@H](C)[C@H](O)[C@](C)(C=C)C[C@@H](OC(CS(S(C4=NC5=C(C=CC=C5)N4)=O)=O)=O)[C@]2(C)[C@H](C)CC3	5.76
O=C1CC[C@]2(CC[C@H]3C)[C@]1([H])[C@]3(C)[C@H](OC(CN4CCN(C(CN5CCCCC5)=O)CC4)=O)C[C@@](C)(C=C)[C@@H](O)[C@@H]2C	5.76
O=C(CC1)[C@]2([H])[C@@]31[C@@H](C)[C@H](O)[C@](C)(C=C)C[C@@H](OC(CS(S(C4=NC(N)=CC(N)=N4)=O)=O)=O)[C@]2(C)[C@H](C)CC3	5.75
O=C(CC1)[C@]2([H])[C@@]31[C@@H](C)[C@H](O)[C@](C)(C=C)C[C@@H](OC(CN4CCN(C(NC5=CC=CC=C5)=O)CC4)=O)[C@]2(C)[C@H](C)CC3	5.75
O=C(CC1)[C@]2([H])[C@@]31[C@@H](C)[C@H](O)[C@](C)(C=C)C[C@@H](OC(CSC4=NN=C(NC(N(C)C)=O)S4)=O)[C@]2(C)[C@H](C)CC3	5.75
O=C(CC1)[C@]2([H])[C@@]31[C@@H](C)[C@H](O)[C@](C)(C=C)C[C@@H](OC(CS[C@H]4CC[C@H](C(OCC)=O)C[C@H]4N)=O)[C@]2(C)[C@H](C)CC3	5.75
O=C(CC1)[C@]2([H])[C@@]31[C@@H](C)[C@H](O)[C@](C)(C=C)C[C@@H](OC(CS(S(C4=NN=C(N)S4)=O)=O)=O)[C@]2(C)[C@H](C)CC3	5.75
O=C1CC[C@]2(CC[C@H]3C)[C@]1([H])[C@]3(C)[C@H](OC(CN4CCN(C5=CC=CC(Cl)=C5)CC4)=O)C[C@@](C)(C=C)[C@@H](O)[C@@H]2C	5.75
O=C(CC1)[C@]2([H])[C@@]31[C@@H](C)[C@H](O)[C@](C)(C=C)C[C@@H](OC(CSC4=NN=C(C5=CC=C(F)C=C5)O4)=O)[C@]2(C)[C@H](C)CC3	5.75
O=C1CC[C@]2(CC[C@H]3C)[C@]1([H])[C@]3(C)[C@H](OC(CN4CCN(C5=CC=C(OC)C=C5)CC4)=O)C[C@@](C)(C=C)[C@@H](O)[C@@H]2C	5.74
O=C(CC1)[C@]2([H])[C@@]31[C@@H](C)[C@H](O)[C@](C)(C=C)C[C@@H](OC(CSC4=NN=C(C5=CC=CC=C5C)O4)=O)[C@]2(C)[C@H](C)CC3	5.74
O=C1CC[C@]2(CC[C@H]3C)[C@]1([H])[C@]3(C)[C@H](OC(CN4CCN(C(C5NCCC5)=O)CC4)=O)C[C@@](C)(C=C)[C@@H](O)[C@@H]2C	5.74
O=C(CC1)[C@]2([H])[C@@]31[C@@H](C)[C@H](O)[C@](C)(C=C)C[C@@H](OC(CSC4=CC(/C=N\OCC)=CN=C4)=O)[C@]2(C)[C@H](C)CC3	5.73
O=C(CC1)[C@]2([H])[C@@]31[C@@H](C)[C@H](O)[C@](C)(C=C)C[C@@H](OC(CN4CCN(C5=CC=C(O)C=C5)CC4)=O)[C@]2(C)[C@H](C)CC3	5.73
O=C(CC1)[C@]2([H])[C@@]31[C@@H](C)[C@H](O)[C@](C)(C=C)C[C@@H](OC(N(CC4)C(C5=C4C=CC([N+]([O-])=O)=C5)=O)=O)[C@]2(C)[C@H](C)CC3	5.73
O=C1CC[C@]2(CC[C@H]3C)[C@]1([H])[C@]3(C)[C@H](OC(CN4CCN(C5=CC=CC(C)=C5)CC4)=O)C[C@@](C)(C=C)[C@@H](O)[C@@H]2C	5.73
O=C(CC1)[C@]2([H])[C@@]31[C@@H](C)[C@H](O)[C@](C)(C=C)C[C@@H](OC(CS(S(C4=NC=CC=N4)=O)=O)=O)[C@]2(C)[C@H](C)CC3	5.73
O=C(CC1)[C@]2([H])[C@@]31[C@@H](C)[C@H](O)[C@](C)(C=C)C[C@@H](OC(CS(S(C4=CC=NC=C4)=O)=O)=O)[C@]2(C)[C@H](C)CC3	5.73
O=C(CC1)[C@]2([H])[C@@]31[C@@H](C)[C@H](O)[C@](C)(C=C)C[C@@H](OC(CN4N=NC(C5=CC=CC(C#N)=C5)=C4)=O)[C@]2(C)[C@H](C)CC3	5.72
O=C1CC[C@]2(CC[C@H]3C)[C@]1([H])[C@]3(C)[C@H](OC(CSC4=NC(NC=C5)=C5C(N)=N4)=O)C[C@@](C)(C=C)[C@@H](O)[C@@H]2C	5.72
O=C(CC1)[C@]2([H])[C@@]31[C@@H](C)[C@H](O)[C@](C)(C=C)C[C@@H](OC(CS(S(C4=NNC=N4)=O)=O)=O)[C@]2(C)[C@H](C)CC3	5.72
O=C(CC1)[C@]2([H])[C@@]31[C@@H](C)[C@H](O)[C@](C)(C=C)C[C@@H](OC(CS(SC4=NN=NN4C)=O)=O)[C@]2(C)[C@H](C)CC3	5.72
O=C(CC1)[C@]2([H])[C@@]31[C@@H](C)[C@H](O)[C@](C)(C=C)C[C@@H](OC(CN4N=NC(C5=CC=CC(N)=C5)=C4)=O)[C@]2(C)[C@H](C)CC3	5.72
O=C1CC[C@]2(CC[C@H]3C)[C@]1([H])[C@]3(C)[C@H](OC(CSC4=NC(OC)=CC(N)=N4)=O)C[C@@](C)(C=C)[C@@H](O)[C@@H]2C	5.71
O=C(CC1)[C@]2([H])[C@@]31[C@@H](C)[C@H](O)[C@](C)(C=C)C[C@@H](OC(CSC4=NC(N)=CC(OC)=N4)=O)[C@]2(C)[C@H](C)CC3	5.71
O=C(CC1)[C@]2([H])[C@@]31[C@@H](C)[C@H](O)[C@](C)(C=C)C[C@@H](OC(CSCC4=CC(C(O)=CN4)=O)=O)[C@]2(C)[C@H](C)CC3	5.71
O=C(CC1)[C@]2([H])[C@@]31[C@@H](C)[C@H](O)[C@](C)(C=C)C[C@@H](OC(CSC4=NC(C=CC(NC(C5CCCCC5)=O)=C6)=C6N4)=O)[C@]2(C)[C@H](C)CC3	5.71
O=C(CC1)[C@]2([H])[C@@]31[C@@H](C)[C@H](O)[C@](C)(C=C)C[C@@H](OC(CSCC4=CC(C(O)=C(CN5CCC(O)CC5)O4)=O)=O)[C@]2(C)[C@H](C)CC3	5.70
O=C(CC1)[C@]2([H])[C@@]31[C@@H](C)[C@H](O)[C@](C)(C=C)C[C@@H](OC(CSC4=CC(OC)=CN=C4)=O)[C@]2(C)[C@H](C)CC3	5.70
O=C(CC1)[C@]2([H])[C@@]31[C@@H](C)[C@H](O)[C@](C)(C=C)C[C@@H](OC(CSC4=CC(C(OC)=O)=CN=C4)=O)[C@]2(C)[C@H](C)CC3	5.70

O=C(CC1)[C@]2([H])[C@@]31[C@@H](C)[C@H](O)[C@](C)(C=C)C[C@@H](OC(CN4CCN(C(/C=C/C5=CC([N+]([O-])=O)=CC=C5)=O)CC4)=O)[C@]2(C)[C@H](C)CC3	5.70
O=C(CC1)[C@]2([H])[C@@]31[C@@H](C)[C@H](O)[C@](C)(C=C)C[C@@H](OC(CNCCC4=CC=C(C)C=C4)=O)[C@]2(C)[C@H](C)CC3	5.70
O=C(CC1)[C@]2([H])[C@@]31[C@@H](C)[C@H](O)[C@](C)(C=C)C[C@@H](OC(CNCCC4=C(C)C=CC=C4)=O)[C@]2(C)[C@H](C)CC3	5.70
O=C(CC1)[C@]2([H])[C@@]31[C@@H](C)[C@H](O)[C@](C)(C=C)C[C@@H](OC(CNCCC4=C(C)C=CC=C4)=O)[C@]2(C)[C@H](C)CC3	5.70
O=C(CC1)[C@]2([H])[C@@]31[C@@H](C)[C@H](O)[C@](C)(C=C)C[C@@H](OC(CNCCCC4=CC=CC=C4)=O)[C@]2(C)[C@H](C)CC3	5.70
O=C(CC1)[C@]2([H])[C@@]31[C@@H](C)[C@H](O)[C@](C)(C=C)C[C@@H](OC(CSC4=NC(C=CC(NC(C5=CC=CN5)=O)=C6)=C6N4)=O)[C@]2(C)[C@H](C)CC3	5.69
O=C(CC1)[C@]2([H])[C@@]31[C@@H](C)[C@H](O)[C@](C)(C=C)C[C@@H](OC(CSC4=CC=C(CO)N=C4)=O)[C@]2(C)[C@H](C)CC3	5.69
O=C(CC1)[C@]2([H])[C@@]31[C@@H](C)[C@H](O)[C@](C)(C=C)C[C@@H](OC(C[N+]4(CCCC(CO)C4)C)=O)[C@]2(C)[C@H](C)CC3	5.69
O=C(CC1)[C@]2([H])[C@@]31[C@@H](C)[C@H](O)[C@](C)(C=C)C[C@@H](OC(C[N+]4=CC=C(N)C=C4Cl)=O)[C@]2(C)[C@H](C)CC3	5.69
O=C(CC1)[C@]2([H])[C@@]31[C@@H](C)[C@H](O)[C@](C)(C=C)C[C@@H](OC(CSC4=CC(NC(CN5CCCCC5)=O)=CN=C4)=O)[C@]2(C)[C@H](C)CC3	5.69
O=C(CC1)[C@]2([H])[C@@]31[C@@H](C)[C@H](O)[C@](C)(C=C)C[C@@H](OC(CN4CCN(C(/C=C/C5=CC=C(Cl)C=C5)=O)CC4)=O)[C@]2(C)[C@H](C)CC3	5.69
O=C(CC1)[C@]2([H])[C@@]31[C@@H](C)[C@H](O)[C@](C)(C=C)C[C@@H](OC(CNCC4=CC=CC=C4F)=O)[C@]2(C)[C@H](C)CC3	5.69
O=C(CC1)[C@]2([H])[C@@]31[C@@H](C)[C@H](O)[C@](C)(C=C)C[C@@H](OC(CNCC4=CC=CC(F)=C4)=O)[C@]2(C)[C@H](C)CC3	5.69
O=C(CC1)[C@]2([H])[C@@]31[C@@H](C)[C@H](O)[C@](C)(C=C)C[C@@H](OC(CNCCS(=O)(C)=O)=O)[C@]2(C)[C@H](C)CC3	5.68
O=C(CC1)[C@]2([H])[C@@]31[C@@H](C)[C@H](O)[C@](C)(C=C)C[C@@H](OC(CNCCC4=CC=NC=C4)=O)[C@]2(C)[C@H](C)CC3	5.68
O=C(CC1)[C@]2([H])[C@@]31[C@@H](C)[C@H](O)[C@](C)(C=C)C[C@@H](OC(CNCCC4=CC=CC=C4)=O)[C@]2(C)[C@H](C)CC3	5.68
O=C(CC1)[C@]2([H])[C@@]31[C@@H](C)[C@H](O)[C@](C)(C=C)C[C@@H](OC(CSCC4=CC(C(O)=C(CN5CCCC5)O4)=O)=O)[C@]2(C)[C@H](C)CC3	5.68
O=C(CC1)[C@]2([H])[C@@]31[C@@H](C)[C@H](O)[C@](C)(C=C)C[C@@H](OC(C[N+]4=C(CCC5)C5=CC=C4)=O)[C@]2(C)[C@H](C)CC3	5.68
O=C(CC1)[C@]2([H])[C@@]31[C@@H](C)[C@H](O)[C@](C)(C=C)C[C@@H](OC(CSC4=NC(N)=NN4)=O)[C@]2(C)[C@H](C)CC3	5.68
O=C(CC1)[C@]2([H])[C@@]31[C@@H](C)[C@H](O)[C@](C)(C=C)C[C@@H](OC(CSC4=CC=C(N)N=C4)=O)[C@]2(C)[C@H](C)CC3	5.67
O=C1CC[C@]2(CC[C@H]3C)[C@]1([H])[C@]3(C)[C@H](OC(CSC4=NC(N(CC)CC)=CC(C)=N4)=O)C[C@@](C)(C=C)[C@@H](O)[C@@H]2C	6.09
O=C(CC1)[C@]2([H])[C@@]31[C@@H](C)[C@H](O)[C@](C)(C=C)C[C@@H](OC(CN4CCSCC4)=O)[C@]2(C)[C@H](C)CC3	5.67
O=C(CC1)[C@]2([H])[C@@]31[C@@H](C)[C@H](O)[C@](C)(C=C)C[C@@H](OC(CNCCSC)=O)[C@]2(C)[C@H](C)CC3	5.65
O=C(CC1)[C@]2([H])[C@@]31[C@@H](C)[C@H](O)[C@](C)(C=C)C[C@@H](OC(CN4CCOCC4)=O)[C@]2(C)[C@H](C)CC3	5.65
O=C(CC1)[C@]2([H])[C@@]31[C@@H](C)[C@H](O)[C@](C)(C=C)C[C@@H](OC(CN4CCNCC4)=O)[C@]2(C)[C@H](C)CC3	5.65
O=C(CC1)[C@]2([H])[C@@]31[C@@H](C)[C@H](O)[C@](C)(C=C)C[C@@H](OC(C[N+]4=CC=CC=C4)=O)[C@]2(C)[C@H](C)CC3	5.64
O=C(CC1)[C@]2([H])[C@@]31[C@@H](C)[C@H](O)[C@](C)(C=C)C[C@@H](OC(CN4CCN(C(/C=C/C5=CC=CC=C5Br)=O)CC4)=O)[C@]2(C)[C@H](C)CC3	5.64
O=C(CC1)[C@]2([H])[C@@]31[C@@H](C)[C@H](O)[C@](C)(C=C)C[C@@H](OC(CN4CCN(C(/C=C/C5=CC=C(Br)C=C5)=O)CC4)=O)[C@]2(C)[C@H](C)CC3	5.64
O=C(CC1)[C@]2([H])[C@@]31[C@@H](C)[C@H](O)[C@](C)(C=C)C[C@@H](OC(CN4CCN(C(/C=C/C5=CC=C(C(F)(F)F)C=C5)=O)CC4)=O)[C@]2(C)[C@H](C)CC3	5.63
O=C(CC1)[C@]2([H])[C@@]31[C@@H](C)[C@H](O)[C@](C)(C=C)C[C@@H](OC(CNCCO)=O)[C@]2(C)[C@H](C)CC3	5.62
O=C(CC1)[C@]2([H])[C@@]31[C@@H](C)[C@H](O)[C@](C)(C=C)C[C@@H](OC(CSC4=CC=C([N+]([O-])=O)C=N4)=O)[C@]2(C)[C@H](C)CC3	5.62
O=C(CC1)[C@]2([H])[C@@]31[C@@H](C)[C@H](O)[C@](C)(C=C)C[C@@H](OC(CSC4=C(NC(CN5N=NC(CN6CCN(C7=CC=CC(Cl)=C7)CC6)=C5)=O)C=CC=C4)=O)[C@]2(C)[C@H](C)CC3	5.60
O=C(CC1)[C@]2([H])[C@@]31[C@@H](C)[C@H](O)[C@](C)(C=C)C[C@@H](OC(CSC4=C(C#N)C=CC=N4)=O)[C@]2(C)[C@H](C)CC3	5.60
O=C(CC1)[C@]2([H])[C@@]31[C@@H](C)[C@H](O)[C@](C)(C=C)C[C@@H](OC(CSC4=C(NC(CN5N=NC(CN6CCN(C7=CC=C(O)C=C7)CC6)=C5)=O)C=CC=C4)=O)[C@]2(C)[C@H](C)CC3	5.59
O=C(CC1)[C@]2([H])[C@@]31[C@@H](C)[C@H](O)[C@](C)(C=C)C[C@@H](OC(CSC4CCN(C(CCN5C=NC6=C5N=C(N)N=C6N7CCC(NC)CC7)=O)CC4)=O)[C@]2(C)[C@H](C)CC3	5.59
O=C(CC1)[C@]2([H])[C@@]31[C@@H](C)[C@H](O)[C@](C)(C=C)C[C@@H](OC(CN4CCN(C(CN5N=NC(CN6CCN(C7=CC=CC=C7[N+]([O-])=O)CC6)=C5)=O)CC4)=O)[C@]2(C)[C@H](C)CC3	5.59
O=C(CC1)[C@]2([H])[C@@]31[C@@H](C)[C@H](O)[C@](C)(C=C)C[C@@H](OC(CN4CCN(C(CN5N=NC(CN6CCN(C7=CC=CC([N+]([O-])=O)=C7)CC6)=C5)=O)CC4)=O)[C@]2(C)[C@H](C)CC3	5.59
O=C(CC1)[C@]2([H])[C@@]31[C@@H](C)[C@H](O)[C@](C)(CC)C[C@@H](OC(CS(C(CC4)CCN4C(CCN5C=NC6=C5N=CN=C6N7CCC(C7)N)=O)(=O)=O)=O)[C@]2(C)[C@H](C)CC3	5.59
O=C(CC1)[C@]2([H])[C@@]31[C@@H](C)[C@H](O)[C@](C)(C=C)C[C@@H](OC(CSCC(C4)OCCN4C(CCN5C=NC6=C5N=C(N)N=C6N7CCNCC7)=O)=O)[C@]2(C)[C@H](C)CC3	5.58
O=C(CC1)[C@]2([H])[C@@]31[C@@H](C)[C@H](O)[C@](C)(C=C)C[C@@H](OC(CSC4CCN(C(CCN5C=NC6=C5N=C(N)N=C6N7CC(CN)CC7)=O)CC4)=O)[C@]2(C)[C@H](C)CC3	5.58
O=C(CC1)[C@]2([H])[C@@]31[C@@H](C)[C@H](O)[C@](C)(C=C)C[C@@H](OC(CN4CCN(C(CN5N=NC(CN6CCN(C7=C(C)C=CC=C7)CC6)=C5)=O)CC4)=O)[C@]2(C)[C@H](C)CC3	5.57
O=C(CC1)[C@]2([H])[C@@]31[C@@H](C)[C@H](O)[C@](C)(C=C)C[C@@H](OC(CS[C@@H](C4)CCN4C(CCN5C=NC6=C5N=C(N)N=C6N7CCNCC7)=O)=O)[C@]2(C)[C@H](C)CC3	5.57
O=C1CC[C@]2(CC[C@H]3C)[C@]1([H])[C@]3(C)[C@H](OC(CN4CCN(C(C(CC5=CNC6=C5C=CC=C6)NC(OC(C)(C)C)=O)=O)CC4)=O)C[C@@](C)(C=C)[C@@H](O)[C@@H]2C	5.56
O=C(CC1)[C@]2([H])[C@@]31[C@@H](C)[C@H](O)[C@](C)(C=C)C[C@@H](OC(CSC4=C(NC(CN5CCN(C6=CC=C([N+]([O-])=O)C=C6)CC5)=O)C=CC=C4)=O)[C@]2(C)[C@H](C)CC3	5.56
O=C1CC[C@]2(CC[C@H]3C)[C@]1([H])[C@]3(C)[C@H](OC(CSC4=NC(NC=C5)=C5C(NC(CN6CCC(C7=CC=CC=C7)CC6)=O)=N4)=O)C[C@@](C)(C=C)[C@@H](O)[C@@H]2C	5.56
O=C(CC1)[C@]2([H])[C@@]31[C@@H](C)[C@H](O)[C@](C)(C=C)C[C@@H](OC(CSC(C4)CN4C(CCN5C=NC6=C5N=C(N)N=C6N7CCNCC7)=O)=O)[C@]2(C)[C@H](C)CC3	5.56
O=C(CC1)[C@]2([H])[C@@]31[C@@H](C)[C@H](O)[C@](C)(C=C)C[C@@H](OC(CNC(CC4)CCN4C(CCN5C=NC6=C5N=CN=C6N7CCC(C7)N)=O)=O)[C@]2(C)[C@H](C)CC3	5.56
O=C(CC1)[C@]2([H])[C@@]31[C@@H](C)[C@H](O)[C@](C)(C=C)C[C@@H](OC(CSC4=C(NC(CN5CCN(C6=CC=C(OC)C=C6)CC5)=O)C=CC=C4)=O)[C@]2(C)[C@H](C)CC3	5.56
O=C(CC1)[C@]2([H])[C@@]31[C@@H](C)[C@H](O)[C@](C)(C=C)C[C@@H](OC(CN4CCN(C5=CC=C(C6CC(C7=CC=CC=C7[N+]([O-])=O)=NN6)C=C5)CC4)=O)[C@]2(C)[C@H](C)CC3	5.55
O=C(CC1)[C@]2([H])[C@@]31[C@@H](C)[C@H](O)[C@](C)(C=C)C[C@@H](OC(CSC4=C(NC(CN5CCN(C6=CC=CC=C6F)CC5)=O)C=CC=C4)=O)[C@]2(C)[C@H](C)CC3	5.55
O=C(CC1)[C@]2([H])[C@@]31[C@@H](C)[C@H](O)[C@](C)(C=C)C[C@@H](OC(CSC4=C(NC(CN5CCN(C6=C(C)C=CC=C6)CC5)=O)C=CC=C4)=O)[C@]2(C)[C@H](C)CC3	5.55

O=C(N(CC1)CCN1CC(O[C@@H]([C@]2(C)[C@H](C)CC3)C[C@@](C)(C=C)[C@@H](O)[C@H](C)[C@@]43[C@@]2([H])C(CC4)=O)=O)[C@H](CC5=CC=CC=C5)NC(OC(C)(C)C)=O	5.54
O=C(CC1)[C@]2([H])[C@@]31[C@@H](C)[C@H](O)[C@](C)(C=C)C[C@@H](OC(CN4N=NC(C5=CC=CC(CC6=CC=CC(CN(CC)CC)=C6)=C5)=C4)=O)[C@]2(C)[C@H](C)CC3	5.53
O=C1CC[C@]2(CC[C@H]3C)[C@]1([H])[C@]3(C)[C@H](OC(CN4CCN(C(NC5=CC=CC(NC(OC(C)(C)C)=O)=C5)=O)CC4)=O)C[C@@](C)(C=C)[C@@H](O)[C@@H]2C	5.53
O=C(CC1)[C@]2([H])[C@@]31[C@@H](C)[C@H](O)[C@](C)(C=C)C[C@@H](OC(N4CC5=C(C=CC=C5OCCCN6C=NC(C7=CC=CN=C7)=C6)C4=O)=O)[C@]2(C)[C@H](C)CC3	5.53
O=C(CC1)[C@]2([H])[C@@]31[C@@H](C)[C@H](O)[C@](C)(C=C)C[C@@H](OC(N4C(C(C=C(NC(CCN5CCN(CCO)CC5)=O)C=C6)=C6C4)=O)=O)[C@]2(C)[C@H](C)CC3	5.53
O=C(CC1)[C@]2([H])[C@@]31[C@@H](C)[C@H](O)[C@](C)(C=C)C[C@@H](OC(CN4CCN(C(CN5CCN(C6=CC=C(O)C=C6)CC5)=O)CC4)=O)[C@]2(C)[C@H](C)CC3	5.52
CC1C23C(C(CC3)=O)C(C(CC(C=C)(C)C1O)OC(CN4CCN(C(CN5CCN(C6=CC=C(O)C=C6)CC5)=O)CC4)=O)(C(CC2)C)C	5.52
O=C(CC1)[C@]2([H])[C@@]31[C@@H](C)[C@H](O)[C@](C)(C=C)C[C@@H](OC(CN4N=NC(CN5CCN(C6=CC=CC([N+]([O-])=O)=C6)CC5)=C4)=O)[C@]2(C)[C@H](C)CC3	5.51
O=C(CC1)[C@]2([H])[C@@]31[C@@H](C)[C@H](O)[C@](C)(C=C)C[C@@H](OC(CSC4=NN(C(C5=CC=CC=C5C(F)(F)F)=O)C(N)=N4)=O)[C@]2(C)[C@H](C)CC3	5.51
O=C1CC[C@]2(CC[C@H]3C)[C@]1([H])[C@]3(C)[C@H](OC(CN4CCN(C([C@H]([C@@H](C)O)NC(OC(C)(C)C)=O)=O)CC4)=O)C[C@@](C)(C=C)[C@@H](O)[C@@H]2C	5.51
O=C(CC1)[C@]2([H])[C@@]31[C@@H](C)[C@H](O)[C@](C)(C=C)C[C@@H](OC(CSC4=NN=C(C)N4/N=C/C5=CC=CC(C(F)(F)F)=C5)=O)[C@]2(C)[C@H](C)CC3	5.51
O=C(CC1)[C@]2([H])[C@@]31[C@@H](C)[C@H](O)[C@](C)(C=C)C[C@@H](OC(CSC4=NN=C(C)N4/N=C/C5=CC=C(C(F)(F)F)C=C5)=O)[C@]2(C)[C@H](C)CC3	5.51
O=C(N(CC1)CCN1CC(O[C@@H]([C@]2(C)[C@H](C)CC3)C[C@@](C)(C=C)[C@@H](O)[C@H](C)[C@@]43[C@@]2([H])C(CC4)=O)=O)[C@H](C(C)C)NC(OC(C)(C)C)=O	5.51
O=C1CC[C@]2(CC[C@H]3C)[C@]1([H])[C@]3(C)[C@H](OC(CN4CCN(C(NC5=CC=CC(C(F)(F)F)=C5)=O)CC4)=O)C[C@@](C)(C=C)[C@@H](O)[C@@H]2C	5.50
O=C(CC1)[C@]2([H])[C@@]31[C@@H](C)[C@H](O)[C@](C)(C=C)C[C@@H](OC(CSC4=NN=C(NC(C5=CC=CC=C5Cl)=O)S4)=O)[C@]2(C)[C@H](C)CC3	5.50
O=C(CC1)[C@]2([H])[C@@]31[C@@H](C)[C@H](O)[C@](C)(C=C)C[C@@H](OC(CSC4=NN=C(NC(C5=CC=CC(Cl)=C5)=O)S4)=O)[C@]2(C)[C@H](C)CC3	5.50
O=C(CC1)[C@]2([H])[C@@]31[C@@H](C)[C@H](O)[C@](C)(C=C)C[C@@H](OC(CSC4=NN=C(NC(C5=CC=C(OC)C=C5)=O)S4)=O)[C@]2(C)[C@H](C)CC3	5.50
O=C(CC1)[C@]2([H])[C@@]31[C@@H](C)[C@H](O)[C@](C)(C=C)C[C@@H](OC(CN4N=NC(C5=CC=CC=C5CC6=CC=C(OC)C=C6)=C4)=O)[C@]2(C)[C@H](C)CC3	5.50
O=C1CC[C@]2(CC[C@H]3C)[C@]1([H])[C@]3(C)[C@H](OC(CSC4=NC(OCCCC)=CC(C)=N4)=O)C[C@@](C)(C=C)[C@@H](O)[C@@H]2C	5.49
O=C(CC1)[C@]2([H])[C@@]31[C@@H](C)[C@H](O)[C@](C)(C=C)C[C@@H](OC(CN4CCN(C(NCCC5=CC=CC(OC)=C5)=O)CC4)=O)[C@]2(C)[C@H](C)CC3	5.49
O=C(CC1)[C@]2([H])[C@@]31[C@@H](C)[C@H](O)[C@](C)(C=C)C[C@@H](OC(CN4CCN(C(NC5=CC=CC(NC(C)=O)=C5)=O)CC4)=O)[C@]2(C)[C@H](C)CC3	5.49
O=C(CC1)[C@]2([H])[C@@]31[C@@H](C)[C@H](O)[C@](C)(C=C)C[C@@H](OC(CN4N=NC(CN5CCN(C6=CC=CC=C6F)CC5)=C4)=O)[C@]2(C)[C@H](C)CC3	5.49
O=C(CC1)[C@]2([H])[C@@]31[C@@H](C)[C@H](O)[C@](C)(C=C)C[C@@H](OC(CN4N=NC(C5=CC=CC(CN6CCN(C)CC6)=C5)=C4)=O)[C@]2(C)[C@H](C)CC3	5.49
O=C1CC[C@]2(CC[C@H]3C)[C@]1([H])[C@]3(C)[C@H](OC(CN4CCN(C(NC5=C(C=CC=N6)C6=CC=C5)=O)CC4)=O)C[C@@](C)(C=C)[C@@H](O)[C@@H]2C	5.49
O=C(CC1)[C@]2([H])[C@@]31[C@@H](C)[C@H](O)[C@](C)(C=C)C[C@@H](OC(CSC4=NN=C(NC(C5=CC=C(N)C=C5)=O)S4)=O)[C@]2(C)[C@H](C)CC3	5.49
O=C1CC[C@]2(CC[C@H]3C)[C@]1([H])[C@]3(C)[C@H](OC(CN4CCN(C(NC5=CC=C([N+]([O-])=O)C=C5)=O)CC4)=O)C[C@@](C)(C=C)[C@@H](O)[C@@H]2C	5.48
O=C1CC[C@]2(CC[C@H]3C)[C@]1([H])[C@]3(C)[C@H](OC(CN4CCN(C(NCC5=CC=C(OC)C=C5)=O)CC4)=O)C[C@@](C)(C=C)[C@@H](O)[C@@H]2C	5.48
O=C(CC1)[C@]2([H])[C@@]31[C@@H](C)[C@H](O)[C@](C)(C=C)C[C@@H](OC(CSC(C)(C)CNC(C4=CC(C=CC=C5)=C5N4)=O)=O)[C@]2(C)[C@H](C)CC3	5.48
O=C(CC1)[C@]2([H])[C@@]31[C@@H](C)[C@H](O)[C@](C)(C=C)C[C@@H](OC(CSC(C)(C)CNC(CN(CCC)CCC)=O)=O)[C@]2(C)[C@H](C)CC3	5.48
O=C(CC1)[C@]2([H])[C@@]31[C@@H](C)[C@H](O)[C@](C)(C=C)C[C@@H](OC(CS(S(C4=NC5=C(C=CC(OC)=C5)N4)=O)=O)=O)[C@]2(C)[C@H](C)CC3	5.48
O=C(CC1)[C@]2([H])[C@@]31[C@@H](C)[C@H](O)[C@](C)(C=C)C[C@@H](OC(CN4N=NC(CCCN5C(N=CN=C6N)=C6N=C5)=C4)=O)[C@]2(C)[C@H](C)CC3	5.48
O=C(CC1)[C@]2([H])[C@@]31[C@@H](C)[C@H](O)[C@](C)(C=C)C[C@@H](OC(CN4CCN(C(NCCN5CCOCC5)=O)CC4)=O)[C@]2(C)[C@H](C)CC3	5.48
O=C(CC1)[C@]2([H])[C@@]31[C@@H](C)[C@H](O)[C@](C)(C=C)C[C@@H](OC(CSC4=NC(N)=CC(NC([C@H](C(C)C)N)=O)=N4)=O)[C@]2(C)[C@H](C)CC3	5.48
O=C1CC[C@]2(CC[C@H]3C)[C@]1([H])[C@]3(C)[C@H](OC(CSC4=NC(O)=CC(NC([C@H](C)C(C)C)=O)=N4)=O)C[C@@](C)(C=C)[C@@H](O)[C@@H]2C	5.48
O=C(CC1)[C@]2([H])[C@@]31[C@@H](C)[C@H](O)[C@](C)(C=C)C[C@@H](OC(CSC(C)(C)CNC(C4=CC(OC)=CC=C4)=O)=O)[C@]2(C)[C@H](C)CC3	5.48
O=C(CC1)[C@]2([H])[C@@]31[C@@H](C)[C@H](O)[C@](C)(C=C)C[C@@H](OC(CSC(C)(C)CNC([C@@H](C)C4=CC=CC=C4)=O)=O)[C@]2(C)[C@H](C)CC3	5.48
O=C1CC[C@]2(CC[C@H]3C)[C@]1([H])[C@]3(C)[C@H](OC(CN4CCN(C(NC5=CC=C(OC)C=C5)=O)CC4)=O)C[C@@](C)(C=C)[C@@H](O)[C@@H]2C	5.47
O=C(CC1)[C@]2([H])[C@@]31[C@@H](C)[C@H](O)[C@](C)(C=C)C[C@@H](OC(CN4CCN(C(NCC5=CC=C(C)C=C5)=O)CC4)=O)[C@]2(C)[C@H](C)CC3	5.47
O=C1CC[C@]2(CC[C@H]3C)[C@]1([H])[C@]3(C)[C@H](OC(CN4CCN(C(NCC5=CC=C(C)C=C5)=O)CC4)=O)C[C@@](C)(C=C)[C@@H](O)[C@@H]2C	5.47
O=C(CC1)[C@]2([H])[C@@]31[C@@H](C)[C@H](O)[C@](C)(C=C)C[C@@H](OC(CSC(C)(C)CNC(CN4CCCCC4)=O)=O)[C@]2(C)[C@H](C)CC3	5.47
O=C(CC1)[C@]2([H])[C@@]31[C@@H](C)[C@H](O)[C@](C)(C=C)C[C@@H](OC(CN4N=NC(C5=CC=CC(CN(CC)CC)=C5)=C4)=O)[C@]2(C)[C@H](C)CC3	5.47
O=C(CC1)[C@]2([H])[C@@]31[C@@H](C)[C@H](O)[C@](C)(C=C)C[C@@H](OC(CN4CCN(C(/C=C/C5=C(C)C=CC=C5)=O)CC4)=O)[C@]2(C)[C@H](C)CC3	5.47
O=C(CC1)[C@]2([H])[C@@]31[C@@H](C)[C@H](O)[C@](C)(C=C)C[C@@H](OC(N4C(C(C=CC(NC(C(F)(F)F)=O)=C5)=C5C4)=O)=O)[C@]2(C)[C@H](C)CC3	5.47
O=C(CC1)[C@]2([H])[C@@]31[C@@H](C)[C@H](O)[C@](C)(C=C)C[C@@H](OC(CSC(C)(C)CNC(C4=CC(N)=CC=C4)=O)=O)[C@]2(C)[C@H](C)CC3	5.47
O=C(CC1)[C@]2([H])[C@@]31[C@@H](C)[C@H](O)[C@](C)(C=C)C[C@@H](OC(N4C(C(C=CC(NCC5=CC=CC=C5)=C6)=C6C4)=O)=O)[C@]2(C)[C@H](C)CC3	5.47
O=C(CC1)[C@]2([H])[C@@]31[C@@H](C)[C@H](O)[C@](C)(C=C)C[C@@H](OC(CS(SC4=NN=NN4CCN(C)C)=O)=O)[C@]2(C)[C@H](C)CC3	5.46
O=C(CC1)[C@]2([H])[C@@]31[C@@H](C)[C@H](O)[C@](C)(C=C)C[C@@H](OC(CN4CCN(C(N(C)C5=CC=CC=C5)=O)CC4)=O)[C@]2(C)[C@H](C)CC3	5.46
O=C1CC[C@]2(CC[C@H]3C)[C@]1([H])[C@]3(C)[C@H](OC(CN4CCN(C([C@H](CCSC)N)=O)CC4)=O)C[C@@](C)(C=C)[C@@H](O)[C@@H]2C	5.46
O=C(CC1)[C@]2([H])[C@@]31[C@@H](C)[C@H](O)[C@](C)(C=C)C[C@@H](OC(CS(S(C4=NC5=C(C=CC=C5)O4)=O)=O)=O)[C@]2(C)[C@H](C)CC3	5.46
O=C1CC[C@]2(CC[C@H]3C)[C@]1([H])[C@]3(C)[C@H](OC(CN4CCN(C(CN5CCOCC5)=O)CC4)=O)C[C@@](C)(C=C)[C@@H](O)[C@@H]2C	5.46
O=C(CC1)[C@]2([H])[C@@]31[C@@H](C)[C@H](O)[C@](C)(C=C)C[C@@H](OC(CSC(C)(C)CNC(OCC(C)C)=O)=O)[C@]2(C)[C@H](C)CC3	5.45
O=C1CC[C@]2(CC[C@H]3C)[C@]1([H])[C@]3(C)[C@H](OC(CN4CCN(C(NC5=CC=CC=C5)=O)CC4)=O)C[C@@](C)(C=C)[C@@H](O)[C@@H]2C	5.45
O=C1CC[C@]2(CC[C@H]3C)[C@]1([H])[C@]3(C)[C@H](OC(CSC4=NC(OC(C)C)=CC(C)=N4)=O)C[C@@](C)(C=C)[C@@H](O)[C@@H]2C	6.07
O=C(CC1)[C@]2([H])[C@@]31[C@@H](C)[C@H](O)[C@](C)(C=C)C[C@@H](OC(CSC(C)(C)CNC(CNC(C)C)=O)=O)[C@]2(C)[C@H](C)CC3	5.45
O=C(CC1)[C@]2([H])[C@@]31[C@@H](C)[C@H](O)[C@](C)(C=C)C[C@@H](OC(N4C(C(C=CC(N5CCCCC5)=C6)=C6C4)=O)=O)[C@]2(C)[C@H](C)CC3	5.45
O=C1CC[C@]2(CC[C@H]3C)[C@]1([H])[C@]3(C)[C@H](OC(CN4CCN(C5=CC=CC=C5Cl)CC4)=O)C[C@@](C)(C=C)[C@@H](O)[C@@H]2C	5.44
O=C1CC[C@]2(CC[C@H]3C)[C@]1([H])[C@]3(C)[C@H](OC(CN4CCN(C5=CC=C(Cl)C=C5)CC4)=O)C[C@@](C)(C=C)[C@@H](O)[C@@H]2C	5.44
O=C(CC1)[C@]2([H])[C@@]31[C@@H](C)[C@H](O)[C@](C)(C=C)C[C@@H](OC(CS(S(C4=NN=CS4)=O)=O)=O)[C@]2(C)[C@H](C)CC3	5.43
O=C(CC1)[C@]2([H])[C@@]31[C@@H](C)[C@H](O)[C@](C)(C=C)C[C@@H](OC(CSC4=NN=C(C5=CC=CN=C5)O4)=O)[C@]2(C)[C@H](C)CC3	5.43
O=C(CC1)[C@]2([H])[C@@]31[C@@H](C)[C@H](O)[C@](C)(C=C)C[C@@H](OC(CN4N=NC(C5=CC=CC(CN)=C5)=C4)=O)[C@]2(C)[C@H](C)CC3	5.43
O=C(CC1)[C@]2([H])[C@@]31[C@@H](C)[C@H](O)[C@](C)(C=C)C[C@@H](OC(CN4N=NC(C5=CC=C(CN)C=C5)=C4)=O)[C@]2(C)[C@H](C)CC3	5.43
O=C(CC1)[C@]2([H])[C@@]31[C@@H](C)[C@H](O)[C@](C)(C=C)C[C@@H](OC(CS(SC4=NN=NN4C)=O)=O)[C@]2(C)[C@H](C)CC3	5.42

O=C(CC1)[C@]2([H])[C@@]31[C@@H](C)[C@H](O)[C@](C)(C=C)C[C@@H](OC(N(CC4)C(C5=C4C=C(OC)C=C5)=O)=O)[C@]2(C)[C@H](C)CC3	5.42
O=C1CC[C@]2(CC[C@H]3C)[C@]1([H])[C@]3(C)[C@H](OC(CN4CCN(C([C@H](C)N)=O)CC4)=O)C[C@@](C)(C=C)[C@@H](O)[C@@H]2C	5.41
O=C1CC[C@]2(CC[C@H]3C)[C@]1([H])[C@]3(C)[C@H](OC(CSC4=NC(N)=CC(N4C)=O)=O)C[C@@](C)(C=C)[C@@H](O)[C@@H]2C	5.41
O=C(CC1)[C@]2([H])[C@@]31[C@@H](C)[C@H](O)[C@](C)(C=C)C[C@@H](OC(CSC(N4C)=NC(N)=CC4=O)=O)[C@]2(C)[C@H](C)CC3	5.41
O=C(CC1)[C@]2([H])[C@@]31[C@@H](C)[C@H](O)[C@](C)(C=C)C[C@@H](OC(N4C(C(C=CC=C5OC)=C5C4)=O)=O)[C@]2(C)[C@H](C)CC3	5.41
O=C(CC1)[C@]2([H])[C@@]31[C@@H](C)[C@H](O)[C@](C)(C=C)C[C@@H](OC(CN4N=NC(C5=CC=CC=C5)=C4)=O)[C@]2(C)[C@H](C)CC3	5.40
O=C1CC[C@]2(CC[C@H]3C)[C@]1([H])[C@]3(C)[C@H](OC(CN4CCN(C(CN)=O)CC4)=O)C[C@@](C)(C=C)[C@@H](O)[C@@H]2C	5.40
O=C(CC1)[C@]2([H])[C@@]31[C@@H](C)[C@H](O)[C@](C)(C=C)C[C@@H](OC(CNCCC4=CC=C(O)C=C4)=O)[C@]2(C)[C@H](C)CC3	5.40
O=C(CC1)[C@]2([H])[C@@]31[C@@H](C)[C@H](O)[C@](C)(C=C)C[C@@H](OC(CN4CCN(C)CC4)=O)[C@]2(C)[C@H](C)CC3	5.36
O=C1CC[C@]2(CC[C@H]3C)[C@]1([H])[C@]3(C)[C@H](OC(CN4CCN(C)CC4)=O)C[C@@](C)(C=C)[C@@H](O)[C@@H]2C	5.36
CC1C23C(C(CC3)=O)C(C(CC(C=C)(C)C1O)OC(CN4CCNCC4)=O)(C(CC2)C)C	5.35
O=C(CC1)[C@]2([H])[C@@]31[C@@H](C)[C@H](O)[C@](C)(C=C)C[C@@H](OC(C[N+]4=CN(C)C=C4)=O)[C@]2(C)[C@H](C)CC3	5.35
O=C(CC1)[C@]2([H])[C@@]31[C@@H](C)[C@H](O)[C@](C)(C=C)C[C@@H](OC(CNCC(C)C)=O)[C@]2(C)[C@H](C)CC3	5.34
O=C(CC1)[C@]2([H])[C@@]31[C@@H](C)[C@H](O)[C@](C)(C=C)C[C@@H](OC(CNCCCC)=O)[C@]2(C)[C@H](C)CC3	5.34
O=C(CC1)[C@]2([H])[C@@]31[C@@H](C)[C@H](O)[C@](C)(C=C)C[C@@H](OC(CN4CCCC4)=O)[C@]2(C)[C@H](C)CC3	5.33
O=C(CC1)[C@]2([H])[C@@]31[C@@H](C)[C@H](O)[C@](C)(C=C)C[C@@H](OC(CN4CCN(C(/C=C/C5=CC=C(OC)C=C5)=O)CC4)=O)[C@]2(C)[C@H](C)CC3	5.31
O=C(CC1)[C@]2([H])[C@@]31[C@@H](C)[C@H](O)[C@](C)(C=C)C[C@@H](OC(CSC4CCN(C(CCN5C=NC6=C5N=C(N)N=C6N7CC(CNC)CC7)=O)CC4)=O)[C@]2(C)[C@H](C)CC3	5.29
O=C(CC1)[C@]2([H])[C@@]31[C@@H](C)[C@H](O)[C@](C)(C=C)C[C@@H](OC(CN4CCN(C(/C=C/C5=CC=CC=C5)=O)CC4)=O)[C@]2(C)[C@H](C)CC3	5.28
O=C(CC1)[C@]2([H])[C@@]31[C@@H](C)[C@H](O)[C@](C)(C=C)C[C@@H](OC(CN4CCN(C(CN5N=NC(CN6CCN(C7=CC=CC=C7OC)CC6)=C5)=O)CC4)=O)[C@]2(C)[C@H](C)CC3	5.28
O=C(CC1)[C@]2([H])[C@@]31[C@@H](C)[C@H](O)[C@](C)(C=C)C[C@@H](OC(CN4CCN(C(CN5N=NC(CN6CCN(C7=CC=CC(OC)=C7)CC6)=C5)=O)CC4)=O)[C@]2(C)[C@H](C)CC3	5.28
O=C(CC1)[C@]2([H])[C@@]31[C@@H](C)[C@H](O)[C@](C)(C=C)C[C@@H](OC(CN4CCN(C(CN5N=NC(CN6CCN(C7=CC=C(OC)C=C7)CC6)=C5)=O)CC4)=O)[C@]2(C)[C@H](C)CC3	5.28
O=C(CC1)[C@]2([H])[C@@]31[C@@H](C)[C@H](O)[C@](C)(C=C)C[C@@H](OC(CN4CCN(C(CN5N=NC(CN6CCN(C7=CC=CC(F)=C7)CC6)=C5)=O)CC4)=O)[C@]2(C)[C@H](C)CC3	5.27
O=C(CC1)[C@]2([H])[C@@]31[C@@H](C)[C@H](O)[C@](C)(C=C)C[C@@H](OC(CN4CCN(C(CN5N=NC(CN6CCN(C7=CC=C(F)C=C7)CC6)=C5)=O)CC4)=O)[C@]2(C)[C@H](C)CC3	5.27
O=C(CC1)[C@]2([H])[C@@]31[C@@H](C)[C@H](O)[C@](C)(C=C)C[C@@H](OC(CN4CCN(C(CN5N=NC(CN6CCN(C7=CC=CC(O)=C7)CC6)=C5)=O)CC4)=O)[C@]2(C)[C@H](C)CC3	5.27
O=C(CC1)[C@]2([H])[C@@]31[C@@H](C)[C@H](O)[C@](C)(C=C)C[C@@H](OC(CN4CCN(C(CN5N=NC(CN6CCN(C7=CC(C)=CC=C7)CC6)=C5)=O)CC4)=O)[C@]2(C)[C@H](C)CC3	5.27
O=C(CC1)[C@]2([H])[C@@]31[C@@H](C)[C@H](O)[C@](C)(C=C)C[C@@H](OC(CN4CCN(C(CN5N=NC(CN6CCN(N7CCCCC7)CC6)=C5)=O)CC4)=O)[C@]2(C)[C@H](C)CC3	5.27
O=C(CC1)[C@]2([H])[C@@]31[C@@H](C)[C@H](O)[C@](C)(C=C)C[C@@H](OC(CSCC4=CSC(NC(CN5CCN(C(OC(C)(C)C)=O)CC5)=O)=N4)=O)[C@]2(C)[C@H](C)CC3	5.26
O=C(CC1)[C@]2([H])[C@@]31[C@@H](C)[C@H](O)[C@](C)(C=C)C[C@@H](OC(CSC4=C(NC(CN5CCN(C6=C([N+]([O-])=O)C=CC=C6)CC5)=O)C=CC=C4)=O)[C@]2(C)[C@H](C)CC3	5.26
O=C(CC1)[C@]2([H])[C@@]31[C@@H](C)[C@H](O)[C@](C)(C=C)C[C@@H](OC(CSC4=C(NC(CN5CCN(C6=CC=CC([N+]([O-])=O)=C6)CC5)=O)C=CC=C4)=O)[C@]2(C)[C@H](C)CC3	5.26
O=C(CC1)[C@]2([H])[C@@]31[C@@H](C)[C@H](O)[C@](C)(C=C)C[C@@H](OC(CN4CCN(C(CN5N=NC(CN6CCN(C7=CC=CC=C7)CC6)=C5)=O)CC4)=O)[C@]2(C)[C@H](C)CC3	5.26
O=C(CC1)[C@]2([H])[C@@]31[C@@H](C)[C@H](O)[C@](C)(C=C)C[C@@H](OC(CN4CCN(C(CN5N=NC(CN6CCN(N(CC)CC)CC6)=C5)=O)CC4)=O)[C@]2(C)[C@H](C)CC3	5.26
O=C(CC1)[C@]2([H])[C@@]31[C@@H](C)[C@H](O)[C@](C)(C=C)C[C@@H](OC(CN4CCN(C(CN5N=NC(CN6CCN(N7CCCC7)CC6)=C5)=O)CC4)=O)[C@]2(C)[C@H](C)CC3	5.26
O=C1CC[C@]2(CC[C@H]3C)[C@]1([H])[C@]3(C)[C@H](OC(CN4CCN(C(NCC5=CC=C(NC(CN6CCOCC6)=O)C=C5)=O)CC4)=O)C[C@@](C)(C=C)[C@@H](O)[C@@H]2C	5.26
O=C(CC1)[C@]2([H])[C@@]31[C@@H](C)[C@H](O)[C@](C)(C=C)C[C@@H](OC(CSC4=C(NC(CN5CCN(C6=CC(OC)=CC=C6)CC5)=O)C=CC=C4)=O)[C@]2(C)[C@H](C)CC3	5.25
O=C(CC1)[C@]2([H])[C@@]31[C@@H](C)[C@H](O)[C@](C)(C=C)C[C@@H](OC(CSCC4=CC(C(O)=CN4C)=O)=O)[C@]2(C)[C@H](C)CC3	5.25
O=C(CC1)[C@]2([H])[C@@]31[C@@H](C)[C@H](O)[C@](C)(C=C)C[C@@H](OC(CN(C)CCN(C)C(CCN4C=NC5=C4N=CN=C5N6CCC(N)C6)=O)=O)[C@]2(C)[C@H](C)CC3	5.25
O=C(CC1)[C@]2([H])[C@@]31[C@@H](C)[C@H](O)[C@](C)(C=C)C[C@@H](OC(CN4CCN(C(CN5N=NC(CN6CCN(N(C)C)CC6)=C5)=O)CC4)=O)[C@]2(C)[C@H](C)CC3	5.24
O=C1CC[C@]2(CC[C@H]3C)[C@]1([H])[C@]3(C)[C@H](OC(CN4CCN(C(C(C(C)CC)NC(OC(C)(C)C)=O)=O)CC4)=O)C[C@@](C)(C=C)[C@@H](O)[C@@H]2C	5.22
O=C1CC[C@]2(CC[C@H]3C)[C@]1([H])[C@]3(C)[C@H](OC(CSC4=NC(NC=C5)=C5C(NC(CN6CCNCC6)=O)=N4)=O)C[C@@](C)(C=C)[C@@H](O)[C@@H]2C	5.21
O=C(CC1)[C@]2([H])[C@@]31[C@@H](C)[C@H](O)[C@](C)(C=C)C[C@@H](OC(CSC4=NN=C(C)N4/N=C/C5=CC=C(N(CC)CC)C=C5)=O)[C@]2(C)[C@H](C)CC3	5.21
O=C1CC[C@]2(CC[C@H]3C)[C@]1([H])[C@]3(C)[C@H](OC(CN4CCN(C(NC5=CN=C(Br)C=C5)=O)CC4)=O)C[C@@](C)(C=C)[C@@H](O)[C@@H]2C	5.21
O=C(CC1)[C@]2([H])[C@@]31[C@@H](C)[C@H](O)[C@](C)(C=C)C[C@@H](OC(CN4N=NC(CN5CCN(C6=CC=CC=C6Cl)CC5)=C4)=O)[C@]2(C)[C@H](C)CC3	5.20
O=C(CC1)[C@]2([H])[C@@]31[C@@H](C)[C@H](O)[C@](C)(C=C)C[C@@H](OC(CN4N=NC(CN5CCN(C6=CC=CC(Cl)=C6)CC5)=C4)=O)[C@]2(C)[C@H](C)CC3	5.20
O=C(CC1)[C@]2([H])[C@@]31[C@@H](C)[C@H](O)[C@](C)(C=C)C[C@@H](OC(CN4N=NC(CN5CCN(C6=CC=C(Cl)C=C6)CC5)=C4)=O)[C@]2(C)[C@H](C)CC3	5.20
O=C1CC[C@]2(CC[C@H]3C)[C@]1([H])[C@]3(C)[C@H](OC(CSC4=NC(O)=CC(NC(C5=CC=C(OC)C=C5)=O)=N4)=O)C[C@@](C)(C=C)[C@@H](O)[C@@H]2C	5.20
O=C(CC1)[C@]2([H])[C@@]31[C@@H](C)[C@H](O)[C@](C)(C=C)C[C@@H](OC(CSC4=NC(N)=CC(NC(C5=CC=C(OC)C=C5)=O)=N4)=O)[C@]2(C)[C@H](C)CC3	5.20
O=C(CC1)[C@]2([H])[C@@]31[C@@H](C)[C@H](O)[C@](C)(C=C)C[C@@H](OC(CN4N=NC(CN5CCN(C6=CC=CC=C6OC)CC5)=C4)=O)[C@]2(C)[C@H](C)CC3	5.20
O=C(CC1)[C@]2([H])[C@@]31[C@@H](C)[C@H](O)[C@](C)(C=C)C[C@@H](OC(CN4N=NC(CN5CCN(C6=CC=CC(OC)=C6)CC5)=C4)=O)[C@]2(C)[C@H](C)CC3	5.20
O=C(CC1)[C@]2([H])[C@@]31[C@@H](C)[C@H](O)[C@](C)(C=C)C[C@@H](OC(CN4N=NC(CN5CCN(C6=CC=C(OC)C=C6)CC5)=C4)=O)[C@]2(C)[C@H](C)CC3	5.20
O=C(CC1)[C@]2([H])[C@@]31[C@@H](C)[C@H](O)[C@](C)(C=C)C[C@@H](OC(CSCC4=CSC(NC(CN5CCNCC5)=O)=N4)=O)[C@]2(C)[C@H](C)CC3	5.20
O=C(CC1)[C@]2([H])[C@@]31[C@@H](C)[C@H](O)[C@](C)(C=C)C[C@@H](OC(CSC4=NN=C(NC(C5=CC=C(Cl)C=C5)=O)S4)=O)[C@]2(C)[C@H](C)CC3	5.20
O=C1CC[C@]2(CC[C@H]3C)[C@]1([H])[C@]3(C)[C@H](OC(CN4CCN(C(NCCC5=CC(OC)=CC=C5)=O)CC4)=O)C[C@@](C)(C=C)[C@@H](O)[C@@H]2C	5.19

O=C(CC1)[C@]2([H])[C@@]31[C@@H](C)[C@H](O)[C@](C)(C=C)C[C@@H](OC(CN4N=NC(CN(C5CCCCC5)C6CCCCC6)=C4)=O)[C@]2(C)[C@H](C)CC3	5.19
O=C1CC[C@]2(CC[C@H]3C)[C@]1([H])[C@]3(C)[C@H](OC(CN4CCN(C(NC5=CC=CC(NC(C)=O)=C5)=O)CC4)=O)C[C@](C)(C=C)[C@@H](O)[C@@H]2C	5.19
O=C(CC1)[C@]2([H])[C@@]31[C@@H](C)[C@H](O)[C@](C)(C=C)C[C@@H](OC(CN4N=NC(CN5CCN(C6=CC=CC(F)=C6)CC5)=C4)=O)[C@]2(C)[C@H](C)CC3	5.19
O=C(CC1)[C@]2([H])[C@@]31[C@@H](C)[C@H](O)[C@](C)(C=C)C[C@@H](OC(CN4N=NC(CN5CCN(C6=CC=C(F)C=C6)CC5)=C4)=O)[C@]2(C)[C@H](C)CC3	5.19
O=C1CC[C@]2(CC[C@H]3C)[C@]1([H])[C@]3(C)[C@H](OC(CSC4=NC(O)=CC(NC(C5=CC=C(C)C=C5)=O)=N4)=O)C[C@](C)(C=C)[C@@H](O)[C@@H]2C	5.19
O=C(CC1)[C@]2([H])[C@@]31[C@@H](C)[C@H](O)[C@](C)(C=C)C[C@@H](OC(CSC4=NC(N)=CC(NC(C5=CC=C(C)C=C5)=O)=N4)=O)[C@]2(C)[C@H](C)CC3	5.19
O=C(CC1)[C@]2([H])[C@@]31[C@@H](C)[C@H](O)[C@](C)(C=C)C[C@@H](OC(CN4N=NC(CN5CCN(C6=CC=CC=C6O)CC5)=C4)=O)[C@]2(C)[C@H](C)CC3	5.19
O=C(CC1)[C@]2([H])[C@@]31[C@@H](C)[C@H](O)[C@](C)(C=C)C[C@@H](OC(CN4N=NC(CN5CCN(C6=CC=CC(O)=C6)CC5)=C4)=O)[C@]2(C)[C@H](C)CC3	5.19
O=C(CC1)[C@]2([H])[C@@]31[C@@H](C)[C@H](O)[C@](C)(C=C)C[C@@H](OC(CN4N=NC(C5=CC=C(CN6CCN(C)CC6)C=C5)=C4)=O)[C@]2(C)[C@H](C)CC3	5.19
O=C(CC1)[C@]2([H])[C@@]31[C@@H](C)[C@H](O)[C@](C)(C=C)C[C@@H](OC(CN4N=NC(CN5CCN(C6=CC=C(C)C=C6)CC5)=C4)=O)[C@]2(C)[C@H](C)CC3	5.19
O=C1CC[C@]2(CC[C@H]3C)[C@]1([H])[C@]3(C)[C@H](OC(CSC4=NC(O)=CC(NC([C@@H]5NCCCC5)=O)=N4)=O)C[C@](C)(C=C)[C@@H](O)[C@@H]2C	5.19
O=C(CC1)[C@]2([H])[C@@]31[C@@H](C)[C@H](O)[C@](C)(C=C)C[C@@H](OC(CSC4=NC(N)=CC(NC([C@H]5CCCCN5)=O)=N4)=O)[C@]2(C)[C@H](C)CC3	5.19
O=C(CC1)[C@]2([H])[C@@]31[C@@H](C)[C@H](O)[C@](C)(C=C)C[C@@H](OC(CSC4=NN=C(NC(C5=CC=CC=C5N)=O)S4)=O)[C@]2(C)[C@H](C)CC3	5.19
O=C(CC1)[C@]2([H])[C@@]31[C@@H](C)[C@H](O)[C@](C)(C=C)C[C@@H](OC(CN4N=NC(CN5CCN(C6=CC=CC=C6)CC5)=C4)=O)[C@]2(C)[C@H](C)CC3	5.18
O=C1CC[C@]2(CC[C@H]3C)[C@]1([H])[C@]3(C)[C@H](OC(CN4CCN(C(CNC(OC(C)(C)C)=O)=O)CC4)=O)C[C@](C)(C=C)[C@@H](O)[C@@H]2C	5.18
O=C1CC[C@]2(CC[C@H]3C)[C@]1([H])[C@]3(C)[C@H](OC(CN4CCN(C(NCCN5CCOCC5)=O)CC4)=O)C[C@](C)(C=C)[C@@H](O)[C@@H]2C	5.18
O=C(CC1)[C@]2([H])[C@@]31[C@@H](C)[C@H](O)[C@](C)(C=C)C[C@@H](OC(CN4CCN(C(NCCN5CCCCC5)=O)CC4)=O)[C@]2(C)[C@H](C)CC3	5.18
O=C1CC[C@]2(CC[C@H]3C)[C@]1([H])[C@]3(C)[C@H](OC(CN4CCN(C(NCCN5CCCCC5)=O)CC4)=O)C[C@](C)(C=C)[C@@H](O)[C@@H]2C	5.18
O=C(CC1)[C@]2([H])[C@@]31[C@@H](C)[C@H](O)[C@](C)(C=C)C[C@@H](OC(CSC(C)(C)CNC(OCC4=CC=CC=C4)=O)=O)[C@]2(C)[C@H](C)CC3	5.18
O=C(CC1)[C@]2([H])[C@@]31[C@@H](C)[C@H](O)[C@](C)(C=C)C[C@@H](OC(CS(S(C4=NN=NN4CCN(C)C)=O)=O)=O)[C@]2(C)[C@H](C)CC3	5.17
O=C1CC[C@]2(CC[C@H]3C)[C@]1([H])[C@]3(C)[C@H](OC(CN4CCN(C(NCCC5=CC=CC=C5)=O)CC4)=O)C[C@](C)(C=C)[C@@H](O)[C@@H]2C	5.17
N[C@@H](CC1=CC=CC=C1)C(N(CC2)CCN2CC(O[C@@H]([C@]3(C)[C@H](C)CC4)C[C@@](C)(C=C)[C@@H](O)[C@H](C)[C@@]54[C@@]3([H])C(CC5)=O)=O)=O	5.17
O=C(CC1)[C@]2([H])[C@@]31[C@@H](C)[C@H](O)[C@](C)(C=C)C[C@@H](OC(CSC(C)(C)CNC(CN4CCOCC4)=O)=O)[C@]2(C)[C@H](C)CC3	5.17
O=C(CC1)[C@]2([H])[C@@]31[C@@H](C)[C@H](O)[C@](C)(C=C)C[C@@H](OC(CN4N=NC(C5=CC=C(CN(CC)CC)C=C5)=C4)=O)[C@]2(C)[C@H](C)CC3	5.17
O=C(CC1)[C@]2([H])[C@@]31[C@@H](C)[C@H](O)[C@](C)(C=C)C[C@@H](OC(CSC4=NN=C(NC(N5CCCC5)=O)S4)=O)[C@]2(C)[C@H](C)CC3	5.17
O=C(CC1)[C@]2([H])[C@@]31[C@@H](C)[C@H](O)[C@](C)(C=C)C[C@@H](OC(CN4CCN(C(CNC5CCCCC5)=O)CC4)=O)[C@]2(C)[C@H](C)CC3	5.17
N[C@@H](C1CCCCC1)C(N(CC2)CCN2CC(O[C@@H]([C@]3(C)[C@H](C)CC4)C[C@@](C)(C=C)[C@@H](O)[C@H](C)[C@@]54[C@@]3([H])C(CC5)=O)=O)=O	5.17
O=C1CC[C@]2(CC[C@H]3C)[C@]1([H])[C@]3(C)[C@H](OC(CN4CCN(C(NC5=NC(N)=CC=C5)=O)CC4)=O)C[C@](C)(C=C)[C@@H](O)[C@@H]2C	5.16
O=C1CC[C@]2(CC[C@H]3C)[C@]1([H])[C@]3(C)[C@H](OC(CN4CCN(C(NCC5=CC=CC=C5)=O)CC4)=O)C[C@](C)(C=C)[C@@H](O)[C@@H]2C	5.16
O=C(CC1)[C@]2([H])[C@@]31[C@@H](C)[C@H](O)[C@](C)(C=C)C[C@@H](OC(CSC(C)(C)CNC(CN4CCCC4)=O)=O)[C@]2(C)[C@H](C)CC3	5.16
O=C(CC1)[C@]2([H])[C@@]31[C@@H](C)[C@H](O)[C@](C)(C=C)C[C@@H](OC(CN4CCN(C(NC5=CC=NC=C5)=O)CC4)=O)[C@]2(C)[C@H](C)CC3	5.15
N[C@@H](CC(C)C)C(N(CC1)CCN1CC(O[C@@H]([C@]2(C)[C@H](C)CC3)C[C@@](C)(C=C)[C@@H](O)[C@H](C)[C@@]43[C@@]2([H])C(CC4)=O)=O)=O	5.15
O=C1CC[C@]2(CC[C@H]3C)[C@]1([H])[C@]3(C)[C@H](OC(CN4CCN(C(C(C(C)CC)N)=O)CC4)=O)C[C@](C)(C=C)[C@@H](O)[C@@H]2C	5.15
O=C(CC1)[C@]2([H])[C@@]31[C@@H](C)[C@H](O)[C@](C)(C=C)C[C@@H](OC(CN4CCN(C(CN5CCCC5)=O)CC4)=O)[C@]2(C)[C@H](C)CC3	5.14
O=C1CC[C@]2(CC[C@H]3C)[C@]1([H])[C@]3(C)[C@H](OC(CN4CCN(C([C@H]([C@@H](C)O)N)=O)CC4)=O)C[C@](C)(C=C)[C@@H](O)[C@@H]2C	5.14
N[C@@H](C(C)C)C(N(CC1)CCN1CC(O[C@@H]([C@]2(C)[C@H](C)CC3)C[C@@](C)(C=C)[C@@H](O)[C@H](C)[C@@]43[C@@]2([H])C(CC4)=O)=O)=O	5.13
O=C1CC[C@]2(CC[C@H]3C)[C@]1([H])[C@]3(C)[C@H](OC(CN4CCN(C5=CC=C(O)C=C5)CC4)=O)C[C@](C)(C=C)[C@@H](O)[C@@H]2C	5.13
O=C1CC[C@]2(CC[C@H]3C)[C@]1([H])[C@]3(C)[C@H](OC(CN4CCN(C5=CC=C(C)C=C5)CC4)=O)C[C@](C)(C=C)[C@@H](O)[C@@H]2C	5.13
O=C(CC1)[C@]2([H])[C@@]31[C@@H](C)[C@H](O)[C@](C)(C=C)C[C@@H](OC(CN4N=NC(C5=CC=CC=C5CN)=C4)=O)[C@]2(C)[C@H](C)CC3	5.13
O=C(CC1)[C@]2([H])[C@@]31[C@@H](C)[C@H](O)[C@](C)(C=C)C[C@@H](OC(CNCC4=CC=C(F)C=C4)=O)[C@]2(C)[C@H](C)CC3	5.08
O=C(CC1)[C@]2([H])[C@@]31[C@@H](C)[C@H](O)[C@](C)(C=C)C[C@@H](OC(CN4CCCCC4)=O)[C@]2(C)[C@H](C)CC3	5.05
O=C(CC1)[C@]2([H])[C@@]31[C@@H](C)[C@H](O)[C@](C)(C=C)C[C@@H](OC(CNCCCC)=O)[C@]2(C)[C@H](C)CC3	5.04
O=C(CC1)[C@]2([H])[C@@]31[C@@H](C)[C@H](O)[C@](C)(C=C)C[C@@H](OC(CNC(C)CC)=O)[C@]2(C)[C@H](C)CC3	5.04
O=C(CC1)[C@]2([H])[C@@]31[C@@H](C)[C@H](O)[C@](C)(C(CO)O)C[C@@H](OC(CO)=O)[C@]2(C)[C@H](C)CC3	5.01
O=C(CC1)[C@]2([H])[C@@]31[C@@H](C)[C@H](O)[C@](C)(C4CO4)C[C@@H](OC(CO)=O)[C@]2(C)[C@H](C)CC3	4.99
O=C1CC[C@]2(CC[C@H]3C)[C@]1([H])[C@]3(C)[C@H](OC(CN4CCN(C([C@H](CCCCNC(OC(C)(C)C)=O)NC(OC(C)(C)C)=O)=O)CC4)=O)C[C@](C)(C=C)[C@@H](O)[C@@H]2C	4.99
O=C(CC1)[C@]2([H])[C@@]31[C@@H](C)[C@H](O)[C@](C)(C=C)C[C@@H](OC(CSC4=CC(C(NC5=CC=C(S(=O)(NC6=NC(C)=CC(C)=N6)=O)C=C5)=O)=CC=C4)=O)[C@]2(C)[C@H](C)CC3	4.99
O=C(CC1)[C@]2([H])[C@@]31[C@@H](C)[C@H](O)[C@](C)(C=C)C[C@@H](OC(CN4CCN(C(CN5N=NC(CN6CCN(C7=CC=CC=C7Cl)CC6)=C5)=O)CC4)=O)[C@]2(C)[C@H](C)CC3	4.98
O=C(CC1)[C@]2([H])[C@@]31[C@@H](C)[C@H](O)[C@](C)(C=C)C[C@@H](OC(CSC4=CC(C(NC5=CC=C(S(=O)(NC6=NOC(C)=C6)=O)C=C5)=O)=CC=C4)=O)[C@]2(C)[C@H](C)CC3	4.97
O=C(CC1)[C@]2([H])[C@@]31[C@@H](C)[C@H](O)[C@](C)(C=C)C[C@@H](OC(CN4CCN(C(CN5N=NC(CN6CCN(C7=CC=CC=C7F)CC6)=C5)=O)CC4)=O)[C@]2(C)[C@H](C)CC3	4.97
O=C(CC1)[C@]2([H])[C@@]31[C@@H](C)[C@H](O)[C@](C)(C=C)C[C@@H](OC(CN4CCN(C(CN5N=NC(CN6CCN(C7=CC=CC=C7O)CC6)=C5)=O)CC4)=O)[C@]2(C)[C@H](C)CC3	4.97
O=C(CC1)[C@]2([H])[C@@]31[C@@H](C)[C@H](O)[C@](C)(C=C)C[C@@H](OC(CN4CCN(C(CN5N=NC(CN6CCN(C7=CC=C(C)C=C7)CC6)=C5)=O)CC4)=O)[C@]2(C)[C@H](C)CC3	4.97
O=C(CC1)[C@]2([H])[C@@]31[C@@H](C)[C@H](O)[C@](C)(C=C)C[C@@H](OC(CN4CCN(C(CN5N=NC(CN6CCN(N7CCOCC7)CC6)=C5)=O)CC4)=O)[C@]2(C)[C@H](C)CC3	4.97
O=C(CC1)[C@]2([H])[C@@]31[C@@H](C)[C@H](O)[C@](C)(C=C)C[C@@H](OC(CSCC4=CSC(NC(CN5CCN(CC6=CC=CC=C6)CC5)=O)=N4)=O)[C@]2(C)[C@H](C)CC3	4.96
O=C(CC1)[C@]2([H])[C@@]31[C@@H](C)[C@H](O)[C@](C)(C=C)C[C@@H](OC(CSC4=C(NC(CN5CCN(C6=C(Cl)C=CC=C6)CC5)=O)C=CC=C4)=O)[C@]2(C)[C@H](C)CC3	4.96
O=C(CC1)[C@]2([H])[C@@]31[C@@H](C)[C@H](O)[C@](C)(C=C)C[C@@H](OC(CSC4=C(NC(CN5CCN(C6=CC(C)=CC=C6)CC5)=O)C=CC=C4)=O)[C@]2(C)[C@H](C)CC3	4.94
O=C(N(CC1)CCN1CC(O[C@@H]([C@]2(C)[C@H](C)CC3)C[C@@](C)(C=C)[C@@H](O)[C@H](C)[C@@]43[C@@]2([H])C(CC4)=O)=O)[C@H](C5CCCCC5)NC(OC(C)(C)C)=O	4.93
O=C(CC1)[C@]2([H])[C@@]31[C@@H](C)[C@H](O)[C@](C)(C=C)C[C@@H](OC(CN4N=NC(C5=CC=CC(CC6=CC=C(CN(CC)CC)C=C6)=C5)=C4)=O)[C@]2(C)[C@H](C)CC3	4.93

O=C(CC1)[C@]2([H])[C@@]31[C@@H](C)[C@H](O)[C@](C)(C=C)C[C@@H](OC(CSCC4=CSC(NC(CN(CCCC)CCCC)=O)=N4)=O)[C@]2(C)[C@H](C)CC3	4.93
O=C(CC1)[C@]2([H])[C@@]31[C@@H](C)[C@H](O)[C@](C)(C=C)C[C@@H](OC(N4CC5=C(C=CC=C5OCCCCN6C7=CC=CN=C7N=C6)C4=O)=O)[C@]2(C)[C@H](C)CC3	4.92
O=C(CC1)[C@]2([H])[C@@]31[C@@H](C)[C@H](O)[C@](C)(C=C)C[C@@H](OC(CN4CCN(C(CN5N=NC(CN6CCN(C)CC6)=C5)=O)CC4)=O)[C@]2(C)[C@H](C)CC3	4.92
O=C(CC1)[C@]2([H])[C@@]31[C@@H](C)[C@H](O)[C@](C)(C=C)C[C@@H](OC(CN4CCN(C5=CC=C(C(/C=C/C6=CC=CC(N)=C6)=O)C=C5)CC4)=O)[C@]2(C)[C@H](C)CC3	4.92
O=C(N(CC1)CCN1CC(O[C@@H]([C@]2(C)[C@H](C)CC3)C[C@@](C)(C=C)[C@@H](O)[C@H](C)[C@@]43[C@@]2([H])C(CC4)=O)=O)[C@H](CC(C)C)NC(OC(C)(C)C)=O	4.92
O=C(CC1)[C@]2([H])[C@@]31[C@@H](C)[C@H](O)[C@](C)(C=C)C[C@@H](OC(CSC4=NN=C(NC(C5=CC(OC)=CC=C5)=O)S4)=O)[C@]2(C)[C@H](C)CC3	4.89
O=C(CC1)[C@]2([H])[C@@]31[C@@H](C)[C@H](O)[C@](C)(C=C)C[C@@H](OC(CSC4=NN=C(NC([C@H](C5=CC=CC=C5)C)=O)S4)=O)[C@]2(C)[C@H](C)CC3	4.89
O=C(CC1)[C@]2([H])[C@@]31[C@@H](C)[C@H](O)[C@](C)(C=C)C[C@@H](OC(CN4N=NC(C5=CC=C(CC6=CC=C(OC)C=C6)C=C5)=C4)=O)[C@]2(C)[C@H](C)CC3	4.89
O=C(CC1)[C@]2([H])[C@@]31[C@@H](C)[C@H](O)[C@](C)(C=C)C[C@@H](OC(CN4N=NC(COCCN5C=NC6=C5N=CN=C6N)=C4)=O)[C@]2(C)[C@H](C)CC3	4.89
O=C(CC1)[C@]2([H])[C@@]31[C@@H](C)[C@H](O)[C@](C)(C=C)C[C@@H](OC(CSC4=NN=C(NC(N5CCN(C)CC5)=O)S4)=O)[C@]2(C)[C@H](C)CC3	4.89
O=C(CC1)[C@]2([H])[C@@]31[C@@H](C)[C@H](O)[C@](C)(C=C)C[C@@H](OC(CN4N=NC(CN5CCN(C6=CC=C(O)C=C6)CC5)=C4)=O)[C@]2(C)[C@H](C)CC3	4.89
O=C(CC1)[C@]2([H])[C@@]31[C@@H](C)[C@H](O)[C@](C)(C=C)C[C@@H](OC(CSCC4=CSC(NC(C5=CC=CS5)=O)=N4)=O)[C@]2(C)[C@H](C)CC3	4.89
O=C1CC[C@]2(CC[C@H]3C)[C@]1([H])[C@]3(C)[C@H](OC(CSC4=NC(O)=CC(NC([C@H]5NCCCC5)=O)=N4)=O)C[C@@](C)(C=C)[C@@H](O)[C@@H]2C	4.89
O=C(CC1)[C@]2([H])[C@@]31[C@@H](C)[C@H](O)[C@](C)(C=C)C[C@@H](OC(CSC4=NC(N)=CC(NC([C@@H]5CCCCN5)=O)=N4)=O)[C@]2(C)[C@H](C)CC3	4.88
O=C(CC1)[C@]2([H])[C@@]31[C@@H](C)[C@H](O)[C@](C)(C=C)C[C@@H](OC(CN4N=NC=C4CCCN5C(N=CN=C6N)=C6N=C5)=O)[C@]2(C)[C@H](C)CC3	4.88
O=C(CC1)[C@]2([H])[C@@]31[C@@H](C)[C@H](O)[C@](C)(C=C)C[C@@H](OC(CSC(C)(C)CNC(C4=CC(Cl)=CC=C4)=O)=O)[C@]2(C)[C@H](C)CC3	4.88
O=C1CC[C@]2(CC[C@H]3C)[C@]1([H])[C@]3(C)[C@H](OC(CN4CCN(C(NCC5=CC=C(N)C=C5)=O)CC4)=O)C[C@@](C)(C=C)[C@@H](O)[C@@H]2C	4.87
O=C(CC1)[C@]2([H])[C@@]31[C@@H](C)[C@H](O)[C@](C)(C=C)C[C@@H](OC(CSC4=NN=C(NC(N(CC)CC)=O)S4)=O)[C@]2(C)[C@H](C)CC3	4.87
O=C(CC1)[C@]2([H])[C@@]31[C@@H](C)[C@H](O)[C@](C)(C=C)C[C@@H](OC(CSC4=NN=C(NC([C@@H](C(C)C)N)=O)S4)=O)[C@]2(C)[C@H](C)CC3	4.87
O=C(CC1)[C@]2([H])[C@@]31[C@@H](C)[C@H](O)[C@](C)(C=C)C[C@@H](OC(CN4CCN(C(CN(CCC)CCC)=O)CC4)=O)[C@]2(C)[C@H](C)CC3	4.87
O=C1CC[C@]2(CC[C@H]3C)[C@]1([H])[C@]3(C)[C@H](OC(CN4CCN(C(NC5=CC(N)=CC=C5)=O)CC4)=O)C[C@@](C)(C=C)[C@@H](O)[C@@H]2C	4.86
O=C1CC[C@]2(CC[C@H]3C)[C@]1([H])[C@]3(C)[C@H](OC(CN4CCN(C(NC5=CC=C(N)C=C5)=O)CC4)=O)C[C@@](C)(C=C)[C@@H](O)[C@@H]2C	4.86
O=C(CC1)[C@]2([H])[C@@]31[C@@H](C)[C@H](O)[C@](C)(C=C)C[C@@H](OC(CN4N=NC(CN5CCCC(CCO)C5)=C4)=O)[C@]2(C)[C@H](C)CC3	4.85
O=C(CC1)[C@]2([H])[C@@]31[C@@H](C)[C@H](O)[C@](C)(C=C)C[C@@H](OC(CN4CCN(C(CN(CC)CC)=O)CC4)=O)[C@]2(C)[C@H](C)CC3	4.84
O=C(CC1)[C@]2([H])[C@@]31[C@@H](C)[C@H](O)[C@](C)(C=C)C[C@@H](OC(CN4N=NC(CN5CCCC(CO)C5)=C4)=O)[C@]2(C)[C@H](C)CC3	4.84
O=C(CC1)[C@]2([H])[C@@]31[C@@H](C)[C@H](O)[C@](C)(C=C)C[C@@H](OC(CN4N=NC(CN5CCC(O)CC5)=C4)=O)[C@]2(C)[C@H](C)CC3	4.83
O=C(CC1)[C@]2([H])[C@@]31[C@@H](C)[C@H](O)[C@](C)(C=C)C[C@@H](OC(CN4N=NC(CN5CCN(C)CC5)=C4)=O)[C@]2(C)[C@H](C)CC3	4.83
O=C(CC1)[C@]2([H])[C@@]31[C@@H](C)[C@H](O)[C@](C)(C=C)C[C@@H](OC(CSC4=NC(C=CC(NC(C5=CC6=C(C=CC=C6)N5)=O)=C7)=C7N4)=O)[C@]2(C)[C@H](C)CC3	4.83
O=C(CC1)[C@]2([H])[C@@]31[C@@H](C)[C@H](O)[C@](C)(C=C)C[C@@H](OC(CN4N=NC(CN5CCOCC5)=C4)=O)[C@]2(C)[C@H](C)CC3	4.82
O=C(CC1)[C@]2([H])[C@@]31[C@@H](C)[C@H](O)[C@](C)(C=C)C[C@@H](OC(CN4N=NC(CN(CC)CC)=C4)=O)[C@]2(C)[C@H](C)CC3	4.81
O=C(CC1)[C@]2([H])[C@@]31[C@@H](C)[C@H](O)[C@](C)(C=C)C[C@@H](OC(CN4N=NC(CN(C)C)=C4)=O)[C@]2(C)[C@H](C)CC3	4.78
O=C(CC1)[C@]2([H])[C@@]31[C@@H](C)[C@H](O)[C@](C)(C=C)C[C@@H](OC(CN(CC)CC)=O)[C@]2(C)[C@H](C)CC3	4.73
O=C(CC1)[C@]2([H])[C@@]31[C@@H](C)[C@H](O)[C@](C)(C=C)C[C@@H](OC(C[N+](CC)(CC)C)=O)[C@]2(C)[C@H](C)CC3	4.70
O=C(CC1)[C@]2([H])[C@@]31[C@@H](C)[C@H](O)[C@](C)(C=C)C[C@@H](OC(CN4CCN(C(CN5N=NC(CN6CCN(N7CCC(CCO)CC7)CC6)=C5)=O)CC4)=O)[C@]2(C)[C@H](C)CC3	4.69
O=C(CC1)[C@]2([H])[C@@]31[C@@H](C)[C@H](O)[C@](C)(C=C)C[C@@H](OC(CN4CCN(C(CN5N=NC(CN6CCN(N7CCCC(CO)C7)CC6)=C5)=O)CC4)=O)[C@]2(C)[C@H](C)CC3	4.69
O=C(CC1)[C@]2([H])[C@@]31[C@@H](C)[C@H](O)[C@](C)(C=C)C[C@@H](OC(CSC4=CC(C(NC5=CC=C(S(=O)(NC6=CC(OC)=NC=N6)=O)C=C5)=O)=CC=C4)=O)[C@]2(C)[C@H](C)CC3	4.69
CC1C23C(C(CC3)=O)C(C(CC(C=C)(C)C1O)OC(CO)=O)(C(CC2)C)C	4.67
O=C(CC1)[C@]2([H])[C@@]31[C@@H](C)[C@H](O)[C@](C)(C=C)C[C@@H](OC(CN4CCN(C(CN5N=NC(CN6CCN(N7CCC(O)CC7)CC6)=C5)=O)CC4)=O)[C@]2(C)[C@H](C)CC3	4.67
O=C(CC1)[C@]2([H])[C@@]31[C@@H](C)[C@H](O)[C@](C)(C=C)C[C@@H](OC(CSC4=C(NC(CN5CCN(C6=C(OC)C=CC=C6)CC5)=O)C=CC=C4)=O)[C@]2(C)[C@H](C)CC3	4.65
O=C(CC1)[C@]2([H])[C@@]31[C@@H](C)[C@H](O)[C@](C)(C=C)C[C@@H](OC(CN4CCN(C5=CC=C(C(/C=C/C6=CC=C(NC(C)=O)C=C6)=O)C=C5)CC4)=O)[C@]2(C)[C@H](C)CC3	4.65
O=C(CC1)[C@]2([H])[C@@]31[C@@H](C)[C@H](O)[C@](C)(C=C)C[C@@H](OC(CSC4=C(NC(CN5CCN(C6=CC=C(C)C=C6)CC5)=O)C=CC=C4)=O)[C@]2(C)[C@H](C)CC3	4.64
O=C1CC[C@]2(CC[C@H]3C)[C@]1([H])[C@]3(C)[C@H](OC(CN4CCN(C(C(CC5=CNC6=C5C=CC=C6)N)=O)CC4)=O)C[C@@](C)(C=C)[C@@H](O)[C@@H]2C	4.60
O=C(CC1)[C@]2([H])[C@@]31[C@@H](C)[C@H](O)[C@](C)(C=C)C[C@@H](OC(CN4N=NC(C5=CC=CC(CC6=CC=C(OC)C=C6)=C5)=C4)=O)[C@]2(C)[C@H](C)CC3	4.59
O=C(CC1)[C@]2([H])[C@@]31[C@@H](C)[C@H](O)[C@](C)(C=C)C[C@@H](OC(CSC4=NN=C(C5=CC=C(Br)C=C5)O4)=O)[C@]2(C)[C@H](C)CC3	4.59
O=C(CC1)[C@]2([H])[C@@]31[C@@H](C)[C@H](O)[C@](C)(C=C)C[C@@H](OC(CSCC4=CSC(NC(CN5C=CC=C5)=O)=N4)=O)[C@]2(C)[C@H](C)CC3	4.58
O=C(CC1)[C@]2([H])[C@@]31[C@@H](C)[C@H](O)[C@](C)(C=C)C[C@@H](OC(CN4CCN(C(CNC5=CC=CC=C5)=O)CC4)=O)[C@]2(C)[C@H](C)CC3	4.56
O=C1CC[C@]2(CC[C@H]3C)[C@]1([H])[C@]3(C)[C@H](OC(CN4CCN(C(N(C5=CC=CC=C5)C)=O)CC4)=O)C[C@@](C)(C=C)[C@@H](O)[C@@H]2C	4.56
O=C1CC[C@]2(CC[C@H]3C)[C@]1([H])[C@]3(C)[C@H](OC(CN4CCN(C([C@H](CCCCN)N)=O)CC4)=O)C[C@@](C)(C=C)[C@@H](O)[C@@H]2C	4.56
O=C(CC1)[C@]2([H])[C@@]31[C@@H](C)[C@H](O)[C@](C)(C=C)C[C@@H](OC(CN4CCN(C(CN5CCOCC5)=O)CC4)=O)[C@]2(C)[C@H](C)CC3	4.55
O=C(CC1)[C@]2([H])[C@@]31[C@@H](C)[C@H](O)[C@](C)(C=C)C[C@@H](OC(CSC4=NN=C(C5=CC=CC=C5Cl)O4)=O)[C@]2(C)[C@H](C)CC3	4.55
O=C(CC1)[C@]2([H])[C@@]31[C@@H](C)[C@H](O)[C@](C)(C=C)C[C@@H](OC(CSC4=NN=C(C5=CC=C(Cl)C=C5)O4)=O)[C@]2(C)[C@H](C)CC3	4.55
O=C(CC1)[C@]2([H])[C@@]31[C@@H](C)[C@H](O)[C@](C)(C=C)C[C@@H](OC(CN4CCN(C(CN5CCCCC5)=O)CC4)=O)[C@]2(C)[C@H](C)CC3	4.55
O=C1CC[C@]2(CC[C@H]3C)[C@]1([H])[C@]3(C)[C@H](OC(CN4CCN(C(NC5=CC=NC=C5)=O)CC4)=O)C[C@@](C)(C=C)[C@@H](O)[C@@H]2C	4.55
O=C(CC1)[C@]2([H])[C@@]31[C@@H](C)[C@H](O)[C@](C)(C=C)C[C@@H](OC(CS(SC4=NC(O)=CC(O)=N4)=O)=O)[C@]2(C)[C@H](C)CC3	4.54
O=C(CC1)[C@]2([H])[C@@]31[C@@H](C)[C@H](O)[C@](C)(C=C)C[C@@H](OC(CN4CCN(C(CN(C)C)=O)CC4)=O)[C@]2(C)[C@H](C)CC3	4.52
O=C(CC1)[C@]2([H])[C@@]31[C@@H](C)[C@H](O)[C@](C)(C=C)C[C@@H](OC(CSC4=NC(C=CC(NC(C5=CC=CC(Cl)=C5)=O)=C6)=C6N4)=O)[C@]2(C)[C@H](C)CC3	4.52

O=C(CC1)[C@]2([H])[C@@]31[C@@H](C)[C@H](O)[C@](C)(C=C)C[C@@H](OC(CSC4=NC(C=CC(NC(C5=CC=C(OC)C=C5)=O)=C6)=C6N4)=O)[C@]2(C)[C@H](C)CC3	4.52
O=C(CC1)[C@]2([H])[C@@]31[C@@H](C)[C@H](O)[C@](C)(C=C)C[C@@H](OC(CNCCCCCCCC)=O)[C@]2(C)[C@H](C)CC3	4.49
O=C(CC1)[C@]2([H])[C@@]31[C@@H](C)[C@H](O)[C@](C)(C=C)C[C@@H](OC(C[N+]4(CCOCC4)C)=O)[C@]2(C)[C@H](C)CC3	4.46
O=C(CC1)[C@]2([H])[C@@]31[C@@H](C)[C@H](O)[C@](C)(C=C)C[C@@H](OC(CSC4=NN=CO4)=O)[C@]2(C)[C@H](C)CC3	4.46
O=C(CC1)[C@]2([H])[C@@]31[C@@H](C)[C@H](O)[C@](C)(C=C)C[C@@H](OC(C[N+]4(CCCCC4)C)=O)[C@]2(C)[C@H](C)CC3	4.46
O=C(CC1)[C@]2([H])[C@@]31[C@@H](C)[C@H](O)[C@](C)(C=C)C[C@@H](OC(CSCCNC(C4=CC=CC(OC)=C4)=O)=O)[C@]2(C)[C@H](C)CC3	4.36
O=C(CC1)[C@]2([H])[C@@]31[C@@H](C)[C@H](O)[C@](C)(C=C)C[C@@H](OC(N4CC5=C(C=CC=C5OCCCN6C7=CC=CN=C7N=C6)C4=O)=O)[C@]2(C)[C@H](C)CC3	4.31
O=C1CC[C@]2(CC[C@H]3C)[C@]1([H])[C@]3(C)[C@H](OC(CSC4=NC(O)=CC(NC(C5=CC=CC(Cl)=C5)=O)=N4)=O)C[C@@](C)(C=C)[C@@H](O)[C@@H]2C	4.30
O=C(CC1)[C@]2([H])[C@@]31[C@@H](C)[C@H](O)[C@](C)(C=C)C[C@@H](OC(CSC4=NC(N)=CC(NC(C5=CC(Cl)=CC=C5)=O)=N4)=O)[C@]2(C)[C@H](C)CC3	4.30
O=C(CC1)[C@]2([H])[C@@]31[C@@H](C)[C@H](O)[C@](C)(C=C)C[C@@H](OC(CSCC4=CSC(NC(CN5C=CN=N5)=O)=N4)=O)[C@]2(C)[C@H](C)CC3	4.28
O=C(CC1)[C@]2([H])[C@@]31[C@@H](C)[C@H](O)[C@](C)(C=C)C[C@@H](OC(CSCC4=CSC(NC(CN5C=CN=C5)=O)=N4)=O)[C@]2(C)[C@H](C)CC3	4.28
O=C(CC1)[C@]2([H])[C@@]31[C@@H](C)[C@H](O)[C@](C)(C=C)C[C@@H](OC(CNCCCCCCCCCCCCCCCC)=O)[C@]2(C)[C@H](C)CC3	4.27
O=C(CC1)[C@]2([H])[C@@]31[C@@H](C)[C@H](O)[C@](C)(C=C)C[C@@H](OC(CSC4=NN=C(NC([C@H](C(C)C)N)=O)S4)=O)[C@]2(C)[C@H](C)CC3	4.27
O=C(CC1)[C@]2([H])[C@@]31[C@@H](C)[C@H](O)[C@](C)(C=C)C[C@@H](OC(CN4CCN(C(CN5CCN(C)CC5)=O)CC4)=O)[C@]2(C)[C@H](C)CC3	4.26
O=C(CC1)[C@]2([H])[C@@]31[C@@H](C)[C@H](O)[C@](C)(C=C)C[C@@H](OC(CNCCCCCCCCCCCC)=O)[C@]2(C)[C@H](C)CC3	4.23
O=C1CC[C@]2(CC[C@H]3C)[C@]1([H])[C@]3(C)[C@H](OC(CN4CCN(C(C5N(C(OC(C)(C)C)=O)CCC5)=O)CC4)=O)C[C@@](C)(C=C)[C@@H](O)[C@@H]2C	4.23
O=C(CC1)[C@]2([H])[C@@]31[C@@H](C)[C@H](O)[C@](C)(C=C)C[C@@H](OC(CS[C@H]4CC[C@H](C(O)=O)C[C@H]4N)=O)[C@]2(C)[C@H](C)CC3	4.22
O=C(CC1)[C@]2([H])[C@@]31[C@@H](C)[C@H](O)[C@](C)(C=C)C[C@@H](OC(CSCC4=CC(C(O)=CN4O)=O)=O)[C@]2(C)[C@H](C)CC3	4.22
O=C(CC1)[C@]2([H])[C@@]31[C@@H](C)[C@H](O)[C@](C)(C=C)C[C@@H](OC(CSC4=NC(C=CC(NC(C5=CC=CC=C5Cl)=O)=C6)=C6N4)=O)[C@]2(C)[C@H](C)CC3	4.22
O=C(CC1)[C@]2([H])[C@@]31[C@@H](C)[C@H](O)[C@](C)(C=C)C[C@@H](OC(CSC4=NC(C=CC(NC(C5=CC=C(Cl)C=C5)=O)=C6)=C6N4)=O)[C@]2(C)[C@H](C)CC3	4.22
O=C(CC1)[C@]2([H])[C@@]31[C@@H](C)[C@H](O)[C@](C)(C=C)C[C@@H](OC(CSC4=NC(C=CC(NC(C5=CC=CC=C5OC)=O)=C6)=C6N4)=O)[C@]2(C)[C@H](C)CC3	4.22
O=C(CC1)[C@]2([H])[C@@]31[C@@H](C)[C@H](O)[C@](C)(C=C)C[C@@H](OC(CSC4=NC(C=CC(NC(C5=CC=CC(OC)=C5)=O)=C6)=C6N4)=O)[C@]2(C)[C@H](C)CC3	4.22
O=C(CC1)[C@]2([H])[C@@]31[C@@H](C)[C@H](O)[C@](C)(C=C)C[C@@H](OC(CSC4=NC(C=CC(NC(C5=CC=CC=C5C)=O)=C6)=C6N4)=O)[C@]2(C)[C@H](C)CC3	4.21
O=C(CC1)[C@]2([H])[C@@]31[C@@H](C)[C@H](O)[C@](C)(C=C)C[C@@H](OC(CSC4=NC(C=CC(NC(C5=CC=CC(C)=C5)=O)=C6)=C6N4)=O)[C@]2(C)[C@H](C)CC3	4.21
O=C(CC1)[C@]2([H])[C@@]31[C@@H](C)[C@H](O)[C@](C)(C=C)C[C@@H](OC(CSC4=NC(C=CC(NC(C5=CC=C(C)C=C5)=O)=C6)=C6N4)=O)[C@]2(C)[C@H](C)CC3	4.21
O=C(CC1)[C@]2([H])[C@@]31[C@@H](C)[C@H](O)[C@](C)(C=C)C[C@@H](OC(CN4CCN(C(CO)=O)CC4)=O)[C@]2(C)[C@H](C)CC3	4.20
O=C(CC1)[C@]2([H])[C@@]31[C@@H](C)[C@H](O)[C@](C)(C=C)C[C@@H](OC(CNC(C)(C)C)=O)[C@]2(C)[C@H](C)CC3	4.13
O=C(CC1)[C@]2([H])[C@@]31[C@@H](C)[C@H](O)[C@](C)(C=C)C[C@@H](OC(CS[C@H]4CC[C@H](C(OCC)=O)C[C@H]4NS(=O)(C5=CC=C(C(F)(F)F)C=C5)=O)=O)[C@]2(C)[C@H](C)CC3	4.08
O=C(CC1)[C@]2([H])[C@@]31[C@@H](C)[C@H](O)[C@](C)(C=C)C[C@@H](OC(CN4CCN(CC(NC5=CC=C(NCC6=CC=CC=C6)C=C5C)=O)CC4)=O)[C@]2(C)[C@H](C)CC3	4.04
O=C(CC1)[C@]2([H])[C@@]31[C@@H](C)[C@H](O)[C@](C)(C=C)C[C@@H](OC(CN4CCN(CC(NC5=CC=C(NCCC)C=C5C)=O)CC4)=O)[C@]2(C)[C@H](C)CC3	4.01
O=C(CC1)[C@]2([H])[C@@]31[C@@H](C)[C@H](O)[C@](C)(C=C)C[C@@H](OC(CN4CCN(CC(NC5=CC=C(NC(C)C)C=C5C)=O)CC4)=O)[C@]2(C)[C@H](C)CC3	4.01
O=C(CC1)[C@]2([H])[C@@]31[C@@H](C)[C@H](O)[C@](C)(C=C)C[C@@H](OC(CN4CCN(C(CN5CCN(C(CCl)=O)CC5)=O)CC4)=O)[C@]2(C)[C@H](C)CC3	4.01
O=C(CC1)[C@]2([H])[C@@]31[C@@H](C)[C@H](O)[C@](C)(C=C)C[C@@H](OC(CN4CCN(CC(NC5=CC=C(NCC=C)C=C5C)=O)CC4)=O)[C@]2(C)[C@H](C)CC3	4.01
O=C(CC1)[C@]2([H])[C@@]31[C@@H](C)[C@H](O)[C@](C)(C=C)C[C@@H](OC(CN4CCN(CC(NC5=CC=C([N+]([O-])=O)C=C5C)=O)CC4)=O)[C@]2(C)[C@H](C)CC3	4.00
O=C(CC1)[C@]2([H])[C@@]31[C@@H](C)[C@H](O)[C@](C)(C=C)C[C@@H](OC(CN4CCN(CC(NC5=CC=C(NCC)C=C5C)=O)CC4)=O)[C@]2(C)[C@H](C)CC3	4.00
O=C(CC1)[C@]2([H])[C@@]31[C@@H](C)[C@H](O)[C@](C)(C=C)C[C@@H](OC(CN4CCN(CC(NC5=CC=C(NC)C=C5C)=O)CC4)=O)[C@]2(C)[C@H](C)CC3	3.99
O=C(CC1)[C@]2([H])[C@@]31[C@@H](C)[C@H](O)[C@](C)(C=C)C[C@@H](OC(CN4CCN(CC(NC5=CC=C(N)C=C5C)=O)CC4)=O)[C@]2(C)[C@H](C)CC3	3.98
CC1C23C(C(CC3)=O)C(C(CC(C=C)(C)C1O)OC(CN4CCN(C(CCl)=O)CC4)=O)(C(CC2)C)C	3.91
O=C(CC1)[C@]2([H])[C@@]31[C@@H](C)[C@H](O)[C@](C)(C=C)C[C@@H](OC(CN4CCN(C5=CC=C(C(/C=C/C6=CC=C(C=C6)OC)=O)C=C5)CC4)=O)[C@]2(C)[C@H](C)CC3	3.73
O=C(CC1)[C@]2([H])[C@@]31[C@@H](C)[C@H](O)[C@](C)(C=C)C[C@@H](OC(CN4CCN(C5=CC=C(C(/C=C/C6=CC=CS6)=O)C=C5)CC4)=O)[C@]2(C)[C@H](C)CC3	3.71
O=C(CC1)[C@]2([H])[C@@]31[C@@H](C)[C@H](O)[C@](C)(C=C)C[C@@H](OC(CN4CCN(C5=CC=C(C(/C=C/C6=CC=CC=C6)=O)C=C5)CC4)=O)[C@]2(C)[C@H](C)CC3	3.71
O=C(CC1)[C@]2([H])[C@@]31[C@@H](C)[C@H](O)[C@](C)(C=C)C[C@@H](OC(CN4CCN(C5=CC=C(C(/C=C/C6=CC=CO6)=O)C=C5)CC4)=O)[C@]2(C)[C@H](C)CC3	3.70
O=C(CC1)[C@]2([H])[C@@]31[C@@H](C)[C@H](O)[C@](C)(C=C)C[C@@H](OC(CNCCCCCCCCCCCCCCCC)=O)[C@]2(C)[C@H](C)CC3	3.67
O=C(CC1)[C@]2([H])[C@@]31[C@@H](C)[C@H](O)[C@](C)(C=C)C[C@@H](OC(CN4CCN(C(CN5CCNCC5)=O)CC4)=O)[C@]2(C)[C@H](C)CC3	3.65
O=C1CC[C@]2(CC[C@H]3C)[C@]1([H])[C@]3(C)[C@H](OC(CN4CCN(C5=CC=CC=C5OC)CC4)=O)C[C@@](C)(C=C)[C@@H](O)[C@@H]2C	3.64
O=C(CC1)[C@]2([H])[C@@]31[C@@H](C)[C@H](O)[C@](C)(C=C)C[C@@H](OC(CNCCCCCCCCCCCC)=O)[C@]2(C)[C@H](C)CC3	3.63
O=C(CC1)[C@]2([H])[C@@]31[C@@H](C)[C@H](O)[C@](C)(C=C)C[C@@H](OC(CS(S(C4=NNC(N)=N4)=O)=O)=O)[C@]2(C)[C@H](C)CC3	3.63
O=C1CC[C@]2(CC[C@H]3C)[C@]1([H])[C@]3(C)[C@H](OC(CN4CCN(C5=CC=CC=C5C)CC4)=O)C[C@@](C)(C=C)[C@@H](O)[C@@H]2C	3.62
O=C(CC1)[C@]2([H])[C@@]31[C@@H](C)[C@H](O)[C@](C)(C=C)C[C@@H](OC(CS(S(C4=NNC=N4)=O)=O)=O)[C@]2(C)[C@H](C)CC3	3.61
O=C(CC1)[C@]2([H])[C@@]31[C@@H](C)[C@H](O)[C@](C)(C=C)C[C@@H](OC(CN(CC(C)C)CC(C)C)=O)[C@]2(C)[C@H](C)CC3	3.58
O=C(CC1)[C@]2([H])[C@@]31[C@@H](C)[C@H](O)[C@](C)(C=C)C[C@@H](OC(CNCCCCCCCC)=O)[C@]2(C)[C@H](C)CC3	3.58

Table S3. Summary of 51 compounds for 3D-QSAR model construction.

SMILES	pMIC	3D-QSAR Set
O=C(CC1)[C@]2([H])[C@@]31[C@@H](C)[C@H](O)[C@](C)(C=C)C[C@@H](OC(CSCC4=CSC(NC(C5=CC=NC=C5)=O)=N4)=O)[C@]2(C)[C@H](C)CC3	8.2	Training
O=C(CC1)[C@]2([H])[C@@]31[C@@H](C)[C@H](O)[C@](C)(C=C)C[C@@H](OC(CSCC4=CSC(NC(C5=CC=CC(N)=C5)=O)=N4)=O)[C@]2(C)[C@H](C)CC3	7.9	Test
O=C(CC1)[C@]2([H])[C@@]31[C@@H](C)[C@H](O)[C@](C)(C=C)C[C@@H](OC(CN4CCN(C5=CC=C(Cl)C=C5)CC4)=O)[C@]2(C)[C@H](C)CC3	7.57	Test
O=C(CC1)[C@]2([H])[C@@]31[C@@H](C)[C@H](O)[C@](C)(C=C)C[C@@H](OC(CN4N=NC(C5=CC=C(CN6C7=NC=NC(N)=C7N=C6)C=C5)=C4)=O)[C@]2(C)[C@H](C)CC3	7.34	Training
O=C(CC1)[C@]2([H])[C@@]31[C@@H](C)[C@H](O)[C@](C)(C=C)C[C@@H](OC(CN4CCN(C5=CC=CC(Cl)=C5)CC4)=O)[C@]2(C)[C@H](C)CC3	7.27	Training
O=C(CC1)[C@]2([H])[C@@]31[C@@H](C)[C@H](O)[C@](C)(C=C)C[C@@H](OC(CSC4=CC(CO)=CN=C4)=O)[C@]2(C)[C@H](C)CC3	7.03	Test
O=C1CC[C@]2(CC[C@H]3C)[C@]1([H])[C@]3(C)[C@H](OC(CSC4=NC(NC=C5)=C5C(NC(CN6CCCC6)=O)=N4)=O)C[C@@](C)(C=C)[C@@H](O)[C@@H]2C	7.01	Training
O=C(CC1)[C@]2([H])[C@@]31[C@@H](C)[C@H](O)[C@](C)(C=C)C[C@@H](OC(CSC4=NN=C(C5=CC=CC(Cl)=C5)N4)=O)[C@]2(C)[C@H](C)CC3	6.96	Training
O=C(CC1)[C@]2([H])[C@@]31[C@@H](C)[C@H](O)[C@](C)(C=C)C[C@@H](OC(CS(SC4=NN=C(C)S4)=O)=O)[C@]2(C)[C@H](C)CC3	6.95	Training
O=C(CC1)[C@]2([H])[C@@]31[C@@H](C)[C@H](O)[C@](C)(C=C)C[C@@H](OC(CN4CCN(C5=CC=CC=C5C)CC4)=O)[C@]2(C)[C@H](C)CC3	6.93	Training
O=C(CC1)[C@]2([H])[C@@]31[C@@H](C)[C@H](O)[C@](C)(C=C)C[C@@H](OC(CSC4=CC(NC(CN5CC(CO)CCC5)=O)=CN=C4)=O)[C@]2(C)[C@H](C)CC3	6.83	Test
O=C(CC1)[C@]2([H])[C@@]31[C@@H](C)[C@H](O)[C@](C)(C=C)C[C@@H](OC(CSC4CCN(C(CCN5C=NC6=C5N=C(N)N=C6N)=O)CC4)=O)[C@]2(C)[C@H](C)CC3	6.83	Training
O=C(CC1)[C@]2([H])[C@@]31[C@@H](C)[C@H](O)[C@](C)(C=C)C[C@@H](OC(CSC4=C(NC(CN5N=NC(CN6CCN(C7=CC=C([N+]([O-])=O)C=C7)CC6)=C5)=O)C=CC=C4)=O)[C@]2(C)[C@H](C)CC3	6.81	Training
O=C(CC1)[C@]2([H])[C@@]31[C@@H](C)[C@H](O)[C@](C)(C=C)C[C@@H](OC(CSC4=C(NC(CN5N=NC(CN6CCN(C7=CC=CC([N+]([O-])=O)=C7)CC6)=C5)=O)C=CC=C4)=O)[C@]2(C)[C@H](C)CC3	6.81	Training
O=C(CC1)[C@]2([H])[C@@]31[C@@H](C)[C@H](O)[C@](C)(C=C)C[C@@H](OC(N4C(C(C=C(N)C=C5)=C5C4)=O)=O)[C@]2(C)[C@H](C)CC3	6.72	Training
O=C(CC1)[C@]2([H])[C@@]31[C@@H](C)[C@H](O)[C@](C)(C=C)C[C@@H](OC(CSC4=NN=C(C5=CC=C([N+]([O-])=O)C=C5)N4)=O)[C@]2(C)[C@H](C)CC3	6.67	Training
O=C(CC1)[C@]2([H])[C@@]31[C@@H](C)[C@H](O)[C@](C)(C=C)C[C@@H](OC(CSC4=NN=C(C5=CC=C(O)C=C5)O4)=O)[C@]2(C)[C@H](C)CC3	6.65	Training
O=C(CC1)[C@]2([H])[C@@]31[C@@H](C)[C@H](O)[C@](C)(C=C)C[C@@H](OC(CN4CCN(C5=CC=C(C)C=C5)CC4)=O)[C@]2(C)[C@H](C)CC3	6.63	Training
O=C(CC1)[C@]2([H])[C@@]31[C@@H](C)[C@H](O)[C@](C)(C=C)C[C@@H](OC(CSC4=NC=NC5=C4C=NN5)=O)[C@]2(C)[C@H](C)CC3	6.61	Training
O=C(CC1)[C@]2([H])[C@@]31[C@@H](C)[C@H](O)[C@](C)(C=C)C[C@@H](OC(CNC4=CC=CC(Cl)=C4)=O)[C@]2(C)[C@H](C)CC3	6.61	Training
O=C(CC1)[C@]2([H])[C@@]31[C@@H](C)[C@H](O)[C@](C)(C=C)C[C@@H](OC(CSC4=C([N+]([O-])=O)C=CC=N4)=O)[C@]2(C)[C@H](C)CC3	6.52	Training
O=C(CC1)[C@]2([H])[C@@]31[C@@H](C)[C@H](O)[C@](C)(C=C)C[C@@H](OC(CSC4=CC(NC([C@H]5CCCN5)=O)=CN=C4)=O)[C@]2(C)[C@H](C)CC3	6.49	Training
O=C1CC[C@]2(CC[C@H]3C)[C@]1([H])[C@]3(C)[C@H](OC(CSC4=NC(NC=C5)=C5C(NC(CN6CCC(CO)CC6)=O)=N4)=O)C[C@@](C)(C=C)[C@@H](O)[C@@H]2C	6.44	Training
O=C(CC1)[C@]2([H])[C@@]31[C@@H](C)[C@H](O)[C@](C)(C=C)C[C@@H](OC(CSC4=NN(C(C5=CC=C(OC)C=C5)=O)C(N)=N4)=O)[C@]2(C)[C@H](C)CC3	6.39	Training
O=C1CC[C@]2(CC[C@H]3C)[C@]1([H])[C@]3(C)[C@H](OC(CSC4=NC(N(C)C)=CC(C)=N4)=O)C[C@@](C)(C=C)[C@@H](O)[C@@H]2C	6.34	Training
O=C(CC1)[C@]2([H])[C@@]31[C@@H](C)[C@H](O)[C@](C)(C=C)C[C@@H](OC(CSC4=C(NC(CN5N=NC(CN6CCOCC6)=C5)=O)C=CC=C4)=O)[C@]2(C)[C@H](C)CC3	6.14	Training
O=C(CC1)[C@]2([H])[C@@]31[C@@H](C)[C@H](O)[C@](C)(C=C)C[C@@H](OC(CSCC4=CSC(NC(CN(CCC)CCC)=O)=N4)=O)[C@]2(C)[C@H](C)CC3	6.11	Training
O=C(CC1)[C@]2([H])[C@@]31[C@@H](C)[C@H](O)[C@](C)(C=C)C[C@@H](OC(N(CC4)C(C5=C4C=C(OCCN6CCCCC6)C=C5)=O)=O)[C@]2(C)[C@H](C)CC3	6.09	Training
O=C1CC[C@]2(CC[C@H]3C)[C@]1([H])[C@]3(C)[C@H](OC(CSCC(NC4=CC=C(NCC=C)C=C4C)=O)=O)C[C@@](C)(C=C)[C@@H](O)[C@@H]2C	6.08	Test
O=C(CC1)[C@]2([H])[C@@]31[C@@H](C)[C@H](O)[C@](C)(C=C)C[C@@H](OC(CS[C@H]4CC[C@H](C(OCC)=O)C[C@H]4NS(=O)(C5=CC=C(C#N)C=C5)=O)=O)[C@]2(C)[C@H](C)CC3	5.86	Training

O=C(CC1)[C@]2([H])[C@@]31[C@@H](C)[C@H](O)[C@](C)(C=C)C[C@@H](OC(CS[C@H]4CC[C@H](C(OCC)=O)C[C@H]4NS(=O)(C5=CC=C(C#N)C=C5)=O)=O)[C@]2(C)[C@H](C)CC3	5.86	Training
O=C(CC1)[C@]2([H])[C@@]31[C@@H](C)[C@H](O)[C@](C)(C=C)C[C@@H](OC(CN4CCN(C(NC5=CC=CC6=C5C=CC=N6)=O)CC4)=O)[C@]2(C)[C@H](C)CC3	5.79	Training
O=C(CC1)[C@]2([H])[C@@]31[C@@H](C)[C@H](O)[C@](C)(C=C)C[C@@H](OC(N4C(C(C=C(NC(C(O)=O)C(C)C)C=C5)=C5C4)=O)=O)[C@]2(C)[C@H](C)CC3	5.77	Test
O=C(CC1)[C@]2([H])[C@@]31[C@@H](C)[C@H](O)[C@](C)(C=C)C[C@@H](OC(CS[C@H]4CC[C@H](C(OCC)=O)C[C@H]4N)=O)[C@]2(C)[C@H](C)CC3	5.75	Test
O=C(CC1)[C@]2([H])[C@@]31[C@@H](C)[C@H](O)[C@](C)(C=C)C[C@@H](OC(CN4N=NC(CCCN5C(N=CN=C6N)=C6N=C5)=C4)=O)[C@]2(C)[C@H](C)CC3	5.48	Training
O=C1CC[C@]2(CC[C@H]3C)[C@]1([H])[C@]3(C)[C@H](OC(CSC4=NC(O)=CC(NC([C@H](C)C(C)C)=O)=N4)=O)C[C@@](C)(C=C)[C@@H](O)[C@@H]2C	5.48	Training
O=C1CC[C@]2(CC[C@H]3C)[C@]1([H])[C@]3(C)[C@H](OC(CSC4=NC(N)=CC(N4C)=O)=O)C[C@@](C)(C=C)[C@@H](O)[C@@H]2C	5.41	Training
O=C(CC1)[C@]2([H])[C@@]31[C@@H](C)[C@H](O)[C@](C)(C=C)C[C@@H](OC(CNCC(C)C)=O)[C@]2(C)[C@H](C)CC3	5.34	Test
O=C(CC1)[C@]2([H])[C@@]31[C@@H](C)[C@H](O)[C@](C)(C=C)C[C@@H](OC(CSC4CCN(C(CCN5C=NC6=C5N=C(N)N=C6N7CC(CNC)CC7)=O)CC4)=O)[C@]2(C)[C@H](C)CC3	5.29	Training
O=C(CC1)[C@]2([H])[C@@]31[C@@H](C)[C@H](O)[C@](C)(C=C)C[C@@H](OC(CN4CCN(C(CN5N=NC(CN6CCN(C7=CC(C)=CC=C7)CC6)=C5)=O)CC4)=O)[C@]2(C)[C@H](C)CC3	5.27	Training
O=C1CC[C@]2(CC[C@H]3C)[C@]1([H])[C@]3(C)[C@H](OC(CSC4=NC(O)=CC(NC([C@@H]5NCCCC5)=O)=N4)=O)C[C@@](C)(C=C)[C@@H](O)[C@@H]2C	5.19	Test
O=C(CC1)[C@]2([H])[C@@]31[C@@H](C)[C@H](O)[C@](C)(C=C)C[C@@H](OC(CSC4=NN=C(NC(C5=CC=CC=C5N)=O)S4)=O)[C@]2(C)[C@H](C)CC3	5.19	Test
O=C(CC1)[C@]2([H])[C@@]31[C@@H](C)[C@H](O)[C@](C)(C=C)C[C@@H](OC(CN4CCCCC4)=O)[C@]2(C)[C@H](C)CC3	5.05	Training
O=C(CC1)[C@]2([H])[C@@]31[C@@H](C)[C@H](O)[C@](C)(C=C)C[C@@H](OC(CN4CCN(C(CN5N=NC(CN6CCN(C7=CC=C(C)C=C7)CC6)=C5)=O)CC4)=O)[C@]2(C)[C@H](C)CC3	4.97	Test
O=C(CC1)[C@]2([H])[C@@]31[C@@H](C)[C@H](O)[C@](C)(C=C)C[C@@H](OC(CN4CCN(C(CN5N=NC(CN6CCN(C)CC6)=C5)=O)CC4)=O)[C@]2(C)[C@H](C)CC3	4.92	Training
O=C(CC1)[C@]2([H])[C@@]31[C@@H](C)[C@H](O)[C@](C)(C=C)C[C@@H](OC(CSC(C)(C)CNC(C4=CC(Cl)=CC=C4)=O)=O)[C@]2(C)[C@H](C)CC3	4.88	Training
O=C(CC1)[C@]2([H])[C@@]31[C@@H](C)[C@H](O)[C@](C)(C=C)C[C@@H](OC(CN(CC)CC)=O)[C@]2(C)[C@H](C)CC3	4.73	Test
O=C(CC1)[C@]2([H])[C@@]31[C@@H](C)[C@H](O)[C@](C)(C=C)C[C@@H](OC(CN4CCN(C(CN5N=NC(CN6CCN(N7CCC(O)CC7)CC6)=C5)=O)CC4)=O)[C@]2(C)[C@H](C)CC3	4.67	Test
O=C1CC[C@]2(CC[C@H]3C)[C@]1([H])[C@]3(C)[C@H](OC(CSC4=NC(O)=CC(NC(C5=CC=CC(Cl)=C5)=O)=N4)=O)C[C@@](C)(C=C)[C@@H](O)[C@@H]2C	4.3	Training
O=C(CC1)[C@]2([H])[C@@]31[C@@H](C)[C@H](O)[C@](C)(C=C)C[C@@H](OC(CSCC4=CSC(NC(CN5C=CN=N5)=O)=N4)=O)[C@]2(C)[C@H](C)CC3	4.28	Test
O=C(CC1)[C@]2([H])[C@@]31[C@@H](C)[C@H](O)[C@](C)(C=C)C[C@@H](OC(CNCCCCCCCCCCCC)=O)[C@]2(C)[C@H](C)CC3	4.23	Test

Table S4. Summary of experimental versus predictive activity(3D-QSAR).

Name	Type	Experimental Activity	Predictive Activity	Residual
molec1	Training	8.2	8.28	−0.08
molec2	Test	7.9	7.87	0.03
molec3	Training	7.27	7.16	0.11
molec4	Training	6.95	6.98	−0.03
molec5	Training	6.96	7.08	−0.12
molec6	Training	7.01	7.02	−0.01
molec7	Training	6.72	6.74	−0.02
molec8	Training	6.83	6.97	−0.14
molec9	Test	6.83	7.20	−0.37
molec10	Training	6.61	6.73	−0.12
molec11	Training	6.65	6.51	0.14
molec12	Training	6.61	6.75	−0.14
molec13	Training	6.81	6.74	0.07
molec14	Training	6.81	6.71	0.10
molec15	Training	6.67	6.58	0.09
molec16	Training	6.63	6.64	−0.01
molec17	Training	6.52	6.53	−0.01

molec18	Training	6.49	6.62	−0.13
molec19	Training	6.34	6.27	0.07
molec20	Training	6.44	6.46	−0.02
molec21	Training	6.11	6.12	−0.01
molec22	Training	6.14	6.10	0.04
molec23	Training	5.86	5.91	−0.05
molec24	Training	5.86	5.72	0.14
molec25	Test	5.77	6.46	−0.69
molec26	Training	5.48	5.39	0.09
molec27	Training	5.41	5.44	−0.03
molec28	Training	5.05	5.30	−0.25
molec29	Training	5.29	5.23	0.06
molec30	Training	5.27	5.29	−0.02
molec31	Test	4.97	5.67	−0.70
molec32	Training	4.92	5.03	−0.11
molec33	Test	4.73	5.07	−0.34
molec34	Training	6.39	6.33	0.06
molec35	Test	4.28	4.78	−0.50
molec36	Training	4.3	4.46	−0.16
molec37	Test	7.57	7.17	0.40
molec38	Training	7.34	7.32	0.02
molec39	Test	7.03	6.73	0.30
molec40	Training	6.93	6.73	0.20
molec41	Test	6.08	6.24	−0.16
molec42	Training	6.09	5.96	0.13
molec43	Test	5.75	5.85	−0.10
molec44	Training	5.79	5.85	−0.06
molec45	Training	5.48	5.34	0.14
molec46	Test	5.34	6.37	−1.03
molec47	Test	5.19	6.29	−1.10
molec48	Test	5.19	5.84	−0.65
molec49	Training	4.88	4.80	0.08
molec50	Test	4.67	5.22	−0.55
molec51	Test	4.23	5.45	-1.22
